# A phylogenomic and ecological analysis of the globally abundant Marine Group II archaea (*Ca*. Poseidoniales ord. nov.)

**DOI:** 10.1038/s41396-018-0282-y

**Published:** 2018-10-15

**Authors:** Christian Rinke, Francesco Rubino, Lauren F. Messer, Noha Youssef, Donovan H. Parks, Maria Chuvochina, Mark Brown, Thomas Jeffries, Gene W. Tyson, Justin R. Seymour, Philip Hugenholtz

**Affiliations:** 1 Australian Centre for Ecogenomics, School of Chemistry and Molecular Biosciences, The University of Queensland, St. Lucia, QLD Australia; 2 Department of Microbiology and Molecular Genetics, Oklahoma State University Stillwater, Stillwater, OK USA; 3 School of Environmental and Life Sciences, University of Newcastle, Callaghan, NSW Australia; 4 Hawkesbury Institute for the Environment, Western Sydney University, Sydney, NSW Australia; 5 Climate Change Cluster, University of Technology Sydney, Sydney, NSW Australia

**Keywords:** Environmental microbiology, Phylogenetics, Metagenomics

## Abstract

Marine Group II (MGII) archaea represent the most abundant planktonic archaeal group in ocean surface waters, but our understanding of the group has been limited by a lack of cultured representatives and few sequenced genomes. Here, we conducted a comparative phylogenomic analysis of 270 recently available MGII metagenome-assembled genomes (MAGs) to investigate their evolution and ecology. Based on a rank-normalised genome phylogeny, we propose that MGII is an order-level lineage for which we propose the name *Candidatus* Poseidoniale*s* (after Gr. n. Poseidon, God of the sea), comprising the families *Candidatus* Poseidonaceae fam. nov. (formerly subgroup MGIIa) and *Candidatus* Thalassarchaeaceae fam. nov. (formerly subgroup MGIIb). Within these families, 21 genera could be resolved, many of which had distinct biogeographic ranges and inferred nutrient preferences. Phylogenetic analyses of key metabolic functions suggest that the ancestor of *Ca*. Poseidoniales was a surface water-dwelling photoheterotroph that evolved to occupy multiple related ecological niches based primarily on spectral tuning of proteorhodopsin genes. Interestingly, this adaptation appears to involve an overwrite mechanism whereby an existing single copy of the proteorhodopsin gene is replaced by a horizontally transferred copy, which in many instances should allow an abrupt change in light absorption capacity. Phototrophy was lost entirely from five *Ca*. Poseidoniales genera coinciding with their adaptation to deeper aphotic waters. We also report the first instances of nitrate reductase in two genera acquired via horizontal gene transfer (HGT), which was a potential adaptation to oxygen limitation. Additional metabolic traits differentiating families and genera include flagellar-based adhesion, transporters, and sugar, amino acid, and peptide degradation. Our results suggest that HGT has shaped the evolution of *Ca*. Poseidoniales to occupy a variety of ecological niches and to become the most successful archaeal lineage in ocean surface waters.

## Introduction

Following the original discovery of marine planktonic Archaea, first in surface and then in deep ocean waters [1, 2], members of this domain are now known to be ubiquitous and abundant in the marine environment [3]. Currently marine archaea are classified into three major groups; Thaumarchaeota (formerly Marine Group I archaea), Marine Group II archaea (MGII) and Marine Group III archaea (MGIII), with the latter two groups currently lacking cultured representatives and being related to *Thermoplasma* in the phylum Euryarchaeota [1, 4]. MGII has been divided into two subgroups, which became known as IIa and IIb [4, 5]. Common features of MGII include a preference for the photic zone with a lower abundance in the deep sea, pronounced seasonal and spatial variations, and a worldwide distribution including polar regions [6]. Proteorhodopsin genes were identified on MGII genome fragments obtained from oceanic surface water fosmid libraries suggesting a photoheterotrophic lifestyle for some members of this group [7]. By contrast, proteorhodopsin genes were not detected in deep-sea MGII genome fragments suggesting a chemoheterotrophic lifestyle based on inferred amino acid, carbohydrate, and lipid metabolism [8]. The most complete MGII genome obtained to date is a member of subgroup IIa recovered from a Puget Sound surface water metagenome. A metabolic reconstruction predicted a motile photoheterotroph, capable of degrading proteins and lipids, potentially indicating organic particles as important growth substrates [9]. Two draft genomes (fosmid clones) were subsequently obtained from the deep chlorophyll maximum layer (50 m) in the Mediterranean Sea and assigned to subgroup IIb, for which the authors proposed the class Thalassoarchaea [10]. The genomes indicated a photoheterotrophic lifestyle, diversified substrate degrading capabilities and a lack of motility. Despite these first glimpses into the metabolic potential of MGII, a comprehensive genomic analysis spanning a broad representation of this group is currently lacking. Here we report an analysis of 270 MGII metagenome-assembled genomes (MAGs) focusing on their phylogeny, global distribution and metabolic potential to better understand the role of this group in marine ecosystems. We propose the name *Candidatus* Poseidoniales (ord. nov.) and subordinate taxa using MAG type species and a rank-normalised taxonomy. Analysis of spatial and temporal distribution patterns in correlation with physico-chemical metadata suggest genus- and family-specific lifestyle and niche partitioning. Metabolic reconstruction predicts an exclusively heterotrophic lifestyle and ancestral phototrophy with spectral tuning of proteorhodopsin genes likely obtained via lateral transfer from bacterial donors.

## Materials and methods

### Sampling site

Seawater samples were collected monthly (July 2012–September 2013) from the Australian Integrated Marine Observing System (IMOS) National Reference Station (NRS), Port Hacking, situated in western Tasman Sea waters ~8 km off the coast of Sydney, Australia (34° 4′ S, 151° 13′ E). Sampling was conducted on the F/V *Zelda Faith II* and comprised the collection of 10 L of seawater from 1 m below the surface using a plastic bucket. Seawater was immediately processed on-board the vessel via inline filtration through a glass fibre pre-filter (1.6 μm nominal pore size) onto a 0.22 μm polyethersulfone membrane (Millipore® Sterivex™) filter unit, using a peristaltic pump (Whatson-Marlow). After filtration, samples were snap frozen in liquid nitrogen and then stored at −80 °C until extraction. DNA was extracted from all samples using the MoBio PowerWater DNA Isolation kit according to the manufacturer’s instructions with the following modifications: Sterivex filters were aseptically removed from their plastic casing using a sterile scalpel, filters were then transferred to the PowerWater bead beating tubes and incubated at 60 °C for 10 min with solution PW1 to ensure complete cell lysis. DNA concentration was quantified using the Qubit Fluorometer (Invitrogen), Nextera metagenomic libraries were prepared for each sample followed by Illumina HiSeq shotgun sequencing (2 × 100 bp reads) allocating 1/3 of a lane for each sample.

### Metagenome-assembled genomes (MAGs)

The majority of the 285 genomes (Table S1) examined for use in the present study have been previously reported [9–13] (see introduction). In addition, 16 MAGs were obtained from the Port Hacking National Reference Station (see above) using the following workflow. Raw sequencing reads were adapter clipped and quality trimmed with Trimmomatic v0.32 [14] with Nextera adapter sequences. BBMerge (version BBMAP: bbmap_34.94; https://sourceforge.net/projects/bbmap) was used to merge overlapping pairs of reads using default parameters. Quality-controlled paired reads were assembled with CLC Genomics Cell assembler v4.4 using an estimated insert size of 30–500 bp. Quality-controlled paired reads were mapped to the assembled contigs using BamM v1.5.0 (http://ecogenomics.github.io/BamM/) that employs BWA [15]. Binning was performed with MetaBAT v2.12 [16], and all Port Hacking MAGs were screened for an estimated completeness >50% and estimated contamination <1% with CheckM v1.0.5 [17]. Raw and processed sequence data of the 15 Port Hacking MAGs, and of 46 MAGs recovered by Parks et al. [18] from public data sets but not included in the final manuscript, were deposited in the NCBI BioProject database ID: PRJNA481422.

### Refining completeness and contamination estimates

The completeness and contamination estimates provided by CheckM are based on the presence of predefined lineage-specific marker sets [17]. These marker sets can be further refined using checkm qa --out_format 4 (list of marker genes and their counts), which allows screening a lineage, in our case *Candidatus* Poseidoniales (MG II), to identify markers which are absent from all MAGs in the lineage. Based on this workflow we excluded 11 marker genes (TIGR00537, TIGR02237, TIGR00422, PF01287.15, TIGR03677, PF09249.6, PF13685.1, TIGR00162, PF02649.9, PF03684.8, PF01849.13) that were absent across all 270 *Ca*. Poseidoniales MAGs, resulting in 177 marker genes being used for completeness and contamination estimates.

### SSU phylogeny

The SSU database (SSURef_Nr99_123.1) for ARB [19] was obtained from the SILVA rRNA database project (https://www.arb-silva.de/). SSU rRNA gene sequences from *Ca*. Poseidoniales (MGII) MAGs were obtained from the Genome Taxonomy Database (GTDB; http://gtdb.ecogenomic.org). Initially sequences with a minimum length of 200 bp were selected, aligned using the online SINA aligner (https://www.arb-silva.de/), imported into ARB and added to the SILVA tree included in the SSURef_Nr99_123.1 ARB package, using the ARB Parsimony (Quick add marked) function. Next a bootstrapped phylogenetic tree of *Thermoplasmata* was calculated, using 3694 SSU sequences, 43 of which were SSU rRNA sequences (>700 bp) extracted from MGII MAGs. First a snapshot tree was calculated with FastTree v2.1.9 (CAT; WAG) [20], then support values were established using 100 nonparameteric bootstrap trees. Subsequently a maximum likelihood tree was calculated with FastTree 2.1.9 (CAT, WAG) [20] using 43 SSU rRNA sequences (>700 bp) extracted from MGII MAGs; 10 reference sequences used in Galand et al. [5] obtained from NCBI and SILVA, and 9 outgroup (E2, MGIII) sequences, resulting in 62 sequences in total. The tree was rooted and 24 MGII SSU sequences greater than 500 bp and smaller than 700 bp were added using the parsimony quick add tool in ARB.

### Genome phylogeny

We inferred maximum likelihood trees based on a multiple sequence alignment of 122 single-copy marker proteins (Table S2). The 122 archaeal proteins were identified as being present in ≥90% of bacterial or archaeal genomes and, when present, single-copy in ≥95% of genomes [18]. Proteins were aligned to Pfam and TIGRfam hidden Markov models (HMMs) using HMMER61 v.3.1b1 with default parameters and trees were inferred with FastTree 2 v.2.1.7 under the WAG + GAMMA model [18].

### Rank normalisation

The assignment of all taxonomic ranks is based on relative evolutionary divergence (RED) [11] calculated from the 122 single-copy marker protein tree using PhyloRank (v0.0.27; https://github.com/dparks1134/PhyloRank). The RED approach ensures that taxa at the same taxonomic rank diverged at similar times. This is achieved by setting the RED value of the last common ancestor occurring at a fixed time in the past to zero (RED = 0), all taxa existing in the present to one (RED = 1), and by linearly interpolating RED values of internal nodes between these values according to lineage-specific rates of evolution [11]. Polyphyletic MGII clades and clades with RED values too low for the genus rank (±0.1) were split into multiple groups and RED values were recalculated to achieve a RED value well within the genus rank range.

### Proposed type species

We proposed two type species based on MAGs employing the following rules: (1) high-quality draft MAG criteria must be met (>90% completion; <5% contamination; multiple fragments where gaps span repetitive regions; presence of the 23S, 16S, and 5S rRNA genes and at least 18 tRNAs; Bowers et al. [21]); (2) near full-length rRNA genes must be present with a threshold of 1200 bp for 16S rRNA genes; and 1900 bp for 23S rRNA genes; and 100 bp for 5S rRNA genes; (3) assembly must consist of 100 contigs or less with less than ten ambiguous bases. All MAGs meeting these criteria were then ranked by quality score integers (completeness-4× contamination) and the highest-ranking genome of MGIIa and MGIIb was selected as a type species.

### Abundance correlation analysis

#### Tara Ocean data set

Reads from 238 samples (332 runs) of Tara Ocean metagenomes [22] were mapped against a de-replicated set of 204 MAGs using BamM v1.5.0 (http://ecogenomics.github.io/BamM/) that employs BWA [15]. Metadata were extracted from Tara Oceans samples using Table W1 “Tara Ocean sample description” [22]. Out of a total of 238 samples, 95 were excluded because they accounted for less than 5% of the whole data, leaving 143 samples that were first scaled by library size, using functions from MGKit v0.3.1 (https://bitbucket.org/setsuna80/mgkit), which implements the methodology in Anders [23]. A negative binomial (NB) generalised linear model (GLM) was fit for each clade against each of the variables in the metadata separately, using statsmodels v0.8.0 (www.statsmodels.org). Categorical variables were coded using the formulas implemented in patsy (https://github.com/pydata/patsy) as “Treatment” for “Environmental Feature” and “Super Marine Biomes”. The reference used were “‘(MES) mesopelagic zone”, “0.22–0.45” and “Coastal Biome” respectively. “Deviation coding (Sum)” was used for the rest of the categorical variables, with the omitted values of the choice of “reference” (for Treatment coding) and “omit” (for Deviation Coding) values decided upon by fitting models with all possible values and minimising the Akaike’s Information Criterion (AIC) [24] of the models. Furthermore, observations containing missing data for a variable were dropped previous to the model fit. Moreover, the alpha parameter of the NB was optimised using the functions available in scipy (www.scipy.org), by minimising the AIC of each fit. A Wald test, qs implemented in statsmodels, was then performed on each fit to assess the significance of the independent variable, followed by correction of the *p* values using the Benjamini−Hochberg (BH) method [25], thereafter accepting a significance threshold of *p* < 0.1 for a variable, and of *p* < 0.05 for its coefficients.

#### Port Hacking data set

Reads from 15 Port Hacking metagenomes (see above) were mapped against a de-replicated set of 204 MAGs as described above. The time series data were analysed using a generalised additive model (GAM), with an NB family and a log link. Cubic splines were used to model the natural community complexity, as implemented in “statsmodels”. As in the previous section the alpha parameter of NB, as well as the degrees of freedom (ranging from 3 to 9) of the splines were determined for each fit by minimising its AIC. Thereafter the confidence intervals were assessed and trends examined to identify variables that carried specific importance to a clade. Moreover, only regions with at least one clade showing significantly different abundances from the mean of means (grand mean; baseline) were included in the results (10.17605/OSF.IO/PAQ3F).

### Metabolic reconstruction

Gene calling was performed with Prokka v1.2 [26], and the resulting protein fasta files were searched against uniref100 [27], accessed 2015.10.20, using Diamond’s v0.7.12 [28] blastx option. The top hit of each read (if above 1e-3) was mapped to Kegg Orthology (KO) IDs [29] using the Uniprot ID mapping files. Hits to each KO were summed to produce a count table. Assignments of key functions were manually verified by inspecting gene feature format (gff) files with CLC Genomics Workbench v10.0.0.64 (https://www.qiagenbioinformatics.com).

#### Peptidases

Peptidase encoding genes were assigned by blasting the Prokka output of all MAGs against the MEROPS database [30] file “merops_scan.lib”. This file contains a nonredundant library of protein sequences including peptidase units from all family types.

#### Gene phylogenies

First IMG [31] was screened for genes of interest and the amino acid sequences were exported as protein FASTA files. Mingle (https://github.com/Ecogenomics/mingle) was used to identify homologues (references) across the GTDB r76 (http://gtdb.ecogenomic.org) [18]. Next GraftM v0.10.1 [32] was employed to create gene-specific HMMs which were then run against all MAGs and references. The recovered aligned and trimmed sequences were manually curated in ARB, exported, and used to calculate a bootstrapped maximum likelihood tree (100 replicates) with FastTree 2 (gamma, wag) [20] and GeneTreeTk v0.0.11 (https://github.com/dparks1134/GeneTreeTk). For the proteorhodopsin genes we employed a specific alignment filter (30% minimum similarity, termini 113 to 585) when exporting protein sequences from ARB for tree calculations.

#### Size fraction

In total, 15 size fractioned metagenomes published previously [33] were obtained from the authors and blasted (blastx) against the Prokka output (*.faa files) of all MAGs and the best blast hit was extracted. Flagella protein abundances were normalised by five single-copy ribosomal genes (S4, S15, S24e, S27ae, L21).

## Results and discussion

### MGII genome data set

A total of 285 genomes belonging to MGII obtained from 141 marine metagenomes were examined in the present study (Table S1). The majority of these have been previously reported, including 234 MAGs mined from public metagenomes [11], 24 from deep-sea samples ranging from 800 to 4950 m [12], nine from the Red Sea [13], and one from surface waters of Puget Sound [9], as well as two partial genomes present in a fosmid library from the Mediterranean deep chlorophyll maximum layer [10]. An additional 16 MAGs were obtained from an oceanographic reference station, situated in coastal waters of the Tasman Sea, sequenced as part of the present study. Based on an initial quality screening we excluded 15 of the 285 genomes from the analysis (Table S1), due to either low estimated completeness (<20%) and/or high estimated contamination (>10%). The low stringency completeness threshold allowed two partially complete deep-sea MAGs [12] to be included in the study (Table S1). The average estimated completeness and contamination of the remaining 270 MAGs used for all subsequent analyses was 77.7 ± 9.2% and 1.0 ± 1.4%, respectively. Estimated MAG genome sizes ranged from 1.5 to 3.3 Mbp (average 2.1 ± 0.3 Mbp) with a genomic GC content from 34.5 to 62.7% (Fig. S1).

### Phylogenetic analysis

**Fig. 1:**
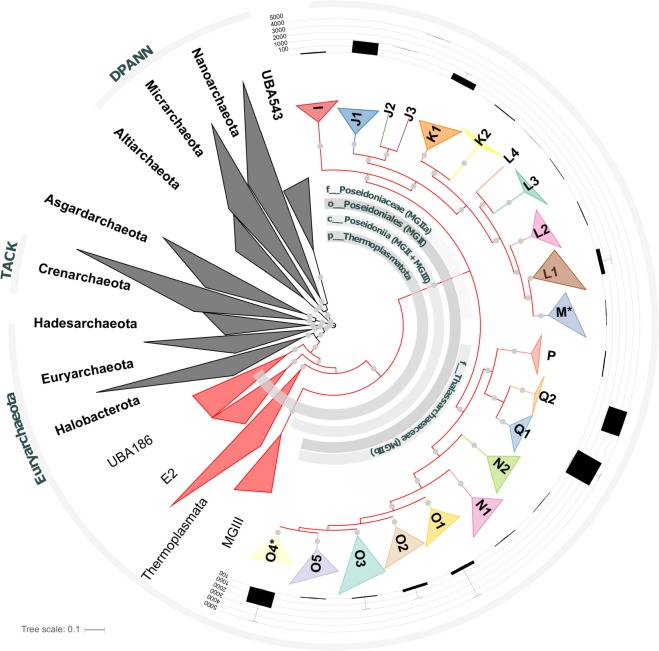
Phylogenomic inference of Marine Group II MAGs. The phylogenomic tree is based on 122 concatenated archaeal marker gene alignments. Inner grey arcs indicate the breadth of the proposed families *Ca*. Poseidoniaceae fam. nov. (MGIIa) and *Ca*. Thalassarchaeaceae fam. nov. (MGIIb), the order *Ca*. Poseidoniales ord. nov. (MGII), the class *Ca*. Poseidoniia class nov. (MGII and MGII), and the phylum *Thermoplasmatota*. The families *Ca*. Poseidoniaceae include the genera (former clades) I to M and *Ca*. Thalassarchaeaceae the genera (clades) N1–Q2, respectively. Genera containing type material are indicated by an asterisk. Sampling depths of metagenomes from which MAGs were derived are displayed via box whisker plots for each genus (box shows first and third quartile; whisker indicate minimum and maximum depth values), and major archaeal groups (former superphyla) including Euryarchaeota, TACK, and DPANN are indicated by outer grey arcs. Bootstrap values over 90% are indicated by grey filled circles. The scale bar represents 0.1 substitutions per site

Concatenated alignments of multiple universally distributed single-copy marker genes are considered to provide better phylogenetic resolution than single gene-based phylogenies [34]. We therefore inferred maximum likelihood trees using a multiple sequence alignment of 122 single-copy archaeal marker genes [11] (Table S2), confirming that all 270 MAGs belong to Marine Group II (Fig. S2). This placement is broadly consistent with the small subunit (SSU) ribosomal RNA gene tree, which placed 67 SSU rRNA sequences (>500 bp) obtained from the MAG data set within MGII; however, many associated groups exclusively comprising environmental sequences have yet to be linked to genome sequences (Fig. S3). MAGs were further classified into subgroups IIa and IIb and clades therein (Fig. 1) based on a previously proposed SSU rRNA classification [5] (Fig S4). In order to assign taxonomic ranks to these groups, we used a recently proposed method, which normalises ranks based on relative evolutionary divergence (RED) [18]. Using this metric, MGII is best described as an order-level group, subgroups IIa and IIb as families, and subordinate clades as genera (Fig. S5). However, two of the clades originally defined by Galand et al. [5] (L and N) were polyphyletic in the genome tree and were subdivided to establish monophyly (L1–4, N1-2; Fig. 1). A further four clades (J, K, O and Q) were deeply divergent and had to be subdivided to have RED values consistent with the rank of genus (J1-3, K1-2, O1-5, Q1-2) resulting in a total of 21 genera (Fig. 1, Fig. S5). A number of changes were also required for the higher ranks circumscribing MGII. Based on SSU rRNA taxonomy, the MGII are currently classified within the class *Thermoplasmata* [35]. However, we propose that the *Thermoplasmata* should be reclassified as a phylum according to RED analysis, which also necessitates a new class circumscribing MGII and MGIII (Fig. S1, Fig. S5). We propose formal names for these groups including type species below.

### Proposal of type material and higher ranks

The rank-normalised phylogenetic analysis indicates that a number of taxonomic changes are required to accommodate the reclassification of MGII. Several authors have proposed the use of genome sequences from as-yet uncultured microorganisms as type material for defining species and higher taxa [36–38]. Following these recommendations, we propose two type species based on two of the 270 MAGs used in the present study; *Candidatus* Poseidonia alphae (GCA_002505405.1) representing subgroup IIa and *Candidatus* Thalassarchaeum betae (PSPG00000000) representing subgroup IIb (Fig. S6; Suppl. Text). Based on these type species, we further propose the families *Candidatus* Poseidoniaceae fam. nov. and *Candidatus* Thalassarchaeaceae fam. nov., the order *Candidatus* Poseidoniales ord. nov. and the class *Candidatus* Poseidoniia class nov. (Fig. 1; Suppl. Text).

### Distribution and lifestyle

**Fig. 2:**
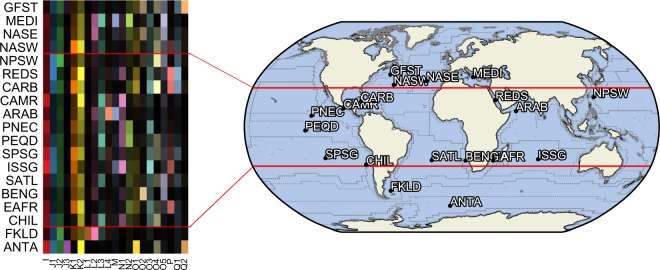
Global distribution of *Ca*. Poseidoniales genera. Shown are the relative abundances based on read mapping of the de-replicated 204 MAGs reference set. *Heatmap* (on left) of normalised genera abundances (*x*-axis) versus Ocean regions (*y*-axis). Darker colours indicate a lower abundance in Ocean regions for a genus, while brighter colours indicate a higher abundance, hue is assigned by genus. Note that only regions with at least one genus showing significantly different abundances from the mean of means (grand mean; baseline) were included in the heatmap. Also based on AIC (Akaike information criterion) model selection, the region “Indian Monsoon Gyres Province” (MONS) was omitted from the sum-to-zero coding-based generalized linear model shown in the heatmap. *World map* (on right) shows the geographic position of each Ocean region. Points and acronyms of the mapped Ocean regions were assigned according to Longhurst’s classification [64] (http://www.marineregions.org) with points indicating the centroids of the polygons defining each region. The acronyms are the following: Gulf Stream (GFST), Mediterranean Sea (MEDI), Northeast Atlantic subtropical gyre (NASE), Northwest Atlantic subtropical gyre (NASW), Northwest Pacific subtropical (NPSW), Caribbean (CARB), Central American coast (CAMR), Northwestern Arabian Upwelling (ARAB), North Pacific equatorial counter current (PNEC), Indian monsoon gyre (MONS), Pacific Equatorial Divergence (PEQD), South Pacific Subtropical Gyre (SPSG), Indian South subtropical gyre (ISSG), South Atlantic gyre (SATL), Benguela Current Coastal (BENG), Eastern India coast (EAFR), Coastal Chile-Peru Current (CHIL), Southwest Atlantic Shelves (FKLD), and Antarctic (ANTA)

The 270 MAGs included in this study were obtained from 141 metagenomes representing a wide range of oceanic regions and depths (Table S3). Metagenome community profiles, based on SSU rRNA sequence classification, suggest that *Ca*. Poseidoniales (MGII) is the most abundant archaeal order in the majority of these metagenomes with a relative abundance averaging 64 ± 28% (Fig. S7). The highest representation was found in surface waters off the California coast, where the *Ca*. Poseidoniales were the only detectable Archaea in the sample (Fig. S7). These findings are consistent with previous studies in which *Ca*. Poseidoniales were estimated to comprise >50% of archaeal SSU rRNA reads in a subtropical river estuary [39] and >90% of all microscopically observable archaeal cells during a spring bloom [40]. The Tara Oceans expedition is the most extensive marine water survey conducted to date, which included 332 well-documented metagenomic data sets [22]. Leveraging this resource, we mapped reads from the Tara Oceans metagenomes onto a dereplicated set of the 270 MAGs, comprising 204 MAGs with <99.5% average nucleotide identity. A number of genera were ubiquitously distributed throughout the major ocean basins, including I, K1, K2, and L3, which were abundant from the tropics to polar regions (Fig. 2). In contrast, certain other genera were limited in their distribution with high abundances in specific geographic regions, including L1 in the Southwest Atlantic shelves province, L4 in the Northwest Arabian Sea, J3 in the Antarctic Province, and Q2 in the Gulf Stream and Antarctic Province (Fig. 2; S8).

**Fig. 3:**
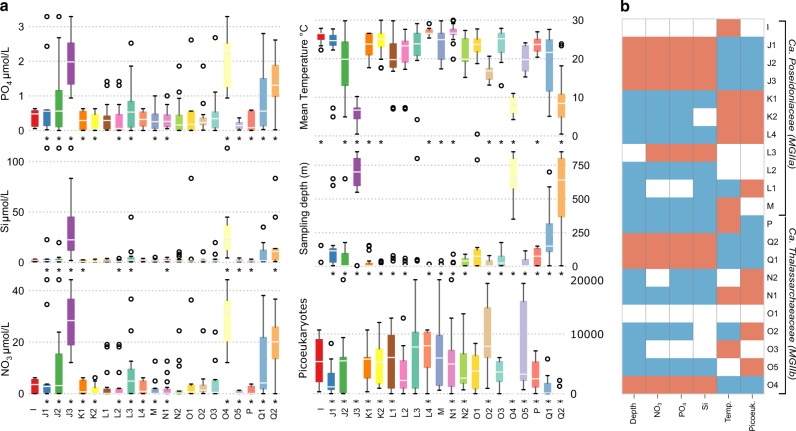
Chemico-physical parameters correlated with *Ca*. Poseidoniales genera abundances. Chemico-physical data were obtained from the TARA Ocean data set. **a** Normalised read abundances of the 90% quantile for each clade, grouped by chemico-physical metadata. Significant correlations between abundance of clades and chemico-physical properties are indicated by an asterisk. **b** Significant correlations between clades and chemo-physical properties. Positive correlations between clades and chemico-physical properties are shown in red, negative correlations in blue. Acronyms used are the following: NO_3_, nitrate; PO_4_, phosphate; Si, Silica (SiO_2_, silicon dioxide), picoeukaryotes, fluorescence-based count of picoeukaryotes (cells/ml); Depth, mean sampling depth (m); Temp, mean temperature °C. Correlations were assessed using a GLM model on a per-clade basis, identifying significance with a Wald test, after adjusting the *p* values with the Benjamini−Hochberg procedure

The majority of *Ca*. Poseidoniales MAGs were recovered from surface water samples (<10 m; Fig. 1, Fig. S9), suggesting that the ancestor of this order may have been a surface dweller. However, a few genera (J1, J2, J3, Q1, and Q2) display deeper distributions in the water column, down to 5000 m, suggesting subsequent adaptation to life below the photic zone. Read mapping of Tara Oceans metagenomic data further supports the partitioning of *Ca*. Poseidoniales genera vertically within the water column, whereby 12 genera were significantly negatively correlated with sampling depth, indicating that they were more abundant in surface waters (Fig. 3). Alternatively, six genera (J1, J2, J3, Q1, Q2, and O4) were significantly positively correlated with sampling depth, and in particular, J3, O4, and Q2 displayed their greatest abundances at depths below 500 m (Fig. 3). These findings were also consistent with nutrient metadata. For example, K1, N1, P, and O5 were all negatively correlated with silica, phosphate, and nitrate concentrations (Fig. 3), in line with their distribution in tropical and subtropical, oligotrophic surface waters (Fig. 2). By contrast, J1, J2, J3, O4, Q1, and Q2 all demonstrated significantly higher abundances in regions with elevated phosphate, and nitrate concentrations (Fig. 3), consistent with their position deeper in the water column. These data suggest different ecological niches for each genus, with a clear separation between those that can be considered oligotrophs versus those found in more copiotrophic environments.

To obtain an indication of temporal dynamics of *Ca*. Poseidoniales, we investigated metagenomes from a surface water time-series. Samples were taken monthly off the coast of south-eastern Australia at Port Hacking over a 15-month period. Overall *Ca*. Poseidoniales represented up to 7% of the total SSU rRNA reads and up to 10% of total reads at a given month (Fig. S10). A number of conspicuous temporal trends were noted in the data. *Ca*. Poseidoniaceae (MGIIa) peaked in the Austral spring, in contrast to *Ca*. Thalassarchaeaceae (MGIIb) which peaked in autumn and winter (Fig. S10). This observation is consistent with previous findings that MGIIa is typically more abundant during spring and summer months, while higher abundances of MGIIb occur during winter, when dissolved inorganic nutrient concentrations increase [5, 41, 42]. At the genus level, the *Ca*. Poseidoniaceae spring peak is largely attributable to L1, L2 and M and the *Ca*. Thalassarchaeaceae autumn and winter peaks are mostly due to genus O2. A generalised additive model was used to identify any specific relationships between the relative abundance of each genus, and physical, chemical, and biological metadata obtained at the Port Hacking sampling site. Significant positive correlations were found between the relative abundances of genera L1, L2, and M, and increased chlorophyll *a* concentrations, dissolved oxygen, and for L1 and L2, blooms of the diatom genera, *Leptocylindrus* and *Thalassiosira* (Fig. S11).

### Core shared metabolism

Consistent with previous analyses of MGII based on smaller data sets, all *Ca*. Poseidoniales genera encode core metabolic functions characteristic of chemoheterotrophs including glycolysis, a TCA cycle, and electron transport chain (Table S4; Fig. S12) indicating aerobic respiration, as posited earlier (for a review see ref. [6]). A proteolytic lifestyle appears to be common to all genera as evidenced by a variety of di- and oligo peptidases, and a high number of genes encoding proteins involved in amino acid degradation pathways (Table S5, S6). Combined with the lack of genes for amino acid biosynthesis, this suggests a dependence on external sources of amino acids which may also serve as sources of carbon and nitrogen. All genera also encode genes for fatty acid degradation (Fig. S13), confirming previous suggestions that MGII archaea might use straight chain fatty acids as growth substrates [9]. None of the 270 *Ca.* Poseidoniales MAGs encodes the enzyme to synthesize the archaeal glycerol-phosphate membrane backbone, but all genera except Q1 encode the enzyme glycerol-3-phosphate (G3P) dehydrogenase required to produce the bacterial/ eukaryotic backbone (Fig. S14; Suppl. Text). The presence of the genes for the bacterial G3P dehydrogenase in combination with a complete set of enzymes for the archaeal lipid biosynthesis pathway (Fig. S14; S15) suggests that Ca. Poseidoniales genera have the potential to synthesize mixed membranes consisting of archaeal type ether lipids with bacterial/eukaryotic G3P glycerol-phosphate backbones. This supports previous findings that MGII archaea have the coding potential for mixed membrane lipids combining archaeal and bacterial features (Villanueva et al. [43]).

**Fig. 4:**
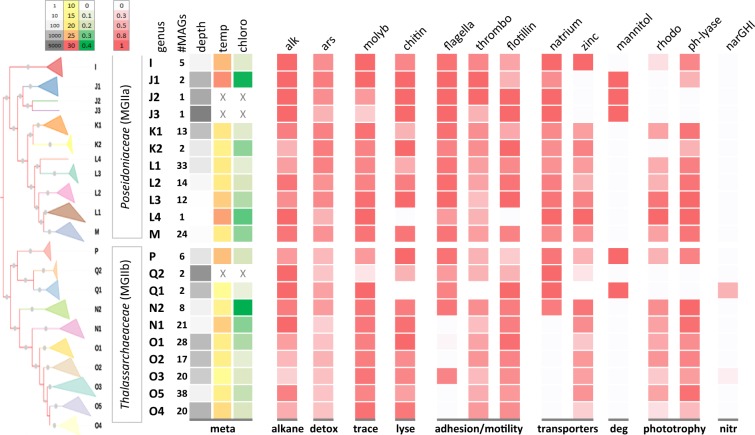
Metabolic potential of *Ca*. Poseidoniales (MGII) genomes. Physico-chemical metadata and inferred metabolic functions uniting and differentiating *Ca*. Poseidoniales families and genera. The encoded functions are based on gene assignments to orthologue groups in the KEGG Orthology (KO) database. Shown are proportions of MAGs in each genus encoding a functional group in the KO database. Gene and gene group names are shown on top and functional categories at the bottom of each heatmap column. #MAGs number of MAGs, depth sample depth in meter, temp sample temperature, chloro sample chlorophyll content in mg Chl/m^3^, meta physico-chemical metadata, alk alkane 1-monooxygenase, alkane alkane degradation, ars arsenate transporter and reductase, detox arsenate detoxification, molyb molybdenum synthesis pathway, trace molybdenum is an essential trace element, chitin chitinase, lyse chitinases lyse algal cells, flagella flagella genes, thrombo thrombospondin genes, flotillin flotillin gene, adhesion/motility functional group of adhesion and motility, natrium natrium transporter, zinc zinc transporter, transporters functional group of ABC-transporters, mannitol mannitol-1-phosphate 5-dehydrogenase, deg mannitol degradation, rhodo proteorhodopsin, ph-lyase deoxyribodipyrimidine photo-lyase, phototrophy functional category phototrophy, (narGHI) nitrate reductase (narGHI) operon, nitr nitrate reduction. Missing data in the metadata columns are indicated by a grey “X”

All *Ca*. Poseidoniales genera except Q2 encode genes for the reduction of toxic arsenate to arsenite and for the transport of arsenite out of the cell (Fig. 3). Such a detoxification system may help *Ca*. Poseidoniales live in oligotrophic waters where low phosphorus concentrations promote the uptake of arsenate, a structural analogue of phosphate, through high affinity phosphate transporters [44] (Suppl. Text). Molybdenum is abundant in the oceans in the form of the molybdate anion [45] and is an essential trace element for several enzymes, including nitrate reductase [46]. Catalytic activity typically requires complexing of molybdenum via cofactors such as the ubiquitous pterin-based molybdenum cofactor (also called molybdopterin-cofactor; Moco) [47]. The biosynthesis of Moco is a complex process and *Ca*. Poseidoniales genomes encode enzymes for all four steps (Fig. 4, Fig. S16).

### Lineage-specific metabolism

A comparison of all genes annotated against the KO database revealed a clear distinction between the metabolic potential of the families *Ca*. Poseidoniaceae (MGIIa) and *Ca*. Thalassarchaeaceae (MGIIb), and more subtle differences between genera (Fig. S17). Key inferred functions separating *Ca*. Poseidoniales families and genera are adhesion, transporters, amino acid and peptide degradation, mannitol metabolism and phototrophy (Fig. 4, Table S5, S6). These are discussed below according to ecological context and their inferred ability to contribute to niche partitioning.

All *Ca*. Poseidoniaceae genera and half of the *Ca*. Thalassarchaeaceae genera possess the archaeal flagella gene operon (Fig. 4, Fig. S18, S19). We posit that the primary function of this operon is for cell adhesion rather than motility, firstly due to a marked absence of identifiable chemotaxis genes and secondly because archaeal flagella are known to contribute to cell-to-cell contact in biofilms and surface attachment [48]. More specifically, it has been suggested that MGIIa could be adapted to a particle-attached lifestyle using flagella [9]. Niche partitioning of *Ca*. Poseidoniales genera by attachment capability also supports previous reports that MGII populations in particulate organic matter (POM) fractions are phylogenetically distinct from free-living MGII [33]. Reanalyzing published size-fractionated metagenomes [33] with genus level resolution revealed that most genera were found in the free-living fraction (0.1–0.8 μm), while others including N2 and L3 occurred in POM fractions only (Fig. S20a). The highest ratio of the flagella gene *flaJ*, in relation to single-copy marker genes, was observed in the large POM fraction (3–20 μm) which also supports the inference that flagella genes are important for particle attachment (Fig. S20b). Further evidence for the importance of cell adhesion in *Ca*. Poseidoniales is the widespread presence of thrombospondin and flotillin genes (Fig. 3), the former of which are a family of secreted glycoproteins implicated in archaeal and bacterial cell adhesion [49] and the latter a protein that triggers changes in cell surface properties [50]. Flotillin was found to be highly transcribed in MGII in coastal upwelling regions, which was speculated to contribute to cell aggregation [10].

**Fig. 5:**
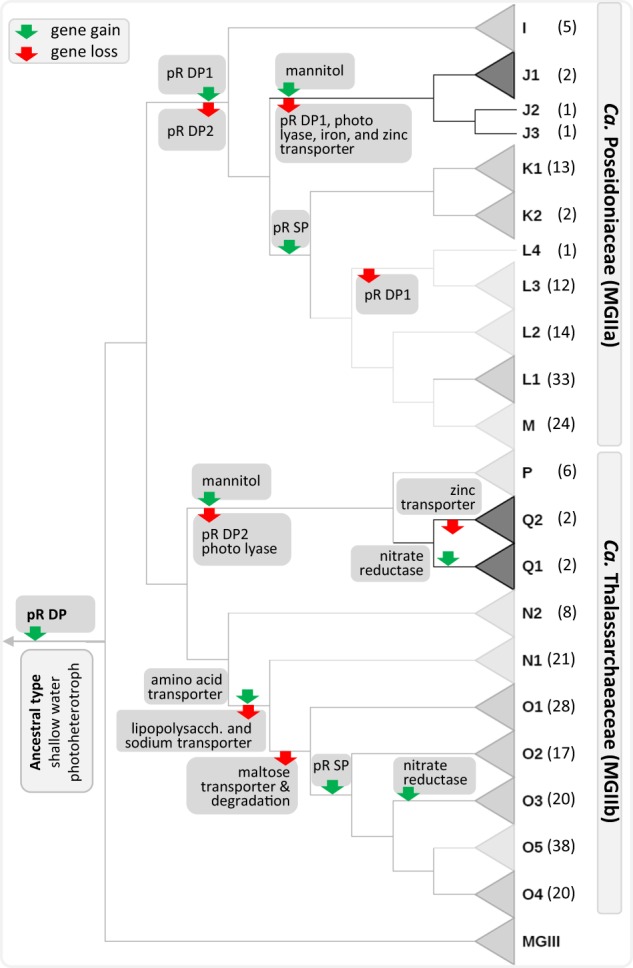
Key metabolic shifts during the evolution of *Ca*. Poseidoniales. Evolutionary relationships among genera are shown via a cladogram with gene acquisitions (green arrows) and gene losses (red arrows). The heterotrophic, shallow water ancestor is show at the root of the tree (left) and its present-day descendants at the leaves (right). The mean sampling depths for each genus is indicated by the grey colour gradient of the clade triangle (light grey = shallowest; dark grey = deepest). pR DP proteorhodopsin deep photic clade, pR DP1 proteorhodopsin deep photic clade 1, pR DP2 proteorhodopsin deep photic clade2, mannitol mannitol synthesis, pR SP proteorhodopsin shallow photic clade

Both *Ca*. Poseidoniales families encode genes involved in amino acid and carbohydrate transport as expected for marine heterotrophic microbes (Fig. S21). Based on the distribution of these traits across the phylogenetic tree, we infer that the ancestor of the N1 and O genera acquired an amino acid transporter and lost their lipopolysaccharide transporter possibly signalling a shift in their ecological niche. Subsequently, most of the group also lost their maltose transporter and associated glucanotransferase, which converts maltose to glucose, suggesting further niche changes from carbohydrate- to amino acid-based metabolism (Fig. 5). A zinc transporter is encoded in most *Ca*. Poseidoniales, but was absent in deep water genera (Fig. 4, Fig. S21). The presence of zinc uptake mechanisms is likely an adaptation to oligotrophic surface waters where low concentrations of free Zn^2+^ are known to limit the growth of phytoplankton [51]. Zinc concentrations are markedly higher in deep compared to surface waters [52].

The class *Ca*. Poseidoniia is unusual among the Archaea, along with the Haloarchaea, as one of the few groups in this domain that encode rhodopsins [9]. More specifically, *Ca*. Poseidoniia encode proteorhodopsin (pR) which is a retinal-containing integral membrane protein that can act as a light-driven ion pump or as a photosensor to monitor environmental light signals [53, 54]. The gene encoding this protein, *pR* has been detected previously in MGII genomic fragments and MAGs recovered from surface waters (<150 m) [7, 9, 10] but not from deep-sea MGII fosmids and transcriptomic assemblies [8, 55]. A total of 153 of the 270 MAGs in the present study representing all genera except J1–3, Q1–2, and K2 encode proteorhodopsin (Fig. 4, Fig. S22). The percentage of MAGs carrying the *pR* genedecreased with sampling depth, which corresponds with declining light penetration in open ocean waters (Fig. S23a). It is possible that missing *pR* genes could be attributed to incomplete MAGs; however, none of the genera recovered exclusively from deeper sites down to 2100 m (J1, J2, J3, Q1, Q2; Fig. S23a), nor individual MAGs recovered below 200 m encoded pR (Fig. S22) consistent with previous observations that deeper MGII lack this gene [8, 55]. With the exception of K2, all shallow water genera (<10 m) have representatives encoding *pR* genes. We thus hypothesise that the loss of proteorhodopsin in deep water genera is a consequence of life below the photic zone.

Of the proteorhodopsin-containing genera, the *Ca*. Poseidoniia *pR* genes fall into two phylogenetically distinct clades within a larger radiation of bacterial homologues (Fig. S24; S25), which we and others attribute to ancient interdomain horizontal gene transfer (HGT) events [7, 56]. We also infer that each pR clade is characterised by a specific absorption maximum attributed to a single amino acid residue at position 315 in our alignment (Fig. S26). The nonpolar methionine residue (Met, M) results in maximal light absorption at 525 nm (green light) and the polar glutamine residue (Gln, Q) at 490 nm (blue light) [54]. Adaptations of pR to different wavelengths (*i.e.* spectral tuning) are known for marine bacteria including the SAR86 clade which absorb light maximally at 527 nm (green light) in surface waters, but at 490 nm (blue light) in deeper (~75 m) waters. This ensures more efficient use of light energy as blue light penetrates deeper than green light in the open ocean [57]. A similar pattern is observed in *Ca*. Poseidoniales with a higher percentage of inferred blue light absorbing pR in MAGs recovered from deeper waters (50–200 m; Fig. S23b). Therefore, we refer to the pR clade with the blue light signature as DP for deep photic (indicating adaptation to deeper photic waters) and the clade with the green light signature as SP for shallow photic (indicating adaptation to shallow photic waters; Fig. S24; S25). Both clades also show well-conserved proton donor positions at pR amino acid residue 318 (Fig. S26); clade DP encodes glutamic acid (Glu, E) at this position and clade SP encodes lysine (Lys, K). While Glu318 pRs have been experimentally verified as proton pumps [53], Lys318 is less well understood, but has been identified as the proton donor for a pR proton pump in a permafrost bacterium [58]. Proteorhodopsins used as photosensors have been reported to have either a phenylalanine or serine in position 318 [58], suggesting that *Ca*. Poseidoniales use their proteorhodopsins as light-driven proton pumps to enhance growth or promote survival during starvation.

**Fig. 6:**
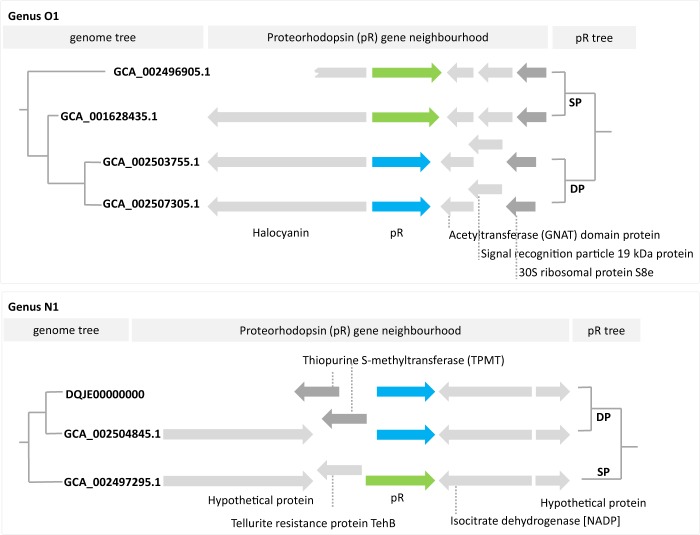
Proteorhodopsin (pR) gene neighbourhoods. Shown are two examples of the *pR* gene neighbourhoods, of closely related MAGs within a genus, with recent horizontal gene transfer events. In both cases, genus O1 (upper panel) and genus N1 (lower panel), we infer that the recently transferred *pR* gene has replaced the previous *pR* gene

The pR phylogeny is strongly at odds with the inferred organismal phylogeny, which indicates extensive and ongoing HGT of *pR* genes within the *Ca*. Poseidoniia, including both ancient (between domains) and recent (within genera) events (Fig. S24; S25; Fig. S27). However, based on the overall distribution of pRs, we predict that the ancestor of the *Ca*. Poseidoniia contained a proteorhodopsin and was therefore a surface dweller. Consistent with this inference is the loss of *pR* genes in several *Ca*. Poseidoniales genera found below the photic zone including J1, J2, J3, N2, P, Q1, and Q2 (Fig. S27). Strikingly, only one *pR* gene homologue could be verified in each proteorhodopsin-encoding MAG, despite the potential to acquire additional copies through HGT. The conservation of pR gene neighbourhoods among closely related MAGs (Fig. 6), regardless of the type of *pR* gene (SP or DP), suggests a possible mechanism for maintaining pR in single copy; horizontally acquired pR genes replace the existing copy by inserting into the same location on the genome possibly via homologous recombination or mediated via transposable elements [59, 60]. This mechanism would also allow the archaeal host to quickly transition to a new ecological niche (i.e. depth) based on pR spectral tuning if the replacement involved a change from an SP to DP type or *vice versa* (Fig. 6; Fig. S27). All genera possessing* pR* genes also encode a photolyase (phrB; EC:4.1.99.3), an enzyme which repairs UV-damaged DNA by using light of the near-UV/blue light region. Like the *pR* gene, the evolutionary history of the photolyase gene is a patchwork of ancient and recent HGT events supporting the inferred ability of *Ca*. Poseidoniales to transition to new environmental niches (Fig. 5).

*Ca*. Poseidoniales may also play a role in the marine nitrogen cycle due to their encoded capacity for nitrate uptake and respiration. In particular, two MAGs from genus O3 and one from genus Q1 include the complete operon encoding a membrane-bound nitrate reductase, NarGHI (Fig. 4; Fig. S28) capable of reducing nitrate to nitrite. These MAGs also possess the *narJ* gene, which encodes the chaperone required for the assembly of the nitrate reductase complex, and encode one or multiple NarK/NasA family nitrate transporters which can facilitate nitrate transport through the cell wall into the cytoplasm [61]. The NarGHI nitrate reductase allows nitrate to be used as the final electron acceptor and this ability enhances bacterial survival by nitrate respiration under anaerobic conditions [61]. Therefore, the encoded respiration of nitrate could be an adaptation to low oxygen tension and indeed all three MAGs were recovered from low oxygen zones or from deep water samples with low oxygen availability, respectively (Suppl. Text). The sporadic presence of *nar* genes in only three *Ca*. Poseidoniales MAGs is likely due to recent horizontal transfer events (Fig. 3). We infer that the nitrate reductase genes were acquired from Proteobacteria and the nitrate transporter genes from other Archaea and/or Bacteria (Fig. S28, S29). Previous studies have also suggested recent HGT of *nar* genes between phylogenetically remote microbial species [62, 63].

## Conclusion

The recent availability of hundreds of near complete draft genomes belonging to the uncultured archaeal lineage, *Ca*. Poseidoniales (*ord. nov*), formerly known as marine group II (MGII), has made it possible to conduct a high resolution phylogenomic analysis. The picture that emerges is one of a surface ocean-dwelling photoheterotrophic ancestor that evolved to occupy multiple related ecological niches based in part on spectral tuning of proteorhodopsin genes, and in some instances loss of phototrophy altogether to occupy deeper aphotic waters. Genes encoding proteorhodopsins tuned to blue or green light have been horizontally passed between members of the *Ca*. Poseidoniales over the entire history of the lineage using an overwrite mechanism that ensures only one copy of the gene is maintained at any one time. We infer that this has enabled abrupt changes in light absorption capacity of some members of the group, presumably in response to changing ecological conditions. Genera that have entirely lost their phototrophic capability have associated changes consistent with a move to deeper aphotic nutrient-rich waters including loss of UV repair and transporter genes. Our analysis and the availability of nearly 300 draft genomes provide important new insights into the likely ecology and trophic strategies of a universally abundant, but poorly understood marine archaeal lineage.

## Electronic supplementary material

Supplementary Text

Supplementary Tables and Figures
