# Supplementary material for: A phylogenomic and ecological analysis of the globally abundant Marine Group II archaea (Ca. Poseidoniales ord. nov.)
Source: ISME J. 2018 Oct 15;13(3):663–75. doi: 10.1038/s41396-018-0282-y (PMC6461757; doi:10.1038/s41396-018-0282-y)
Supplement: Supplementary file 1 — Supplementary Text [file 41396_2018_282_MOESM1_ESM.docx]

**Supplementary text**

**Proposal of type material and higher ranks**

We propose two type species based on two of the 270 MAGs used in the present study; *Candidatus* Poseidonia alphae (GCA_002505405.1) representing subgroup IIa and *Candidatus* Thalassarchaeum betae (PSPG00000000) representing subgroup IIb. Based on these type species, we further propose the families *Candidatus* Poseidoniaceae fam. nov. and *Candidatus* Thalassarchaeaceae fam. nov., the order *Candidatus* Poseidoniales ord. nov. and the class *Candidatus* Poseidoniia class nov.

**Description of *Candidatus* Poseidonia gen. nov.**

*Candidatus* Poseidonia (Po.se.i.do’ni.a. N.L. fem. n. *Poseidonia* named after Poseidon, the Greek god of the sea). Inferred to be of marine origin, a motile, aerobic, photoheterotroph, and able to ferment a wide range of carbohydrates with a focus on peptides. Highest abundances were recorded for surface waters (0-10m). Formerly identified as **clade M** [1]. Type species: *Candidatus* Poseidonia alphae.

**Description of *Candidatus* Poseidonia alphae**

*Candidatus* Poseidonia alphae *(N.L. gen. n. alphae* of alpha, the first letter of Greek alphabet), to honour the original description of this group as II-∝ [2]. Description is the same as for *Candidatus* Poseidonia gen. nov. Represented by the MAG (metagenome-assembled genome) GCA_002505405.1 (1.94 Mbp; **Fig. S6**, **S30**), obtained from metagenomic sequences from surface waters (1m) at Kabeltonne, Helgoland, in the North Sea (54.174 N 7.900 E). Based on the genome reporting standards for MAGs [3], the estimated completeness 90.9%, contamination 0%, and the presence of the 5S (108bp), 16S (1453bp), and 23S (2838bp) rRNA gene and 41 tRNAs *Candidatus* Poseidonia alphae is defined as “high-quality” draft MAG.

**Description of *Candidatus* Poseidoniaceae fam. nov.**

*Candidatus* Poseidoniaceae (Po.se.i.do.ni.a’ce.ae. N.L. fem. n. *Poseidonia*, type genus of the family; N.L. fem. pl. n. *Poseidoniaceae*, the *Poseidonia* family). The description is the same as for *Candidatus* Poseidonia gen. nov. with the following additions. *Candidatus* Poseidoniaceae fam. nov. was previously known as and **“Group II-∝”** and **“Marine Group IIa”** [2, 4]. Eleven genus-level clades I, J1, J2, J3, K1, K2, L1, L2, L3, L4, and *Candidatus* Poseidonia (M) representing the rank of genera were assigned to this family. All members of the family were inferred to be marine, motile heterotrophs, encoding phototrophic and non-phototrophic lifestyles with a distribution from surface waters to the deep sea (1m-500m). Type genus: *Candidatus* Poseidonia gen. nov.

**Description of *Candidatus* Thalassarchaeum gen. nov.**

*Candidatus* Thalassarchaeum (Tha.lass.ar.chae’um. Gr. fem. n. *thalassa* the sea; also the primeval spirit of the sea; N.L. neut. n. *archaeum* (from Gr. adj. *archaios* -*ê* -*on*, ancient) archaeon; N.L. neut. n. *Thalassarchaeum* the archaeon from the sea). Inferred to be of marine origin, representing non-motile, aerobic heterotrophs containing phototrophic and non-phototrophic members. Formerly identified as **clade O** [1] and **genus O4** (this study). This genus name is proposed for continuity with the orthographically incorrect “*Thalassoarchaea”* suggested by [5] for low GC MGIIb fosmid-genomes. Type species: *Candidatus* Thalassarchaeum betae.

**Description of *Candidatus* Thalassarchaeum betae**

*Candidatus* Thalassarchaeum betae (N.L. gen. n. *betae* of beta, the second letter for the Greek alphabet), named to honour the original description of this group as II-β [2]. Description is the same as for *Candidatus* Thalassarchaeum gen. nov. Represented by the MAG (metagenome-assembled genome) PSPG00000000 (1.48 Mbp; **Fig. S6, S30**), obtained from metagenomic sequences retrieved from water deep sea water sample (1953 to 4946m) from the Mid-Cayman Rise, in western Caribbean Sea (18.32 N, 81.43 W) and first reported as “Cayman51_deep” [6]. Based on the genome reporting standards for MAGs [3], the estimated completeness 92.2%, contamination 0%, the presence of the 5S (111bp), 16S (1275bp), and 23S (2555bp) rRNA gene and 37 tRNAs encoding 19 amino acids, *Candidatus* Thalassarchaeum betae is defined as “high-quality” draft MAG.

**Description of *Candidatus* Thalassarchaeaceae fam. nov.**

*Candidatus* Thalassarchaeaceae (Tha.lass.arch.ae.a’ce.ae. N.L. fem. pl. n. *Thalassarchaeaceae* the *Candidatus* Thalassarchaeum family). The description is the same as for *Candidatus* Thalassarchaeum gen. nov. with the following additions; *Candidatus* Thalassarchaeaceae fam. nov. was previously designated “**Group II-β”** and “**Marine Group IIb**” [2, 4]. Ten genus-level clades N1, N2, O1, O2, O3, *Candidatus* Thalassarchaeum (O4), O5, P, Q1, and Q2 were assigned to this family. Inferred metabolic features suggest motile and non-motile heterotrophic family members, some of which are phototrophic, others acquired the genes for anaerobic nitrate respiration via horizontal gene transfer. Type genus: *Candidatus* Thalassarchaeum gen. nov.

**Description of** ***Candidatus* Poseidoniales ord. nov.**

*Candidatus* Poseidoniales (Po.se.i.do.ni.a’les. N.L. fem. n. *Poseidonia* the type genus of the order; N.L. fem. pl. n. *Poseidoniales* the *Poseidonia* order.). The description is the same as for *Candidatus* Poseidonia gen. nov. with the following additions; -ales ending to denote a order. *Candidatus* Poseidoniales ord. nov. was previously known as “Marine Group II” and comprises two families *Candidatus* Poseidoniaceae fam. nov. and *Candidatus* Thalassarchaeaceae fam. nov.

**Description of *Candidatus* Poseidoniia class. nov.**

*Candidatus* Poseidoniia (Po.se.i.do’ni.ia. N.L. fem. n. *Poseidonia* the type genus of the type order of the class; N.L. neut. pl. n. *Poseidoniia* the *Poseidonia* class). The description is the same as for *Candidatus* Poseidonia gen. nov. with the following additions; *Candidatus* Poseidoniia class. nov. comprises the type order *Candidatus* Poseidoniales ord. nov. and order Marine group III (MGIII).

**Core shared metabolism**

**Fatty acids**

All *Ca*. Poseidoniales genera encode genes for fatty acid degradation (**Fig. S13**), confirming previous suggestions that MGII archaea might utilise straight fatty acid chains as growth substrates [7]. Hydrocarbons such as alkanes are abundant in the marine environment, mostly produced by cyanobacteria and algae [8]. The presence of the *alkB1_2* genes in all *Ca*. Poseidoniales genera (**Fig. 3; Fig. S13**) is remarkable, since alkane degradation seems to be uncommon among Archaea [9], indicating that this order may play a role in marine alkane recycling.

**Membrane lipids**

The composition of membrane lipids is considered a hallmark feature differentiating the domains of life. Archaeal lipids are characterized by branched hydrocarbon chains (isoprene-based alkyl chains) attached to the glycerol phosphate-backbone via ether linkages. In contrast, Bacteria and Eukaryotes feature unbranched fatty acid chains attached to the glycerol-phosphate backbone via ester linkages. These traits are typical but not unique since fatty acids have been found in some Archaea and membrane spanning ether-lipids lipids have been reported in some Bacteria [10].

The remaining domain exclusive membrane feature is the stereochemistry of the glycerol-phosphate backbone. Archaeal membrane backbones are made of sn-glycerol-1-phosphate (G1P) whereas Bacteria and Eukaryote backbones consist of the sn-glycerol-3-phosphate (G3P). The enzyme synthesizing the G1P and G3P backbones (glycerol-1- and glycerol-3-phosphate dehydrogenase) belong to different protein superfamilies and lack any structural or sequence homologies [10]. This ‘lipid divide’ between domains presents a problem for theories explaining eukaryogenesis, since most models require a transformation of the host organism’s membrane from an archaeal G1P membrane to a bacterial/eukaryotic G3P membrane.

Our results confirmed previous studies concluding that marine group II Archaea lack the gene to synthesize G1P, but instead encode G3P synthesis [7, 11]. In fact, all *Ca.* Poseidoniales genera bear the gene *gpsA* (K00057), which encodes glycerol-3-phosphate dehydrogenase [EC:1.1.1.94], whilst lacking *egsA* (K00096), the gene for glycerol-1-phosphate dehydrogenase [EC:1.1.1.261] (**Fig. S14**). The lack of *egsA* indicates, that none of the *Ca*. Poseidoniales genera have the ability to produce archaeal G1P lipids, despite the presence of all genes encoding enzymes for every other step in the archaeal lipid biosynthesis pathway (**Fig. S14**). A detailed phylogenetic analysis of the key enzyme in the remaining archaeal lipid biosynthesis, GGR (digeranylgeranyl-glycerophospholipid reductase), confirms that this protein is of archaeal origin, although it has been subjected to a number of ancient horizontal transfers including into bacterial recipients (**Fig S15**).

Interestingly glycerol-3-phosphate dehydrogenase (gene *gpsA*; K00057) was not encoded in any of seven investigated MGIII MAGs, on the contrary the majority of MGIII MAGs beared the gene *egsA* (K00096) for glycerol-1-phosphate dehydrogenase (*Rinke pers. comm.)* This suggests that several archaeal lineages, including *Ca.* Poseidoniales (MGII) and Lokiarchaeota [11], but excluding MGIII, are potentially able to synthesize mixed membranes consisting of archaeal type ether lipids with bacterial/eukaryotic G3P glycerol backbones.

In addition, our results confirmed that most *Ca.* Poseidoniales genera carry homologs of the *plsC* gene encoding 1-acyl-sn-glycerol-3-phosphate acyltransferase. This enzyme converts lysophosphatidic acid (LPA) into phosphatidic acid and hints a putative bacteria-like fatty acid synthesis pathway in *Ca.* Poseidoniales (**Fig. S14**).

**Chitinases**Glucosidases, chitinases, cellulases and other enzymes that help degrade plant cell walls are also involved in the utilisation of algal cells. Thereby bacterial degradation is mediated by extracellular enzymes [12]. This includes bacterial strains producing carbohydrases for degradation of polysaccharides present in the algal cell wall, and showing carbohydrase activity with glucans, galactans, galactomannans and pectin [13]. The authors of this study concluded that this carbohydrate utilisation suggests the potential use of cast-off algal cell wall products. However, microbes are also known to actively lyse algal cells. Two bacterial genera were identified as the source for the cell lysis of the red-tide forming algae *Alexandrium tamarense* by utilizing extracellular enzymes including β-Glucosidase, aminopeptidase and chitinase [14]. Chitinase producing bacteria are also lethal for diatoms since microbes use this enzyme to degrade algal cell walls, which eventually leads to algal lysis and death [15]. In addition, chitin is also a major component of cuticles and exoskeletons of zooplankton [16]. Overall, chitin is the most abundant natural polysaccharide in the marine environment [17] and many bacteria use chitin as the sole carbon and nitrogen source [18]. We found that all genera, except L4 and Q2, in *Ca*. Poseidoniaceae (MGIIa) and *Ca*. Thalassarchaeaceae (IIb) encode chitinase (K01183; EC:3.2.1.14). Within the domain Archaea chitinases are known from several Euryarchaeota and a Crenarchaeum [19, 20], and Archaea were among the highest contributors to the chitin-active community during induced phytoplankton blooms in the southern Ocean [21]. *Ca*. Poseidoniales genera could use chitin as a carbon and nitrogen source, and could be important archaeal players in the global chitin bioconversion in surface waters. The latter argument is supported by the widespread occurrence of chitinase genes in *Ca*. Poseidoniales genomes and by a previous study reporting that chitinases were abundant in the MG-II transcriptomic data [6].

**Lineage specific metabolism**

**Mannitol**Several *Posedoniales* genera encode mannitol-1-phosphate 5-dehydrogenase (**Fig. 4; Fig. S31**), which is the key enzyme in mannitol synthesis and degradation, a function not previously reported in archaea. In the marine environment, mannitol production is widespread among algae [22] and mannitol represents up to 25% of the dry weight of some species [23]. Therefore the *mtlD* gene homologues in *Posedoniales* could indicate that Archaea play a role in marine mannitol degradation, possibly recycling this sugar alcohol from algal sources, further highlighting likely close ecological ties between the *Posedoniales* and phytoplankton. However, we found no genes encoding the reaction from mannitol to mannitol-phosphate, suggesting that *Posedoniales* may acquire mannitol as mannitol-phosphate (**Fig. S31)**.

**Arsenate detoxification**

Arsenate has a structural analogy to inorganic phosphate and can be introduced into the cell through phosphate transporters [24] disrupting metabolic reactions requiring phosphorylation, including adenosine triphosphate (ATP) synthesis. Arsenate resistance or detoxification becomes especially critical in oligotrophic waters where phosphate concentrations are below those of Arsenate [25]. Such a detoxification is performed by arsenate reductases (ArsCS), which are small cytoplasmic redox enzymes that evolved independently to reduce the toxic arsenate to arsenite (As(III)) and have been categorised into distinct ArsC classes [26]. Most *Ca*. Poseidoniales clades possess homologs of an arsenite efflux pump (*arsB*, K03893), and for an arsenate reductase (*arsC1*, K00537; *arsC2*, K03741), and an arsenite-stimulated ATPase (*arsA*, K01551) which increase the efficiency of arsenite efflux through *arsB* [25]. While most *Ca*. Poseidoniaceae (MGIIa) genera possess two arsenate reductase genes (*arsC1, arsC2*), *Ca*. Thalassarchaeaceae (IIb) genera contain only one copy (*arsC2*), except for clade N1. In the deep sea bacterium *Geobacillus kaustophilus* the expression of both arsC genes was strongly induced in the presence of arsenate, indicating that both encode a functional arsenate reductase [27].

**Proteorhodopsin.**

Proteorhodopsin (pR) is a retinal-containing integral membrane protein that can act as a light-driven proton pump or as a photo sensor to monitor environmental light signals [28]. The name proteorhodopsin refers to the initial identification of the gene in the group in γ-proteobacteria [29]. The gene encoding this protein has been detected previously in *Ca*. Poseidoniales genomic fragments and population bins recovered from surface waters [5, 7, 30] but not from deep sea MGII fosmids and transcriptomic assemblies [31, 32]. A total of 153 of the 270 MAGs in the present study representing all genera except J1- 3, Q1-2, and K2 encode proteorhodopsin. As previously noted [7], the MGII proteorhodopsin genes form two phylogenetically distinct clades (DP and SP) within a broader radiation of bacterial homologs, which we interpret to indicate two ancient lateral transfers from bacterial donors into *Ca*. Poseidoniales. Clade DP shares a common ancestor with *Proteobacteria* whereas clade SP is only distantly related to all other bacterial *pR* genes. Each MAG, except for three (DQKI00000000/ U_67557, GCA_002504805.1/ U_67237), GCA_002495385.1/ U_69271), maintained only a single gene copy which is consistent with previous studies assuming microorganism carry only on *pR* gene copy [33, 34]. The contigs of the three MAGs containing a second *pR* copy where likely a result of binning errors, since the second copy aligned with a different genus or order. Surprisingly the phylogenetic split of pR into these two clades did not follow the genome nor the 16S phylogeny since both clades consist of a mix of MAGS from *Ca*. Poseidoniaceae (MGIIa) and *Ca*. Thalassarchaeaceae (MGIIb) genera. A phylogeny of a single gene conflicting with genome phylogeny usually points to a rampant horizontal gene transfer (HGT) and indeed HGT of *pR* genes was found to be widespread and was hypothesized to have involved transfers between Bacteria and Archaea [30, 35]. Such HGTs within *Ca*. Poseidoniales must have been recent evolutionary events since closely related MAGs harbor *pR* genes from clade DP or clade SP respectively. Even more intriguing is the fact that each MAG contains only a single *pR* copy (see above). A most likely explanation for a single copy gene with a phylogeny decoupled from the host phylogeny is that a plasmid or virus is carrying that gene. Indeed, proteorhodopsin genes have been identified in giant **viruses** and such viral homologs were found to be abundant in marine environments [36]. Although the lack of conservation of the proton donor carboxylate (located in Helix C) indicated that these viral proteorhodopsin homologs were sensory rhodopsins rather than light-dependent proton pump (see below), viruses carrying the proton pump version might still await discovery.

**Function.** A main difference observed between transport and sensory rhodopsins is the amino acid residue at the proton donor position. If the proton donor group is aspartic acid or glutamic acid, the rhodopsin may serve as an electron pump [28], but  a lack of conservation of the proton donor carboxylate indicates that the homologs serve a sensory function [36]. Among *Ca*. Poseidoniales the proton donor position is well conserved (pos 318); clade DP has the residue glutamic acid (glu; code E ) and clade SP has lysine (lys; code K). While glutamic acid is characteristic for proton pumps [28] a lysine residue has been reported previously for a MGII MAG [7] and has been identified as the proton donor for the proteorhodopsin proton pump in a permafrost bacterium  [37]. This suggests that MGII encodes a proteorhodopsin that functions as a light-driven proton pump and could enhance growth or promote survival during starvation.

**Ecology.** The two different rhodopsin clades present in *Ca*. Poseidoniales are further characterized by specific absorption maxima. This spectral tuning is attributed to a single amino acid residue at position 315 in our alignment. The non-polar methionine residue (Met, M) results in a green (525nm) and the polar glutamine residue (Gln, Q) in a blue (490nm) absorption [38]. Adaptations to different wavelength are known for marine bacteria. For example, SAR86 bacteria absorbed light at a 527nm (green light) maximum in surface waters, but at a 490nm (blue light) maximum at 75m indicating an adaptation to ensure maximum absorption of blue light with penetrates deeper in the open ocean [39]. We found a similar pattern for *Ca*. Poseidoniales spectral tuning with a higher presence of *bop* genes encoding blue absorption wavelength in MAGs recovered from deeper waters.

Investigating the gene neighbourhood of the proteorhodopsin genes in *Ca*. Poseidoniales, we found that the most frequent genes directly up- and down-stream (∓1) were annotated (PROKKA annotation) as hypothetical proteins, acyltransferase family proteins, putative ABC transporter ATP-binding proteins, and cupin domain proteins. The putative ABC transporter ATP-binding proteins are likely part of an ABC transporter complex and responsible for energy coupling to the transport system. The cupin domain proteins represent a functionally very diverse superfamily of proteins named after its conserved barrel domain (cupa) and their functional roles in regards to rhodopsin genes need further investigation. The presence of acyltransferase family proteins as direct neighbours to proteorhodopsin could indicate that *Ca*. Poseidoniales encode the lecithin-retinol acyltransferase (LRAT), an enzyme present mainly in the retinal pigmented epithelial cell, which is part of a two-cell recycling system that replenishes the 11-cis-retinal chromophore of rhodopsin [40]. Interestingly, in seven *Ca*. Thalassarchaeaceae MAGs the proteorhodopsin gene neighbourhood included a gene encoding the tellurite resistance protein TehB. Tellurite is extremely harmful to most microorganisms, and its toxicity has been associated with the establishment of an oxidative stress status, including ROS generation [41, 42]. Tellurite resistant marine microbes have been reported from deep-ocean hydrothermal-vent environments that are rich in heavy metals and metalloids [43], however the benefits of harboring TehB for *Ca*. Poseidoniales, which occur in the open Ocean, are unknown.

**Nitrate***Ca*. Poseidonales might also play a role in the marine nitrogen cycle due to their encoded capacity for nitrate uptake and nitrate respiration. In particular, two MAGs from the genus O3 and one from genus Q include the complete operon encoding a membrane‐bound nitrate reductase (NarGHI) capable of reducing nitrate to nitrite. Membrane‐bound nitrate reductases are constituted of three subunits (NarG, NarH, and NarI) and are responsible for generating the proton motive force across the cytoplasmatic membrane of the cell [44]. The three *Ca*. Poseidoniales MAGs encode all three subunits and possess the *narJ* gene, which encodes the chaperone required for proper molybdenum cofactor insertion and final assembly of the nitrate reductase complex (**Fig. S28).** In the gene neighbourhood of the *narGHI* operon the MAGs encode one or two NarK/NasA family nitrate transporter, and it has been shown for *E.coli* that nitrate transverses the cell wall into the cytoplasm via Nar transmembrane proteins, in particular NarK2 which is a nitrate/H+ symporter [45]. The *narGHI* operon exists in many bacteria playing a key role in the nitrogen cycle and the encoded nitrate reductase allows microbes to use nitrate as final electron acceptor. This ability has been shown to enhance bacterial survival by nitrate respiration under anaerobic conditions [45]. Among bacteria which can use nitrate as a terminal electron acceptor instead of oxygen, to preserve the proton gradient for energy production in anaerobic conditions, are *M. tuberculosis* and *Escherichia coli* [46, 47]. When grown anaerobically with nitrate as a respiratory oxidant, *E. coli* develops a respiratory chain terminated by a membrane-bound nitrate oxidoreductase (NarGHI) [46]. Therefore, the encoded respiration of nitrate in *Ca*. Poseidoniales could be an adaptation to low oxygen tension since all 3 MAGs were recovered from low oxygen zones or from deep water samples with low oxygen availability, respectively. The two O3 MAGs were recovered from a shallow water low oxygen zone off the coast of Mexico (GCA_002505385.1/ U_69541; 80m) and from a depth of 174-180 m, targeting a marine oxygen minimum zone (U_77941/ GCA_002497545.1; 177m). The genus Q genome originates from a sea deep sample (DQJW00000000/ U_68079; 600m) which, based on the fact that Oxygen decreases and Nitrate increase with depth [48], e.g. the nitrate maximum in the Pacific is at about 1000m, was most likely microaerophilic or even anaerobic and rich in nitrate. The sporadic presence of *nar* genes in only three Poseidonales MAGs could be explained by horizontal gene transfer (HGT). There is evidence that *nar* genes were acquired by hyperthermophilic archaea and some bacteria via HGT [49] and it has been suggested that HGT of *nar* operons exists in archaeal and epsilonproteobacterial species [50]. In the case of the *Ca*. Poseidoniales MAGs the *nar* operon has been acquired from Proteobacteria and the nak/nasA genes from other archaea and/or bacteria. In summary, our results suggest that *Ca*. Poseidoniales are able to influence the availability of reactive nitrogen species in marine environments, an ability that so far has been only attributed to *Thaumarchaeota* (MGI Archaea) environments [51].

**References**

1. Galand PE, Gutiérrez-Provecho C, Massana R, Gasol JM, Casamayor EO. Inter-annual recurrence of archaeal assemblages in the coastal NW Mediterranean Sea (Blanes Bay Microbial Observatory). *Limnol Oceanogr* 2010; **55**: 2117–2125.

2. Massana R, DeLong EF, Pedrós-Alió C. A Few Cosmopolitan Phylotypes Dominate Planktonic Archaeal Assemblages in Widely Different Oceanic Provinces. *Appl Environ Microbiol* 2000; **66**: 1777–1787.

3. Bowers RM, Kyrpides NC, Stepanauskas R, Harmon-Smith M, Doud D, Reddy TBK, et al. Minimum information about a single amplified genome (MISAG) and a metagenome-assembled genome (MIMAG) of bacteria and archaea. *Nat Biotech* 2017; **35**: 725–731.

4. Zhang CL, Xie W, Martin-Cuadrado A-B, Rodriguez-Valera F. Marine Group II Archaea, potentially important players in the global ocean carbon cycle. *Front Microbiol* 2015; **6**.

5. Martin-Cuadrado A-B, Garcia-Heredia I, Moltó AG, López-Úbeda R, Kimes N, López-García P, et al. A new class of marine Euryarchaeota group II from the mediterranean deep chlorophyll maximum. *ISME J* 2015; **9**: 1619–1634.

6. Li M, Baker BJ, Anantharaman K, Jain S, Breier JA, Dick GJ. Genomic and transcriptomic evidence for scavenging of diverse organic compounds by widespread deep-sea archaea. *Nat Commun* 2015; **6**: 8933.

7. Iverson V, Morris RM, Frazar CD, Berthiaume CT, Morales RL, Armbrust EV. Untangling Genomes from Metagenomes: Revealing an Uncultured Class of Marine Euryarchaeota. *Science* 2012; **335**: 587–590.

8. Lea-Smith DJ, Biller SJ, Davey MP, Cotton CAR, Perez Sepulveda BM, Turchyn AV, et al. Contribution of cyanobacterial alkane production to the ocean hydrocarbon cycle. *Proc Natl Acad Sci U S A* 2015; **112**: 13591–13596.

9. Nie Y, Chi C-Q, Fang H, Liang J-L, Lu S-L, Lai G-L, et al. Diverse alkane hydroxylase genes in microorganisms and environments. *Scientific Reports* 2014; **4**: 4968.

10. Caforio A, Driessen AJM. Archaeal phospholipids: Structural properties and biosynthesis. *Biochimica et Biophysica Acta (BBA) - Molecular and Cell Biology of Lipids* 2017; **1862**: 1325–1339.

11. Villanueva L, Schouten S, Damsté JSS. Phylogenomic analysis of lipid biosynthetic genes of Archaea shed light on the ‘lipid divide’. *Environmental Microbiology* 2017; **19**: 54–69.

12. Afi L, Metzger P, Largeau C, Connan J, Berkaloff C, Rousseau B. Bacterial degradation of green microalgae: incubation of Chlorella emersonii and Chlorella vulgaris with Pseudomonas oleovorans and Flavobacterium aquatile. *Organic Geochemistry* 1996; **25**: 117–130.

13. Arora M, Anil AC, Delany J, Rajarajan N, Emami K, Mesbahi E. Carbohydrate-degrading bacteria closely associated with Tetraselmis indica: influence on algal growth. *Aquatic Biology* 2012; **15**: 61–71.

14. Wang X, Li Z, Su J, Tian Y, Ning X, Hong H, et al. Lysis of a red-tide causing alga, Alexandrium tamarense, caused by bacteria from its phycosphere. *Biological Control* 2010; **52**: 123–130.

15. Li Y, Lei X, Zhu H, Zhang H, Guan C, Chen Z, et al. Chitinase producing bacteria with direct algicidal activity on marine diatoms. *Scientific Reports* 2016; **6**: 21984.

16. Tang KW, Turk V, Grossart H-P. Linkage between crustacean zooplankton and aquatic bacteria. *Aquatic Microbial Ecology* 2010; **61**: 261–277.

17. Tharanathan RN, Kittur FS. Chitin — The Undisputed Biomolecule of Great Potential. *Critical Reviews in Food Science and Nutrition* 2003; **43**: 61–87.

18. Li X, Roseman S. The chitinolytic cascade in Vibrios is regulated by chitin oligosaccharides and a two-component chitin catabolic sensor/kinase. *Proc Natl Acad Sci U S A* 2004; **101**: 627–631.

19. Staufenberger T, Imhoff JF, Labes A. First crenarchaeal chitinase found in Sulfolobus tokodaii. *Microbiological Research* 2012; **167**: 262–269.

20. García-Fraga B, Silva AF da, López-Seijas J, Sieiro C. Functional expression and characterization of a chitinase from the marine archaeon Halobacterium salinarum CECT 395 in Escherichia coli. *Appl Microbiol Biotechnol* 2014; **98**: 2133–2143.

21. Fourquez M, Beier S, Jongmans E, Hunter R, Obernosterer I. Uptake of Leucine, Chitin, and Iron by Prokaryotic Groups during Spring Phytoplankton Blooms Induced by Natural Iron Fertilization off Kerguelen Island (Southern Ocean). *Front Mar Sci* 2016; **3**.

22. Tonon T, Li Y, McQueen-Mason S. Mannitol biosynthesis in algae: more widespread and diverse than previously thought. *New Phytol* 2017; **213**: 1573–1579.

23. Reed RH, Davison IR, Chudek JA, Foster R. The osmotic role of mannitol in the Phaeophyta: an appraisal. *Phycologia* 1985; **24**: 35–47.

24. Rosen BP, Liu Z. Transport pathways for arsenic and selenium: A miniriew. *Environ Int* 2009; **35**: 512–515.

25. Dyhrman ST, Haley ST. Arsenate Resistance in the Unicellular Marine Diazotroph Crocosphaera watsonii. *Front Microbiol* 2011; **2**.

26. Ordóñez E, Belle KV, Roos G, Galan SD, Letek M, Gil JA, et al. Arsenate Reductase, Mycothiol, and Mycoredoxin Concert Thiol/Disulfide Exchange. *J Biol Chem* 2009; **284**: 15107–15116.

27. Cuebas M, Villafane A, McBride M, Yee N, Bini E. Arsenate reduction and expression of multiple chromosomal ars operons in Geobacillus kaustophilus A1. *Microbiology* 2011; **157**: 2004–2011.

28. Jung K-H. The distinct signaling mechanisms of microbial sensory rhodopsins in Archaea, Eubacteria and Eukarya. *Photochem Photobiol* 2007; **83**: 63–69.

29. Bamann C, Bamberg E, Wachtveitl J, Glaubitz C. Proteorhodopsin. *Biochimica et Biophysica Acta (BBA) - Bioenergetics* 2014; **1837**: 614–625.

30. Frigaard N-U, Martinez A, Mincer TJ, DeLong EF. Proteorhodopsin lateral gene transfer between marine planktonic Bacteria and Archaea. *Nature* 2006; **439**: 847–850.

31. Baker BJ, Sheik CS, Taylor CA, Jain S, Bhasi A, Cavalcoli JD, et al. Community transcriptomic assembly reveals microbes that contribute to deep-sea carbon and nitrogen cycling. *ISME J* 2013; **7**: 1962–1973.

32. Deschamps P, Zivanovic Y, Moreira D, Rodriguez-Valera F, López-García P. Pangenome Evidence for Extensive Interdomain Horizontal Transfer Affecting Lineage Core and Shell Genes in Uncultured Planktonic Thaumarchaeota and Euryarchaeota. *Genome Biol Evol* 2014; **6**: 1549–1563.

33. Boeuf D, Lami R, Cunnington E, Jeanthon C. Summer Abundance and Distribution of Proteorhodopsin Genes in the Western Arctic Ocean. *Front Microbiol* 2016; **7**.

34. Sabehi G, Loy A, Jung K-H, Partha R, Spudich JL, Isaacson T, et al. New Insights into Metabolic Properties of Marine Bacteria Encoding Proteorhodopsins. *PLOS Biology* 2005; **3**: e273.

35. Béjà O, Aravind L, Koonin EV, Suzuki MT, Hadd A, Nguyen LP, et al. Bacterial Rhodopsin: Evidence for a New Type of Phototrophy in the Sea. *Science* 2000; **289**: 1902–1906.

36. Yutin N, Koonin EV. Proteorhodopsin genes in giant viruses. *Biol Direct* 2012; **7**: 34.

37. Gushchin I, Chervakov P, Kuzmichev P, Popov AN, Round E, Borshchevskiy V, et al. Structural insights into the proton pumping by unusual proteorhodopsin from nonmarine bacteria. *Proc Natl Acad Sci U S A* 2013; **110**: 12631–12636.

38. Pinhassi J, DeLong EF, Béjà O, González JM, Pedrós-Alió C. Marine Bacterial and Archaeal Ion-Pumping Rhodopsins: Genetic Diversity, Physiology, and Ecology. *Microbiol Mol Biol Rev* 2016; **80**: 929–954.

39. Munn C. Marine Microbiology: Ecology & Applications, 2 edition. 2011. Garland Science, New York.

40. Batten ML, Imanishi Y, Maeda T, Tu DC, Moise AR, Bronson D, et al. Lecithin-retinol Acyltransferase Is Essential for Accumulation of All-trans-Retinyl Esters in the Eye and in the Liver. *J Biol Chem* 2004; **279**: 10422–10432.

41. Chasteen TG, Fuentes DE, Tantaleán JC, Vásquez CC. Tellurite: history, oxidative stress, and molecular mechanisms of resistance. *FEMS Microbiol Rev* 2009; **33**: 820–832.

42. Pérez JM, Calderón IL, Arenas FA, Fuentes DE, Pradenas GA, Fuentes EL, et al. Bacterial Toxicity of Potassium Tellurite: Unveiling an Ancient Enigma. *PLoS One* 2007; **2**.

43. Rathgeber C, Yurkova N, Stackebrandt E, Beatty JT, Yurkov V. Isolation of Tellurite- and Selenite-Resistant Bacteria from Hydrothermal Vents of the Juan de Fuca Ridge in the Pacific Ocean. *Appl Environ Microbiol* 2002; **68**: 4613–4622.

44. Coelho C, Romão MJ. Structural and mechanistic insights on nitrate reductases. *Protein Sci* 2015; **24**: 1901–1911.

45. Huang Q, Abdalla AE, Xie J. Phylogenomics of Mycobacterium Nitrate Reductase Operon. *Curr Microbiol* 2015; **71**: 121–128.

46. Rothery RA, Magalon A, Giordano G, Guigliarelli B, Blasco F, Weiner JH. The Molybdenum Cofactor of Escherichia coli Nitrate Reductase A (NarGHI) EFFECT OF A mobAB MUTATION AND INTERACTIONS WITH [Fe-S] CLUSTERS. *J Biol Chem* 1998; **273**: 7462–7469.

47. Sohaskey CD. Nitrate Enhances the Survival of Mycobacterium tuberculosis during Inhibition of Respiration. *J Bacteriol* 2008; **190**: 2981–2986.

48. Sunda W. Feedback Interactions between Trace Metal Nutrients and Phytoplankton in the Ocean. *Front Microbiol* 2012; **3**.

49. Cabello P, Roldán MD, Moreno-Vivián C. Nitrate reduction and the nitrogen cycle in archaea. *Microbiology* 2004; **150**: 3527–3546.

50. Bay DC, Chan CS, Turner RJ. NarJ subfamily system specific chaperone diversity and evolution is directed by respiratory enzyme associations. *BMC Evol Biol* 2015; **15**.

51. Qin W, Amin SA, Martens-Habbena W, Walker CB, Urakawa H, Devol AH, et al. Marine ammonia-oxidizing archaeal isolates display obligate mixotrophy and wide ecotypic variation. *PNAS* 2014; **111**: 12504–12509.
