# Supplementary material for: A phylogenomic and ecological analysis of the globally abundant Marine Group II archaea (Ca. Poseidoniales ord. nov.)
Source: ISME J. 2018 Oct 15;13(3):663–75. doi: 10.1038/s41396-018-0282-y (PMC6461757; doi:10.1038/s41396-018-0282-y)
Supplement: Supplementary file 2 — Supplementary Tables and Figures [file 41396_2018_282_MOESM2_ESM.docx]

**Supplementary Tables and Figures**

**Table S1 | Metagenome-assembled genomes (MAGs) used in this study.** MAGs are ordered by estimated completeness. All MAGs are medium-quality draft assemblies (completeness ≥ 50%; contamination ≤ 10%), except for “Lau93” which qualifies as low-quality draft MAG, according to the minimum information about a metagenome-assembled genome (MISAG) standards [1]. Completeness and contamination estimates are provided for each MAG, calculated with the tool CheckM (see Material and Methods). Note, the MAGs from Li et al. 2015 [2] were obtained from the authors directly (Li. pers. comm.), and not all are available at GenBank. However, the original metagenome sequences are deposited in the DOE JGI-IMG/MER database under the following Taxon Object IDs: 3300001680 (Kilo Moana), 3300001681 (Abe), 3300001678 (Mariner), 3300001679 (Tahi Moana), 3300001676 (Tui Malila), 3300001683 (Guaymas), 3300001781 (Cayman Deep) and 3300001835 (Cayman Shallow). Proposed type species are highlighted in blue. Abbreviations: NCBI organism or isolate name (Organism name); NCBI accession number (Accession no.); internal accession number used during data analysis (Internal acc. no.);method used to recover genome (Method); estimated completeness (Comp); estimated contamination (Cont); genomes size in bp (Size); reference (Ref). Genomes excluded based on quality standard requirements are highlighted with a red background.

| **Organism_name** | **Accession no.** | **Internal acc. no.** | **Method** | **Comp** | **Cont** | **Size** | **Ref** |
| --- | --- | --- | --- | --- | --- | --- | --- |
| Cayman51_deep* | PSPG00000000 | U_62587 | MAG | 92.32 | 0 | 1,479,632 | Li et al., 2015 |
| Cayman80_deep | PSPC00000000 | U_62593 | MAG | 74.74 | 0.87 | 1,322,386 | Li et al., 2015 |
| Guaymas21 | PSPY00000000 | U_62590 | MAG | 87.25 | 2.61 | 1,501,106 | Li et al., 2015 |
| Guaymas22 | PSPX00000000 | U_62589 | MAG | 88.94 | 0.7 | 1,448,805 | Li et al., 2015 |
| Guaymas23 | PSPW00000000 | U_62585 | MAG | 93.77 | 2.61 | 1,633,324 | Li et al., 2015 |
| Guaymas25 | PSPU00000000 | U_62588 | MAG | 91.74 | 8.7 | 2,275,685 | Li et al., 2015 |
| Guaymas26 | PSPT00000000 | U_62592 | MAG | 84.78 | 0.87 | 1,751,774 | Li et al., 2015 |
| Guaymas27 | PSPS00000000 | U_62609 | MAG | 51.3 | 0 | 1,026,877 | Li et al., 2015 |
| Guaymas28 | PSPR00000000 | U_62586 | MAG | 93.04 | 3.11 | 1,921,720 | Li et al., 2015 |
| Guaymas29 | PSPQ00000000 | U_62591 | MAG | 85.9 | 3.02 | 1,597,417 | Li et al., 2015 |
| Lau93 | PSPZ00000000 | U_62613 | MAG | 21.15 | 1.1 | 623,523 | Li et al., 2015 |
| uncultured marine group II euryarchaeote | GCA_000246735.1 | GB_GCA_000246735.1 | MAG | 90.72 | 0 | 2,064,136 | Parks et al., 2018 |
| Marine group II euryarchaeote REDSEA-S03_B6 | GCA_001628435.1 | GB_GCA_001628435.1 | MAG | 70.37 | 2.5 | 1,315,350 | Parks et al., 2018 |
| Marine group II euryarchaeote REDSEA-S10_B2 | GCA_001628455.1 | GB_GCA_001628455.1 | MAG | 83.04 | 0.43 | 1,234,658 | Parks et al., 2018 |
| Marine group II euryarchaeote REDSEA-S19_B7N8 | GCA_001628475.1 | GB_GCA_001628475.1 | MAG | 76.81 | 0.87 | 1,171,691 | Parks et al., 2018 |
| Marine group II euryarchaeote REDSEA-S11_B3N4 | GCA_001628485.1 | GB_GCA_001628485.1 | MAG | 89.37 | 0 | 1,295,986 | Parks et al., 2018 |
| Marine group II euryarchaeote REDSEA-S30_B12 | GCA_001629205.1 | GB_GCA_001629205.1 | MAG | 73.97 | 0.87 | 1,266,629 | Parks et al., 2018 |
| Marine group II euryarchaeote REDSEA-S43_B8 | GCA_001629235.1 | GB_GCA_001629235.1 | MAG | 78.7 | 0 | 1,101,674 | Parks et al., 2018 |
| Marine group II euryarchaeote REDSEA-S41_B6 | GCA_001629245.1 | GB_GCA_001629245.1 | MAG | 77.84 | 1.22 | 1,129,223 | Parks et al., 2018 |
| Marine group II euryarchaeote REDSEA-S42_B7 | GCA_001629255.1 | GB_GCA_001629255.1 | MAG | 80 | 0 | 1,191,311 | Parks et al., 2018 |
| Marine group II euryarchaeote REDSEA-S40_B11N13 | GCA_001629295.1 | GB_GCA_001629295.1 | MAG | 78.22 | 0 | 1,240,878 | Parks et al., 2018 |
| ERX552264.b32.v1 | GCA_002494605.1 | U_76773 | MAG | 87.68 | 0 | 1,758,451 | Parks et al., 2018 |
| SRX802076.b33.v1 | GCA_002494645.1 | U_71923 | MAG | 91.74 | 0 | 1,794,878 | Parks et al., 2018 |
| ERX555967.b49.v1 | GCA_002494685.1 | U_66947 | MAG | 81.54 | 0.87 | 1,654,031 | Parks et al., 2018 |
| ERX552262.b23.v1 | GCA_002494865.1 | U_76730 | MAG | 79.91 | 1.91 | 1,753,015 | Parks et al., 2018 |
| ERX552274.b19.v1 | GCA_002494905.1 | U_75617 | MAG | 78.7 | 0.14 | 1,263,560 | Parks et al., 2018 |
| ERX552274.b13.v1 | GCA_002494965.1 | U_75616 | MAG | 89.42 | 0 | 1,425,612 | Parks et al., 2018 |
| SRX802189.b23.v1 | GCA_002494975.1 | U_76974 | MAG | 74.03 | 0.87 | 1,337,649 | Parks et al., 2018 |
| ERX556009.b33.v1 | GCA_002495045.1 | U_66109 | MAG | 87.62 | 1.74 | 1,905,265 | Parks et al., 2018 |
| ERX556126.b12.v1 | GCA_002495105.1 | U_65615 | MAG | 79.23 | 0 | 1,859,385 | Parks et al., 2018 |
| ERX552241.b8.v1 | GCA_002495165.1 | U_65778 | MAG | 77.68 | 0 | 1,288,480 | Parks et al., 2018 |
| ERX552277.b3.v1 | GCA_002495245.1 | U_75649 | MAG | 67.64 | 0 | 1,182,715 | Parks et al., 2018 |
| ERX552297.b27.v1 | GCA_002495285.1 | U_75481 | MAG | 77.25 | 0.87 | 1,227,335 | Parks et al., 2018 |
| ERX555933.b23.v1 | GCA_002495385.1 | U_69271 | MAG | 72.03 | 0 | 1,286,306 | Parks et al., 2018 |
| ERX556040.b22.v1 | GCA_002495395.1 | U_68723 | MAG | 75.07 | 3.62 | 1,357,804 | Parks et al., 2018 |
| ERX556126.b68.v1 | GCA_002495405.1 | U_65628 | MAG | 78.84 | 4.35 | 2,330,256 | Parks et al., 2018 |
| ERX555967.b11.v1 | GCA_002495525.1 | U_66942 | MAG | 85.76 | 0.87 | 1,385,735 | Parks et al., 2018 |
| SRX672291.b14.v1 | GCA_002495535.1 | U_73977 | MAG | 87.26 | 1.74 | 1,835,100 | Parks et al., 2018 |
| ERX552262.b50.v1 | GCA_002495645.1 | U_76738 | MAG | 87.27 | 0 | 1,848,559 | Parks et al., 2018 |
| ERX556056.b9.v1 | GCA_002495675.1 | U_68843 | MAG | 79.86 | 0 | 1,367,263 | Parks et al., 2018 |
| ERX556139.b6.v1 | GCA_002495735.1 | U_66271 | MAG | 65.22 | 0 | 1,133,618 | Parks et al., 2018 |
| ERX552258.b3.v1 | GCA_002495815.1 | U_77620 | MAG | 64.3 | 0.87 | 1,046,834 | Parks et al., 2018 |
| ERX556108.b12.v1 | GCA_002495925.1 | U_67218 | MAG | 73.91 | 2.17 | 1,123,522 | Parks et al., 2018 |
| ERX556126.b25.v1 | GCA_002495945.1 | U_65618 | MAG | 79.71 | 2.61 | 1,602,356 | Parks et al., 2018 |
| ERX555931.b31.v1 | GCA_002496005.1 | U_69288 | MAG | 67.39 | 0.87 | 1,092,855 | Parks et al., 2018 |
| ERX555966.b9.v1 | GCA_002496175.1 | U_66932 | MAG | 78.12 | 0.87 | 1,639,900 | Parks et al., 2018 |
| ERX552239.b21.v1 | GCA_002496205.1 | U_66627 | MAG | 77.25 | 0.87 | 1,315,197 | Parks et al., 2018 |
| ERX556031.b26.v1 | GCA_002496245.1 | U_67167 | MAG | 72.81 | 0.87 | 1,554,421 | Parks et al., 2018 |
| ERX552262.b15.v1 | GCA_002496285.1 | U_76728 | MAG | 74.49 | 0.87 | 1,703,409 | Parks et al., 2018 |
| SRX672316.b8.v1 | GCA_002496435.1 | U_65863 | MAG | 71.74 | 0 | 1,352,311 | Parks et al., 2018 |
| ERX555978.b29.v1 | GCA_002496485.1 | U_65938 | MAG | 76.07 | 1.3 | 1,344,809 | Parks et al., 2018 |
| ERX555945.merged_b34_b35.v1 | GCA_002496595.1 | U_77936 | MAG | 73 | 1.3 | 1,699,595 | Parks et al., 2018 |
| ERX555961.b44.v1 | GCA_002496635.1 | U_66899 | MAG | 78.8 | 1.65 | 1,617,893 | Parks et al., 2018 |
| ERX556054.b49.v1 | GCA_002496725.1 | U_68838 | MAG | 84.43 | 0.87 | 1,331,106 | Parks et al., 2018 |
| SRX802143.b63.v1 | GCA_002496735.1 | U_67755 | MAG | 91.3 | 0 | 1,568,067 | Parks et al., 2018 |
| ERX552298.b56.v1 | GCA_002496845.1 | U_75537 | MAG | 86.81 | 0.87 | 1,871,345 | Parks et al., 2018 |
| ERX556101.b13.v1 | GCA_002496885.1 | U_67233 | MAG | 72.83 | 0 | 1,393,562 | Parks et al., 2018 |
| ERX556101.b17.v1 | GCA_002496905.1 | U_67234 | MAG | 79.94 | 2.22 | 1,354,098 | Parks et al., 2018 |
| ERX555916.merged_b22_b23.v1 | GCA_002496955.1 | U_67730 | MAG | 88.04 | 3.83 | 1,622,264 | Parks et al., 2018 |
| ERX555931.b65.v1 | GCA_002496985.1 | U_69293 | MAG | 78.98 | 3.61 | 1,822,051 | Parks et al., 2018 |
| ERX555917.merged_b38_b48.v1 | GCA_002497025.1 | U_67720 | MAG | 89.97 | 1.01 | 1,447,310 | Parks et al., 2018 |
| ERX555990.merged_b22_b25.v1 | GCA_002497125.1 | U_75935 | MAG | 78.48 | 0 | 1,347,959 | Parks et al., 2018 |
| ERX552238.b26.v1 | GCA_002497145.1 | U_66643 | MAG | 84.07 | 0.87 | 1,616,078 | Parks et al., 2018 |
| ERX556098.b19.v1 | GCA_002497185.1 | U_70279 | MAG | 70.09 | 0 | 1,217,243 | Parks et al., 2018 |
| ERX555987.b29.v1 | GCA_002497245.1 | U_74120 | MAG | 88.84 | 0 | 1,388,573 | Parks et al., 2018 |
| ERX556127.b15.v1 | GCA_002497265.1 | U_65611 | MAG | 77.41 | 1.74 | 1,301,216 | Parks et al., 2018 |
| ERX555907.b66.v1 | GCA_002497295.1 | U_68603 | MAG | 81.16 | 0 | 1,342,360 | Parks et al., 2018 |
| ERX555954.b29.v1 | GCA_002497315.1 | U_77020 | MAG | 78.94 | 3.48 | 1,340,785 | Parks et al., 2018 |
| ERX552262.b22.v1 | GCA_002497385.1 | U_76729 | MAG | 86.09 | 0.87 | 1,871,923 | Parks et al., 2018 |
| ERX289005.b14.v1 | GCA_002497405.1 | U_66537 | MAG | 84.35 | 0.43 | 1,876,881 | Parks et al., 2018 |
| ERX552256.b27.v1 | GCA_002497465.1 | U_77660 | MAG | 68.55 | 0 | 1,185,813 | Parks et al., 2018 |
| ERX556103.b37.v1 | GCA_002497525.1 | U_67242 | MAG | 73.77 | 0.87 | 1,244,262 | Parks et al., 2018 |
| ERX555949.b21.v1 | GCA_002497545.1 | U_77941 | MAG | 78.79 | 0 | 1,304,134 | Parks et al., 2018 |
| ERX556059.b53.v1 | GCA_002497565.1 | U_68808 | MAG | 85.22 | 0 | 1,815,858 | Parks et al., 2018 |
| ERX556126.b14.v1 | GCA_002497645.1 | U_65617 | MAG | 87.83 | 0.87 | 1,891,970 | Parks et al., 2018 |
| ERX552297.b25.v1 | GCA_002497745.1 | U_75479 | MAG | 90.87 | 3.91 | 1,998,396 | Parks et al., 2018 |
| ERX552297.b28.v1 | GCA_002497765.1 | U_75482 | MAG | 83.29 | 0.87 | 1,802,484 | Parks et al., 2018 |
| ERX552257.b60.v1 | GCA_002497805.1 | U_77648 | MAG | 64.02 | 0 | 1,558,997 | Parks et al., 2018 |
| ERX555947.merged_b33_b34.v1 | GCA_002497845.1 | U_77924 | MAG | 91.07 | 3.04 | 2,787,008 | Parks et al., 2018 |
| ERX556040.b27.v1 | GCA_002497895.1 | U_68724 | MAG | 76.38 | 0 | 1,266,736 | Parks et al., 2018 |
| ERX556060.b48.v1 | GCA_002497905.1 | U_71332 | MAG | 88.63 | 1.74 | 1,756,838 | Parks et al., 2018 |
| ERX556017.merged_b4_b5.v1 | GCA_002497985.1 | U_65529 | MAG | 60.29 | 0 | 925,674 | Parks et al., 2018 |
| ERX555966.b33.v1 | GCA_002498135.1 | U_66934 | MAG | 81.19 | 2.68 | 1,731,776 | Parks et al., 2018 |
| SRX802076.b91.v1 | GCA_002498145.1 | U_71938 | MAG | 79.63 | 3.33 | 1,449,212 | Parks et al., 2018 |
| ERX556126.b3.v1 | GCA_002498185.1 | U_65614 | MAG | 81.9 | 0 | 1,714,656 | Parks et al., 2018 |
| ERX555971.b15.v1 | GCA_002498265.1 | U_65914 | MAG | 65.94 | 2.17 | 1,430,304 | Parks et al., 2018 |
| ERX552256.b16.v1 | GCA_002498455.1 | U_77659 | MAG | 82.4 | 0 | 1,828,059 | Parks et al., 2018 |
| SRX1075082.b48.v1 | GCA_002498525.1 | U_66319 | MAG | 92.26 | 0.87 | 1,581,043 | Parks et al., 2018 |
| ERX556028.b32.v1 | GCA_002498535.1 | U_70152 | MAG | 63.62 | 0 | 1,469,016 | Parks et al., 2018 |
| SRX514549.b35.v1 | GCA_002498585.1 | U_74618 | MAG | 86.96 | 3.48 | 1,362,482 | Parks et al., 2018 |
| SRX514549.b20.v1 | GCA_002498625.1 | U_74615 | MAG | 77.2 | 0 | 1,234,254 | Parks et al., 2018 |
| ERX552243.b35.v1 | GCA_002498725.1 | U_65801 | MAG | 92.46 | 0 | 1,494,957 | Parks et al., 2018 |
| ERX556040.b14.v1 | GCA_002498925.1 | U_68721 | MAG | 74.96 | 0 | 1,287,024 | Parks et al., 2018 |
| ERX555947.b11.v1 | GCA_002498935.1 | U_77917 | MAG | 79.42 | 0 | 1,368,985 | Parks et al., 2018 |
| SRX803008.b63.v1 | GCA_002498945.1 | U_66035 | MAG | 87.73 | 0 | 1,832,746 | Parks et al., 2018 |
| SRX802076.b58.v1 | GCA_002498985.1 | U_71928 | MAG | 75.65 | 0.87 | 1,211,870 | Parks et al., 2018 |
| SRX1050770.b8.v1 | GCA_002499015.1 | U_71063 | MAG | 89.86 | 0 | 1,810,621 | Parks et al., 2018 |
| ERX555984.merged_b16_b17.v1 | GCA_002499195.1 | U_74143 | MAG | 90.43 | 0 | 1,853,906 | Parks et al., 2018 |
| ERX555984.b37.v1 | GCA_002499205.1 | U_74142 | MAG | 84.9 | 0.05 | 1,756,642 | Parks et al., 2018 |
| ERX552256.b10.v1 | GCA_002499265.1 | U_77658 | MAG | 80.87 | 0.87 | 1,340,770 | Parks et al., 2018 |
| SRX1044543.b9.v1 | GCA_002499285.1 | U_68618 | MAG | 70.88 | 0 | 1,225,610 | Parks et al., 2018 |
| ERX555999.b3.v1 | GCA_002499325.1 | U_76148 | MAG | 74.49 | 0 | 1,198,511 | Parks et al., 2018 |
| ERX555907.b26.v1 | GCA_002499345.1 | U_68597 | MAG | 70.53 | 0 | 1,121,012 | Parks et al., 2018 |
| SRX672316.b5.v1 | GCA_002499365.1 | U_65861 | MAG | 74.44 | 0 | 1,460,939 | Parks et al., 2018 |
| ERX555973.b42.v1 | GCA_002499425.1 | U_65906 | MAG | 82.76 | 2.61 | 1,403,855 | Parks et al., 2018 |
| ERX556059.b35.v1 | GCA_002499485.1 | U_68806 | MAG | 72.63 | 0 | 1,302,561 | Parks et al., 2018 |
| ERX555967.b5.v1 | GCA_002499545.1 | U_66941 | MAG | 89.39 | 0 | 1,869,267 | Parks et al., 2018 |
| ERX552298.b2.v1 | GCA_002499555.1 | U_75526 | MAG | 77.93 | 0 | 1,187,150 | Parks et al., 2018 |
| ERX552264.b16.v1 | GCA_002499585.1 | U_76771 | MAG | 78.55 | 0.87 | 1,362,883 | Parks et al., 2018 |
| ERX552275.b10.v1 | GCA_002499595.1 | U_75623 | MAG | 69.84 | 0 | 1,158,939 | Parks et al., 2018 |
| ERX555951.b7.v1 | GCA_002499645.1 | U_76969 | MAG | 65.07 | 0 | 1,059,829 | Parks et al., 2018 |
| ERX555967.b63.v1 | GCA_002499705.1 | U_66950 | MAG | 85.94 | 0 | 1,879,829 | Parks et al., 2018 |
| ERX556017.b1.v1 | GCA_002499715.1 | U_65521 | MAG | 56.92 | 0 | 950,302 | Parks et al., 2018 |
| ERX552238.b28.v1 | GCA_002499745.1 | U_66644 | MAG | 82.46 | 5.22 | 1,476,213 | Parks et al., 2018 |
| ERX556033.b32.v1 | GCA_002499785.1 | U_67149 | MAG | 69.08 | 0.87 | 1,200,070 | Parks et al., 2018 |
| ERX552253.b5.v1 | GCA_002499795.1 | U_77675 | MAG | 86.81 | 0.87 | 1,374,909 | Parks et al., 2018 |
| ERX552255.b33.v1 | GCA_002499835.1 | U_77689 | MAG | 82.52 | 2.17 | 1,754,850 | Parks et al., 2018 |
| ERX555918.b4.v1 | GCA_002499865.1 | U_67692 | MAG | 80.53 | 0.09 | 1,296,940 | Parks et al., 2018 |
| ERX555918.b5.v1 | GCA_002499885.1 | U_67693 | MAG | 78.55 | 0 | 1,701,653 | Parks et al., 2018 |
| ERX552249.b9.v1 | GCA_002501605.1 | U_65838 | MAG | 80 | 0 | 1,287,379 | Parks et al., 2018 |
| ERX552249.b5.v1 | GCA_002501615.1 | U_65837 | MAG | 81.59 | 0 | 1,780,808 | Parks et al., 2018 |
| ERX552268.b18.v1 | GCA_002501685.1 | U_76708 | MAG | 58.15 | 0 | 986,770 | Parks et al., 2018 |
| SRX514548.b54.v1 | GCA_002501805.1 | U_74609 | MAG | 88.26 | 0 | 1,341,925 | Parks et al., 2018 |
| ERX556126.b37.v1 | GCA_002501815.1 | U_65620 | MAG | 67.83 | 0.87 | 1,117,122 | Parks et al., 2018 |
| ERX556028.b28.v1 | GCA_002501885.1 | U_70150 | MAG | 86.55 | 1.74 | 1,306,710 | Parks et al., 2018 |
| ERX556134.b25.v1 | GCA_002501915.1 | U_66230 | MAG | 75.94 | 2.61 | 1,389,035 | Parks et al., 2018 |
| SRX672316.b13.v1 | GCA_002501975.1 | U_65865 | MAG | 69.42 | 0 | 1,287,962 | Parks et al., 2018 |
| ERX555932.b32.v1 | GCA_002502055.1 | U_69304 | MAG | 91.59 | 2.75 | 1,552,899 | Parks et al., 2018 |
| ERX289005.b7.v1 | GCA_002502095.1 | U_66535 | MAG | 84.16 | 0.43 | 1,318,011 | Parks et al., 2018 |
| ERX552241.b17.v1 | GCA_002502175.1 | U_65779 | MAG | 82.03 | 0 | 1,387,308 | Parks et al., 2018 |
| ERX552255.b1.v1 | GCA_002502205.1 | U_77685 | MAG | 71.36 | 0.87 | 1,169,306 | Parks et al., 2018 |
| ERX555914.b40.v1 | GCA_002502215.1 | U_67701 | MAG | 87.1 | 0 | 1,876,148 | Parks et al., 2018 |
| ERX552302.b14.v1 | GCA_002502255.1 | U_69911 | MAG | 56.68 | 0 | 773,327 | Parks et al., 2018 |
| ERX552249.b1.v1 | GCA_002502285.1 | U_65835 | MAG | 82.03 | 0 | 1,358,582 | Parks et al., 2018 |
| ERX555954.b30.v1 | GCA_002502295.1 | U_77021 | MAG | 83.19 | 0 | 1,951,048 | Parks et al., 2018 |
| ERX552257.b12.v1 | GCA_002502325.1 | U_77630 | MAG | 80.29 | 0 | 1,331,600 | Parks et al., 2018 |
| ERX555990.b30.v1 | GCA_002502335.1 | U_75929 | MAG | 77.93 | 0.87 | 1,616,618 | Parks et al., 2018 |
| ERX556103.b3.v1 | GCA_002502365.1 | U_67236 | MAG | 70 | 0 | 1,083,913 | Parks et al., 2018 |
| ERX552254.b10.v1 | GCA_002502385.1 | U_77670 | MAG | 65.41 | 0.94 | 1,162,973 | Parks et al., 2018 |
| ERX555912.b22.v1 | GCA_002502415.1 | U_67739 | MAG | 88.54 | 0.87 | 1,860,416 | Parks et al., 2018 |
| ERX555914.b6.v1 | GCA_002502485.1 | U_67697 | MAG | 87.83 | 4.35 | 1,874,034 | Parks et al., 2018 |
| ERX552303.b31.v1 | GCA_002502555.1 | U_69907 | MAG | 68.26 | 0 | 1,291,569 | Parks et al., 2018 |
| ERX552241.b19.v1 | GCA_002502565.1 | U_65780 | MAG | 83.33 | 0 | 1,401,856 | Parks et al., 2018 |
| SRX802076.b65.v1 | GCA_002502605.1 | U_71930 | MAG | 79.71 | 4.93 | 1,875,748 | Parks et al., 2018 |
| ERX555914.b45.v1 | GCA_002502625.1 | U_67702 | MAG | 87.8 | 0.05 | 1,820,131 | Parks et al., 2018 |
| ERX552252.b17.v1 | GCA_002502655.1 | U_77699 | MAG | 89.86 | 0.32 | 1,441,483 | Parks et al., 2018 |
| ERX556022.b9.v1 | GCA_002502895.1 | U_68044 | MAG | 75.07 | 0 | 1,222,631 | Parks et al., 2018 |
| SRX514547.b52.v1 | GCA_002503015.1 | U_74528 | MAG | 90.97 | 0 | 1,883,601 | Parks et al., 2018 |
| ERX556005.b39.v1 | GCA_002503045.1 | U_66092 | MAG | 70.88 | 1.45 | 1,579,222 | Parks et al., 2018 |
| ERX555966.b19.v1 | GCA_002503055.1 | U_66933 | MAG | 75.46 | 0.87 | 1,278,241 | Parks et al., 2018 |
| SRX802189.b43.v1 | GCA_002503285.1 | U_76979 | MAG | 92.17 | 0 | 1,524,894 | Parks et al., 2018 |
| ERX555984.b26.v1 | GCA_002503295.1 | U_74141 | MAG | 82.47 | 2.89 | 1,702,623 | Parks et al., 2018 |
| SRX1075082.b82.v1 | GCA_002503395.1 | U_66331 | MAG | 75.11 | 0.87 | 1,983,983 | Parks et al., 2018 |
| ERX552244.b3.v1 | GCA_002503485.1 | U_65813 | MAG | 67.35 | 1.74 | 1,101,783 | Parks et al., 2018 |
| ERX556075.b7.v1 | GCA_002503625.1 | U_70229 | MAG | 80.72 | 0.87 | 1,329,435 | Parks et al., 2018 |
| ERX552298.merged_b66_b72.v1 | GCA_002503675.1 | U_75543 | MAG | 74.45 | 1.74 | 1,559,688 | Parks et al., 2018 |
| ERX555931.b10.v1 | GCA_002503725.1 | U_69281 | MAG | 73.12 | 0 | 1,192,836 | Parks et al., 2018 |
| ERX556134.b12.v1 | GCA_002503755.1 | U_66222 | MAG | 80.14 | 0 | 1,393,853 | Parks et al., 2018 |
| ERX556063.b2.v1 | GCA_002504415.1 | U_71353 | MAG | 90.29 | 0 | 1,392,683 | Parks et al., 2018 |
| ERX555987.b7.v1 | GCA_002504435.1 | U_74118 | MAG | 79.57 | 0 | 1,277,296 | Parks et al., 2018 |
| ERX552256.b5.v1 | GCA_002504565.1 | U_77657 | MAG | 77.25 | 0 | 1,369,607 | Parks et al., 2018 |
| ERX556129.b18.v1 | GCA_002504595.1 | U_65706 | MAG | 80 | 0.87 | 1,677,719 | Parks et al., 2018 |
| ERX556032.b65.v1 | GCA_002504675.1 | U_77162 | MAG | 78.63 | 1.45 | 1,606,115 | Parks et al., 2018 |
| ERX556129.b10.v1 | GCA_002504695.1 | U_65705 | MAG | 84.87 | 0.87 | 1,661,706 | Parks et al., 2018 |
| SRX1075082.b15.v1 | GCA_002504755.1 | U_66304 | MAG | 69.1 | 0 | 1,226,547 | Parks et al., 2018 |
| ERX556103.b6.v1 | GCA_002504805.1 | U_67237 | MAG | 76.09 | 1.05 | 1,682,316 | Parks et al., 2018 |
| ERX555951.b28.v1 | GCA_002504825.1 | U_76972 | MAG | 72.9 | 0 | 1,170,955 | Parks et al., 2018 |
| ERX556060.b12.v1 | GCA_002504845.1 | U_71330 | MAG | 82.03 | 0.87 | 1,259,830 | Parks et al., 2018 |
| ERX552254.b6.v1 | GCA_002504905.1 | U_77669 | MAG | 90.29 | 0 | 1,435,715 | Parks et al., 2018 |
| ERX556134.b9.v1 | GCA_002504925.1 | U_66221 | MAG | 62.46 | 0.87 | 1,043,110 | Parks et al., 2018 |
| ERX552254.b4.v1 | GCA_002504935.1 | U_77668 | MAG | 76.22 | 0 | 1,687,991 | Parks et al., 2018 |
| ERX555919.merged_b10_b12.v1 | GCA_002504995.1 | U_67678 | MAG | 82.42 | 0.87 | 1,717,063 | Parks et al., 2018 |
| ERX555963.b15.v1 | GCA_002505155.1 | U_66903 | MAG | 77.73 | 0 | 1,852,275 | Parks et al., 2018 |
| ERX556094.b31.v1 | GCA_002505355.1 | U_70304 | MAG | 81.99 | 1.74 | 1,721,538 | Parks et al., 2018 |
| SRX648501.b2.v1 | GCA_002505385.1 | U_69541 | MAG | 73.04 | 1.74 | 1,281,151 | Parks et al., 2018 |
| SRX802077.merged_b38_b44.v1** | GCA_002505405.1 | U_71921 | MAG | 90.87 | 0 | 1,936,638 | Parks et al., 2018 |
| ERX555941.b46.v1 | GCA_002505415.1 | U_77903 | MAG | 78.39 | 0.87 | 1,966,738 | Parks et al., 2018 |
| ERX555931.b48.v1 | GCA_002505445.1 | U_69291 | MAG | 78.99 | 1.59 | 1,310,548 | Parks et al., 2018 |
| ERX556134.b46.v1 | GCA_002505455.1 | U_66236 | MAG | 83.33 | 2.61 | 1,439,628 | Parks et al., 2018 |
| SRX1075082.b61.v1 | GCA_002505495.1 | U_66326 | MAG | 80.04 | 1.01 | 1,840,727 | Parks et al., 2018 |
| ERX552275.b48.v1 | GCA_002505685.1 | U_75633 | MAG | 78.12 | 1.74 | 1,514,553 | Parks et al., 2018 |
| ERX552297.b34.v1 | GCA_002505695.1 | U_75484 | MAG | 72.03 | 0 | 1,271,260 | Parks et al., 2018 |
| ERX555912.b2.v1 | GCA_002505775.1 | U_67737 | MAG | 87.39 | 0 | 1,352,658 | Parks et al., 2018 |
| ERX556054.b20.v1 | GCA_002505855.1 | U_68836 | MAG | 76.16 | 2.61 | 1,347,668 | Parks et al., 2018 |
| ERX556098.b66.v1 | GCA_002505935.1 | U_70281 | MAG | 77.54 | 0 | 1,620,084 | Parks et al., 2018 |
| SRX1044556.b12.v1 | GCA_002505985.1 | U_67774 | MAG | 77.81 | 0.87 | 1,394,511 | Parks et al., 2018 |
| ERX555932.b40.v1 | GCA_002506005.1 | U_69306 | MAG | 67.68 | 0 | 1,070,543 | Parks et al., 2018 |
| ERX556106.b5.v1 | GCA_002506025.1 | U_67253 | MAG | 64.69 | 0.87 | 1,428,573 | Parks et al., 2018 |
| ERX555913.b12.v1 | GCA_002506065.1 | U_67731 | MAG | 72.13 | 2.61 | 1,164,698 | Parks et al., 2018 |
| ERX556065.b18.v1 | GCA_002506185.1 | U_71328 | MAG | 84.11 | 0.87 | 1,340,827 | Parks et al., 2018 |
| SRX802076.b77.v1 | GCA_002506275.1 | U_71934 | MAG | 73.62 | 0 | 1,167,799 | Parks et al., 2018 |
| ERX552275.b30.v1 | GCA_002506305.1 | U_75628 | MAG | 92.46 | 0.87 | 1,496,470 | Parks et al., 2018 |
| ERX556103.b43.v1 | GCA_002506385.1 | U_67244 | MAG | 83.1 | 0 | 1,812,406 | Parks et al., 2018 |
| ERX552243.b13.v1 | GCA_002506405.1 | U_65799 | MAG | 70.18 | 0 | 1,183,021 | Parks et al., 2018 |
| ERX555932.b2.v1 | GCA_002506495.1 | U_69301 | MAG | 70.24 | 0.58 | 1,077,973 | Parks et al., 2018 |
| ERX552262.merged_b16_b17.v1 | GCA_002506675.1 | U_76752 | MAG | 73.04 | 0 | 1,191,638 | Parks et al., 2018 |
| ERX552262.b14.v1 | GCA_002506725.1 | U_76727 | MAG | 74.55 | 2.9 | 1,530,732 | Parks et al., 2018 |
| SRX514548.b12.v1 | GCA_002506755.1 | U_74603 | MAG | 76.33 | 0 | 1,245,738 | Parks et al., 2018 |
| ERX556101.b5.v1 | GCA_002506825.1 | U_67232 | MAG | 84.65 | 0 | 1,308,442 | Parks et al., 2018 |
| ERX556005.b69.v1 | GCA_002506875.1 | U_66094 | MAG | 85.8 | 1.78 | 1,799,184 | Parks et al., 2018 |
| SRX147858.b55.v1 | GCA_002507125.1 | U_75882 | MAG | 67.59 | 1.01 | 1,111,706 | Parks et al., 2018 |
| ERX552257.b39.v1 | GCA_002507145.1 | U_77638 | MAG | 61.74 | 0 | 1,065,346 | Parks et al., 2018 |
| ERX555954.b18.v1 | GCA_002507165.1 | U_77019 | MAG | 74.76 | 0.94 | 1,727,560 | Parks et al., 2018 |
| ERX555954.b17.v1 | GCA_002507175.1 | U_77018 | MAG | 83.33 | 1.74 | 1,436,899 | Parks et al., 2018 |
| ERX556059.b12.v1 | GCA_002507225.1 | U_68805 | MAG | 68.19 | 0 | 1,496,899 | Parks et al., 2018 |
| ERX555990.b18.v1 | GCA_002507305.1 | U_75925 | MAG | 83.33 | 0 | 1,369,362 | Parks et al., 2018 |
| ERX556035.b17.v1 | GCA_002507345.1 | U_67132 | MAG | 74.78 | 0.87 | 1,343,516 | Parks et al., 2018 |
| ERX556130.b13.v1 | GCA_002507425.1 | U_66258 | MAG | 87.48 | 0 | 1,731,072 | Parks et al., 2018 |
| bin_112 | QQRW00000000 | U_43171 | MAG | 88.05 | 0.87 | 2,023,763 | this study |
| bin_120 | QQRX00000000 | U_43173 | MAG | 78.22 | 0 | 1,365,509 | this study |
| bin_137 | QQRY00000000 | U_43176 | MAG | 91.45 | 2.75 | 1,972,716 | this study |
| bin_141 | QQRZ00000000 | U_43177 | MAG | 83.14 | 1.16 | 1,761,681 | this study |
| bin_156 | QQSA00000000 | U_43182 | MAG | 88.72 | 0.2 | 1,762,979 | this study |
| bin_195 | QQSB00000000 | U_43193 | MAG | 80.49 | 3.48 | 1,363,896 | this study |
| bin_220 | QQSC00000000 | U_43198 | MAG | 88.85 | 0 | 1,945,193 | this study |
| bin_245 | QQSD00000000 | U_43203 | MAG | 82.34 | 0.87 | 1,781,359 | this study |
| bin_248 | QQSE00000000 | U_43204 | MAG | 83.27 | 0 | 1,863,875 | this study |
| bin_275 | QQSF00000000 | U_43208 | MAG | 83.03 | 3.04 | 1,806,210 | this study |
| bin_32 | QQSG00000000 | U_43216 | MAG | 81.39 | 2.61 | 1,764,012 | this study |
| bin_341 | QQSH00000000 | U_43217 | MAG | 81.16 | 1.81 | 1,865,550 | this study |
| bin_35 | QQSI00000000 | U_43220 | MAG | 78.26 | 2.61 | 1,759,155 | this study |
| bin_375 | QQSJ00000000 | U_43222 | MAG | 77.85 | 2.26 | 1,697,761 | this study |
| bin_62 | QQSK00000000 | U_43234 | MAG | 95.69 | 0 | 1,893,503 | this study |
| ERX288947.b54.v1 | DQIS00000000 | U_67492 | MAG | 74.49 | 0.87 | 1,181,274 | this study/Parks et al., 2018 |
| ERX289004.b45.v1 | DQIT00000000 | U_66560 | MAG | 68.21 | 1.74 | 1,566,165 | this study/Parks et al., 2018 |
| ERX289005.b67.v1 | DQIU00000000 | U_66547 | MAG | 71.3 | 0 | 1,972,512 | this study/Parks et al., 2018 |
| ERX552240.b80.v1 | DQIV00000000 | U_65776 | MAG | 74.37 | 0 | 1,090,207 | this study/Parks et al., 2018 |
| ERX552253.b77.v1 | DQIW00000000 | U_77684 | MAG | 63.55 | 1.74 | 1,530,572 | this study/Parks et al., 2018 |
| ERX552255.b41.v1 | DQIX00000000 | U_77692 | MAG | 64.5 | 0.92 | 1,079,162 | this study/Parks et al., 2018 |
| ERX552262.b91.v1 | DQIY00000000 | U_76746 | MAG | 77.97 | 4.35 | 2,161,899 | this study/Parks et al., 2018 |
| ERX552288.b83.v1 | DQIZ00000000 | U_76279 | MAG | 79.5 | 2.61 | 1,724,762 | this study/Parks et al., 2018 |
| ERX552296.b38.v1 | DQJA00000000 | U_75501 | MAG | 66.3 | 0.43 | 1,460,542 | this study/Parks et al., 2018 |
| ERX552298.b6.v1 | DQJB00000000 | U_75528 | MAG | 68.87 | 0 | 1,467,048 | this study/Parks et al., 2018 |
| ERX552298.b77.v1 | DQJC00000000 | U_75540 | MAG | 65.13 | 1.74 | 1,726,134 | this study/Parks et al., 2018 |
| ERX555907.b115.v1 | DQJD00000000 | U_68608 | MAG | 80.28 | 1.74 | 1,646,433 | this study/Parks et al., 2018 |
| ERX555917.b35.v1 | DQJE00000000 | U_67710 | MAG | 68.46 | 0.87 | 1,063,227 | this study/Parks et al., 2018 |
| ERX555917.b73.v1 | DQJF00000000 | U_67718 | MAG | 71.33 | 1.74 | 1,590,362 | this study/Parks et al., 2018 |
| ERX555918.b8.v1 | DQJG00000000 | U_67694 | MAG | 76.83 | 0 | 1,758,403 | this study/Parks et al., 2018 |
| ERX555919.b17.v1 | DQJH00000000 | U_67673 | MAG | 75.82 | 0.17 | 1,572,985 | this study/Parks et al., 2018 |
| ERX555919.b3.v1 | DQJI00000000 | U_67671 | MAG | 70.08 | 0.11 | 1,111,187 | this study/Parks et al., 2018 |
| ERX555925.b6.v1 | DQJJ00000000 | U_69992 | MAG | 80.14 | 0 | 1,875,471 | this study/Parks et al., 2018 |
| ERX555941.b26.v1 | DQJK00000000 | U_77901 | MAG | 70.59 | 0.92 | 1,694,500 | this study/Parks et al., 2018 |
| ERX555949.b82.v1 | DQJL00000000 | U_77961 | MAG | 68.76 | 1.04 | 1,248,962 | this study/Parks et al., 2018 |
| ERX555957.b85.v1 | DQJM00000000 | U_77027 | MAG | 66.07 | 0.87 | 1,545,626 | this study/Parks et al., 2018 |
| ERX555959.b26.v1 | DQJN00000000 | U_77038 | MAG | 66.91 | 0.94 | 1,154,143 | this study/Parks et al., 2018 |
| ERX555959.b9.v1 | DQJO00000000 | U_77033 | MAG | 74.35 | 1.74 | 1,661,056 | this study/Parks et al., 2018 |
| ERX555964.b14.v1 | DQJP00000000 | U_66911 | MAG | 66.15 | 0 | 1,546,448 | this study/Parks et al., 2018 |
| ERX555964.b19.v1 | DQJQ00000000 | U_66913 | MAG | 55.87 | 0 | 1,065,655 | this study/Parks et al., 2018 |
| ERX555967.b126.v1 | DQJR00000000 | U_66958 | MAG | 69.33 | 0 | 1,176,641 | this study/Parks et al., 2018 |
| ERX555990.b63.v1 | DQJS00000000 | U_75934 | MAG | 68.95 | 0.87 | 1,237,449 | this study/Parks et al., 2018 |
| ERX556003.b26.v1 | DQJT00000000 | U_66075 | MAG | 76.91 | 1.74 | 1,603,141 | this study/Parks et al., 2018 |
| ERX556005.b37.v1 | DQJU00000000 | U_66091 | MAG | 62.33 | 0 | 1,140,228 | this study/Parks et al., 2018 |
| ERX556019.b18.v1 | DQJV00000000 | U_65543 | MAG | 64.17 | 0 | 1,557,874 | this study/Parks et al., 2018 |
| ERX556027.b78.v1 | DQJW00000000 | U_68079 | MAG | 74.17 | 1.59 | 1,667,845 | this study/Parks et al., 2018 |
| ERX556028.b46.v1 | DQJX00000000 | U_70157 | MAG | 76.38 | 0.92 | 1,721,152 | this study/Parks et al., 2018 |
| ERX556028.merged_b30_b31.v1 | DQJY00000000 | U_70165 | MAG | 79.39 | 0 | 1,609,577 | this study/Parks et al., 2018 |
| ERX556031.b19.v1 | DQJZ00000000 | U_67166 | MAG | 70.31 | 0 | 1,295,602 | this study/Parks et al., 2018 |
| ERX556049.b15.v1 | DQKA00000000 | U_69589 | MAG | 73.94 | 0 | 1,701,483 | this study/Parks et al., 2018 |
| ERX556049.b16.v1 | DQKB00000000 | U_69590 | MAG | 76.98 | 0 | 1,598,946 | this study/Parks et al., 2018 |
| ERX556056.b6.v1 | DQKC00000000 | U_68842 | MAG | 60.72 | 0 | 1,576,841 | this study/Parks et al., 2018 |
| ERX556103.b32.v1 | DQKD00000000 | U_67239 | MAG | 77.39 | 0.87 | 1,742,852 | this study/Parks et al., 2018 |
| ERX556134.b26.v1 | DQKE00000000 | U_66231 | MAG | 77.75 | 1.74 | 1,585,995 | this study/Parks et al., 2018 |
| ERX943719.b45.v1 | DQKF00000000 | U_70103 | MAG | 78.59 | 0.92 | 1,297,152 | this study/Parks et al., 2018 |
| SRX802074.b99.v1 | DQKG00000000 | U_71981 | MAG | 69.48 | 0.05 | 1,042,332 | this study/Parks et al., 2018 |
| SRX959616.b14.v1 | DQKH00000000 | U_67560 | MAG | 82.9 | 0 | 1,484,247 | this study/Parks et al., 2018 |
| SRX959616.b5.v1 | DQKI00000000 | U_67557 | MAG | 86.23 | 4.85 | 1,565,062 | this study/Parks et al., 2018 |
| ERX552256.b59.v1 | DQKJ00000000 | U_77664 | MAG | 59.93 | 0.32 | 1,304,347 | this study/Parks et al., 2018 |
| ERX555941.b57.v1 | DQKK00000000 | U_77904 | MAG | 62.99 | 1.27 | 1,424,734 | this study/Parks et al., 2018 |
| ERX555957.b4.v1 | GCA_002503905.1 | U_77023 | MAG | 67.48 | 2.54 | 1,559,320 | Parks et al., 2018 |
| ERX556017.b97.v1 | DQKL00000000 | U_65526 | MAG | 65.45 | 0.87 | 1,231,224 | this study/Parks et al., 2018 |
| Guaymas32 | PSPN00000000 |  | MAG | *95.79* | *90.67* | 2,392,464 | Li et al., 2015 |
| Lau6 | PSQA00000000 |  | MAG | *94.07* | *236.57* | 7,417,145 | Li et al., 2015 |
| Thalassoarchaea marina | 2645728163 |  | fosmids | *73.87* | *13.6* | 1,361,082 | Martin-Cuadrado et al., 2015 |
| Thalassoarchaea mediterranii | 2645728164 |  | fosmids | *73.1* | *75.33* | 2,573,677 | Martin-Cuadrado et al., 2015 |
| Cayman59_shallow | PSPF00000000 |  | MAG | *91.12* | *74.01* | 2,600,802 | Li et al., 2015 |
| Guaymas24 | PSPV00000000 |  | MAG | *88.79* | *14.8* | 2,402,520 | Li et al., 2015 |
| Lau19 | pers. comm. Li Meng |  | MAG | *87.5* | *610.98* | 15,219,040 | Li et al., 2015 |
| Cayman117_shallow | PSPI00000000 |  | MAG | *86.65* | *45.66* | 1,486,040 | Li et al., 2015 |
| Guaymas96 | PSPJ00000000 |  | MAG | *77.36* | *34.63* | 1,086,991 | Li et al., 2015 |
| Lau92 | pers. comm. Li Meng |  | MAG | *76.01* | *112.08* | 2,780,614 | Li et al., 2015 |
| Lau34 | PSQB00000000 |  | MAG | *60.93* | *65.42* | 3,085,883 | Li et al., 2015 |
| Cayman68_shallow | PSPE00000000 |  | MAG | *58.15* | *17.05* | 1,381,458 | Li et al., 2015 |
| Guaymas69 | PSPK00000000 |  | MAG | *55.18* | *29.77* | 3,000,321 | Li et al., 2015 |
| Cayman69_deep | PSPD00000000 |  | MAG | *46.99* | *11.2* | 1,095,695 | Li et al., 2015 |
| Guaymas30 | PSPP00000000 |  | MAG | *9.38* | *10.19* | 1,248,559 | Li et al., 2015 |

* type species *Candidatus* Thalassarchaeum betae (PSPG00000000)

** type species *Candidatus* Poseidonia alphae (GCA_002505405.1)

**Table S2 | Set of 122 archaeal marker proteins used for the phylogenomic inference.** The 122 archaeal proteins were identified as being present in ≥90% of bacterial or archaeal genomes and, when present, single-copy in ≥95% of genomes. The protein sequences were tested for congruency to avoid proteins affected by horizontal gene transfer (see Methods).

| Marker ID | Name | Description | Length (aa) |
| --- | --- | --- | --- |
| PF01990.12 | ATP-synt_F | ATP synthase (F/14-kDa) subunit | 95 |
| PF01866.12 | Diphthamide_syn | Putative diphthamide synthesis protein | 307 |
| PF04104.9 | DNA_primase_lrg | Eukaryotic and archaeal DNA primase, large subunit | 260 |
| PF01984.15 | dsDNA_bind | Double-stranded DNA-binding domain | 107 |
| PF02006.11 | DUF137 | Protein of unknown function DUF137 | 178 |
| PF04019.7 | DUF359 | Protein of unknown function (DUF359) | 121 |
| PF01864.12 | DUF46 | Putative integral membrane protein DUF46 | 175 |
| PF04919.7 | DUF655 | Protein of unknown function (DUF655) | 181 |
| PF07541.7 | EIF_2_alpha | Eukaryotic translation initiation factor 2 alpha subunit | 114 |
| PF13685.1 | Fe-ADH_2 | Iron-containing alcohol dehydrogenase | 250 |
| PF01269.12 | Fibrillarin | Fibrillarin | 229 |
| PF00368.13 | HMG-CoA_red | Hydroxymethylglutaryl-coenzyme A reductase | 373 |
| PF01798.13 | Nop | Putative snoRNA binding domain | 150 |
| PF00687.16 | Ribosomal_L1 | Ribosomal protein L1p/L10e family | 220 |
| PF00466.15 | Ribosomal_L10 | Ribosomal protein L10 | 100 |
| PF00827.12 | Ribosomal_L15e | Ribosomal L15 | 192 |
| PF01280.15 | Ribosomal_L19e | Ribosomal protein L19e | 148 |
| PF01157.13 | Ribosomal_L21e | Ribosomal protein L21e | 99 |
| PF01198.14 | Ribosomal_L31e | Ribosomal protein L31e | 83 |
| PF01655.13 | Ribosomal_L32e | Ribosomal protein L32 | 110 |
| PF01090.14 | Ribosomal_S19e | Ribosomal protein S19e | 140 |
| PF01282.14 | Ribosomal_S24e | Ribosomal protein S24e | 84 |
| PF01200.13 | Ribosomal_S28e | Ribosomal protein S28e | 69 |
| PF01015.13 | Ribosomal_S3Ae | Ribosomal S3Ae family | 195 |
| PF00900.15 | Ribosomal_S4e | Ribosomal family S4e | 77 |
| PF01092.14 | Ribosomal_S6e | Ribosomal protein S6e | 127 |
| PF00410.14 | Ribosomal_S8 | Ribosomal protein S8 | 129 |
| PF01000.21 | RNA_pol_A_bac | RNA polymerase Rpb3/RpoA insert domain | 112 |
| PF13656.1 | RNA_pol_L_2 | RNA polymerase Rpb3/Rpb11 dimerisation domain | 77 |
| PF01194.12 | RNA_pol_N | RNA polymerases N / 8 kDa subunit | 60 |
| PF03874.11 | RNA_pol_Rpb4 | RNA polymerase Rpb4 | 117 |
| PF01191.14 | RNA_pol_Rpb5_C | RNA polymerase Rpb5, C-terminal domain | 74 |
| PF02978.14 | SRP_SPB | Signal peptide binding domain | 104 |
| PF01868.11 | UPF0086 | Domain of unknown function UPF0086 | 89 |
| PF01496.14 | V_ATPase_I | V-type ATPase 116kDa subunit family | 759 |
| TIGR00021 | rpiA | ribose 5-phosphate isomerase A | 218 |
| TIGR00037 | eIF_5A | translation elongation factor IF5A | 130 |
| TIGR00042 | TIGR00042 | non-canonical purine NTP pyrophosphatase, RdgB/HAM1 family | 184 |
| TIGR00064 | ftsY | signal recognition particle-docking protein FtsY | 279 |
| TIGR00111 | pelota | mRNA surveillance protein pelota | 351 |
| TIGR00134 | gatE_arch | glutamyl-tRNA(Gln) amidotransferase, subunit E | 622 |
| TIGR00240 | ATCase_reg | aspartate carbamoyltransferase, regulatory subunit | 150 |
| TIGR00264 | TIGR00264 | alpha-NAC homolog | 116 |
| TIGR00270 | TIGR00270 | TIGR00270 family protein | 154 |
| TIGR00279 | uL16_euk_arch | ribosomal protein uL16 | 172 |
| TIGR00283 | arch_pth2 | peptidyl-tRNA hydrolase | 115 |
| TIGR00291 | RNA_SBDS | rRNA metabolism protein, SBDS family | 231 |
| TIGR00293 | TIGR00293 | prefoldin, alpha subunit | 129 |
| TIGR00307 | eS8 | ribosomal protein eS8 | 127 |
| TIGR00308 | TRM1 | N2,N2-dimethylguanosine tRNA methyltransferase | 375 |
| TIGR00323 | eIF-6 | putative translation initiation factor eIF-6 | 215 |
| TIGR00324 | endA | tRNA-intron lyase | 177 |
| TIGR00335 | primase_sml | putative DNA primase, eukaryotic-type, small subunit | 324 |
| TIGR00336 | pyrE | orotate phosphoribosyltransferase | 173 |
| TIGR00337 | PyrG | CTP synthase | 526 |
| TIGR00373 | TIGR00373 | transcription factor E | 162 |
| TIGR00389 | glyS_dimeric | glycine--tRNA ligase | 565 |
| TIGR00392 | ileS | isoleucine--tRNA ligase | 861 |
| TIGR00398 | metG | methionine--tRNA ligase | 530 |
| TIGR00405 | KOW_elon_Spt5 | transcription elongation factor Spt5 | 145 |
| TIGR00408 | proS_fam_I | proline--tRNA ligase | 475 |
| TIGR00422 | valS | valine--tRNA ligase | 863 |
| TIGR00425 | CBF5 | putative rRNA pseudouridine synthase | 322 |
| TIGR00432 | arcsn_tRNA_tgt | tRNA-guanine(15) transglycosylase | 637 |
| TIGR00442 | hisS | histidine--tRNA ligase | 406 |
| TIGR00448 | rpoE | DNA-directed RNA polymerase | 179 |
| TIGR00456 | argS | arginine--tRNA ligase | 569 |
| TIGR00458 | aspS_nondisc | aspartate--tRNA(Asn) ligase | 428 |
| TIGR00463 | gltX_arch | glutamate--tRNA ligase | 560 |
| TIGR00468 | pheS | phenylalanine--tRNA ligase, alpha subunit | 324 |
| TIGR00471 | pheT_arch | phenylalanine--tRNA ligase, beta subunit | 551 |
| TIGR00490 | aEF-2 | translation elongation factor aEF-2 | 720 |
| TIGR00491 | aIF-2 | translation initiation factor aIF-2 | 594 |
| TIGR00501 | met_pdase_II | methionine aminopeptidase, type II | 295 |
| TIGR00521 | coaBC_dfp | phosphopantothenoylcysteine decarboxylase / phosphopantothenate--cysteine ligase | 392 |
| TIGR00522 | dph5 | diphthine synthase | 258 |
| TIGR00549 | mevalon_kin | mevalonate kinase | 276 |
| TIGR00658 | orni_carb_tr | ornithine carbamoyltransferase | 304 |
| TIGR00670 | asp_carb_tr | aspartate carbamoyltransferase | 304 |
| TIGR00729 | TIGR00729 | ribonuclease HII | 207 |
| TIGR00936 | ahcY | adenosylhomocysteinase | 416 |
| TIGR00982 | uS12_E_A | ribosomal protein uS12 | 139 |
| TIGR01008 | uS3_euk_arch | ribosomal protein uS3 | 195 |
| TIGR01012 | uS2_euk_arch | ribosomal protein uS2 | 196 |
| TIGR01018 | uS4_arch | ribosomal protein uS4 | 162 |
| TIGR01020 | uS5_euk_arch | ribosomal protein uS5 | 212 |
| TIGR01025 | uS19_arch | ribosomal protein uS19 | 135 |
| TIGR01028 | uS7_euk_arch | ribosomal protein uS7 | 186 |
| TIGR01038 | uL22_arch_euk | ribosomal protein uL22 | 148 |
| TIGR01046 | uS10_euk_arch | ribosomal protein uS10 | 99 |
| TIGR01052 | top6b | DNA topoisomerase VI, B subunit | 488 |
| TIGR01060 | eno | phosphopyruvate hydratase | 425 |
| TIGR01077 | L13_A_E | ribosomal protein uL13 | 141 |
| TIGR01080 | rplX_A_E | ribosomal protein uL24 | 116 |
| TIGR01213 | pseudo_Pus10arc | tRNA pseudouridine(54/55) synthase | 387 |
| TIGR01309 | uL30_arch | ribosomal protein uL30 | 151 |
| TIGR01952 | nusA_arch | NusA family KH domain protein, archaeal | 141 |
| TIGR02076 | pyrH_arch | putative uridylate kinase | 222 |
| TIGR02153 | gatD_arch | glutamyl-tRNA(Gln) amidotransferase, subunit D | 405 |
| TIGR02236 | recomb_radA | DNA repair and recombination protein RadA | 311 |
| TIGR02258 | 2_5_ligase | 2'-5' RNA ligase | 180 |
| TIGR02338 | gimC_beta | prefoldin, beta subunit | 110 |
| TIGR02389 | RNA_pol_rpoA2 | DNA-directed RNA polymerase, subunit A'' | 367 |
| TIGR02390 | RNA_pol_rpoA1 | DNA-directed RNA polymerase subunit A' | 867 |
| TIGR02651 | RNase_Z | ribonuclease Z | 302 |
| TIGR03626 | L3_arch | ribosomal protein uL3 | 331 |
| TIGR03627 | uS9_arch | ribosomal protein uS9 | 130 |
| TIGR03628 | arch_S11P | ribosomal protein uS11 | 117 |
| TIGR03629 | uS13_arch | ribosomal protein uS13 | 144 |
| TIGR03636 | uL23_arch | ribosomal protein uL23 | 77 |
| TIGR03653 | uL6_arch | ribosomal protein uL6 | 170 |
| TIGR03665 | arCOG04150 | arCOG04150 universal archaeal KH domain protein | 173 |
| TIGR03670 | rpoB_arch | DNA-directed RNA polymerase subunit B | 599 |
| TIGR03671 | cca_archaeal | CCA-adding enzyme | 410 |
| TIGR03672 | rpl4p_arch | 50S ribosomal protein uL4 | 251 |
| TIGR03673 | uL14_arch | 50S ribosomal protein uL14 | 131 |
| TIGR03674 | fen_arch | flap structure-specific endonuclease | 338 |
| TIGR03677 | eL8_ribo | ribosomal protein eL8 | 117 |
| TIGR03680 | eif2g_arch | translation initiation factor 2, gamma subunit | 407 |
| TIGR03683 | A-tRNA_syn_arch | alanine--tRNA ligase | 902 |
| TIGR03684 | arCOG00985 | arCOG04150 universal archaeal PUA-domain protein | 154 |
| TIGR03722 | arch_KAE1 | universal archaeal protein Kae1 | 323 |

**Table S3 | Metagenomes from which MAGs were obtained.** The 270 MAGs were recovered from 141 metagenomes (SRA experiments) from worldwide sampling locations at different depths.

| # | SRA Experiment | # MAGs | Depth [m] | Latitude | Longitude | Temperature [deg C] | Oxygen [umol/kg] |
| --- | --- | --- | --- | --- | --- | --- | --- |
| 1 | ERX288947 | 1 | 5 | 37.05 | 1.94 | 23.83 | n. a. |
| 2 | ERX289004 | 1 | 42 | 37.05 | 1.95 | 17.37 | n. a. |
| 3 | ERX289005 | 3 | 5 | 37.05 | 1.94 | 23.83 | n. a. |
| 4 | ERX552238 | 2 | 5 | -9 | -139.2 | 26.54 | 186.24 |
| 5 | ERX552239 | 1 | 120 | -9.07 | -140.6 | 25.22 | 175.9 |
| 6 | ERX552240 | 1 | 5 | 18.4 | 39.88 | 27.6 | 184.06 |
| 7 | ERX552241 | 3 | 5 | -9.15 | -140.52 | 26.52 | 190.72 |
| 8 | ERX552243 | 2 | 115 | -9.01 | -139.14 | 24.7 | 179.92 |
| 9 | ERX552244 | 1 | 50 | -31.03 | 4.68 | 16.79 | 231.68 |
| 10 | ERX552249 | 3 | 140 | -8.9 | -142.55 | 23.71 | 160.49 |
| 11 | ERX552252 | 1 | 120 | -30.15 | -43.27 | 19.31 | 217.14 |
| 12 | ERX552253 | 2 | 120 | -30.15 | -43.27 | 19.31 | 217.14 |
| 13 | ERX552254 | 3 | 5 | -30.14 | -43.29 | 19.85 | 221.47 |
| 14 | ERX552255 | 3 | 5 | -20.41 | -3.18 | 19.78 | 215.65 |
| 15 | ERX552256 | 5 | 140 | -8.9 | -142.55 | 23.71 | 160.49 |
| 16 | ERX552257 | 3 | 5 | -9.15 | -140.52 | 26.52 | 190.72 |
| 17 | ERX552258 | 1 | 50 | -31.03 | 4.68 | 16.79 | 231.68 |
| 18 | ERX552262 | 7 | 5 | -8.91 | -140.28 | n. a. | 189.76 |
| 19 | ERX552264 | 2 | 5 | -9 | -139.2 | n. a. | 186.24 |
| 20 | ERX552268 | 1 | 150 | -8.91 | -140.28 | 22.12 | 161.32 |
| 21 | ERX552274 | 2 | 5 | -30.14 | -43.29 | 19.85 | 221.47 |
| 22 | ERX552275 | 3 | 115 | -9.01 | -139.14 | 24.7 | 179.92 |
| 23 | ERX552277 | 1 | 5 | -31.03 | 4.67 | 16.83 | 231.91 |
| 24 | ERX552288 | 1 | 5 | 39.16 | 5.92 | 23.85 | n. a. |
| 25 | ERX552296 | 1 | 5 | -20.94 | -35.18 | 23.35 | 206.19 |
| 26 | ERX552297 | 4 | 5 | -8.91 | -140.28 | 26.58 | 189.76 |
| 27 | ERX552298 | 5 | 5 | -20.41 | -3.18 | 19.78 | 215.65 |
| 28 | ERX552302 | 1 | 5 | -31.03 | 4.67 | 16.83 | 231.91 |
| 29 | ERX552303 | 1 | 55 | 39.06 | 5.94 | n. a. | n. a. |
| 30 | ERX555907 | 3 | 45 | 35.4 | -127.75 | 13.16 | 227.69 |
| 31 | ERX555912 | 2 | 5 | 27.16 | 27.14 | 25.06 | 188.76 |
| 32 | ERX555913 | 1 | 40 | 36.55 | -6.57 | 20.41 | n. a. |
| 33 | ERX555914 | 3 | 5 | 35.76 | 14.26 | 21.44 | 207.79 |
| 34 | ERX555916 | 1 | 5 | -13 | -95.98 | 25.25 | 200.23 |
| 35 | ERX555917 | 3 | 50 | -12.97 | -96.01 | 20.65 | 216.79 |
| 36 | ERX555918 | 3 | 5 | -20.41 | -3.18 | 19.78 | 215.65 |
| 37 | ERX555919 | 3 | 5 | -23.28 | -129.39 | 24.24 | 202.17 |
| 38 | ERX555925 | 1 | 5 | -47.19 | -58.29 | 7.32 | 305.01 |
| 39 | ERX555931 | 4 | 80 | 23.42 | 37.25 | n. a. | 179.97 |
| 40 | ERX555932 | 3 | 115 | -9.01 | -139.14 | 24.7 | 179.92 |
| 41 | ERX555933 | 1 | 5 | -16.96 | -100.63 | 22.77 | 208.87 |
| 42 | ERX555941 | 3 | 50 | -1.9 | -84.63 | 21.81 | 144.63 |
| 43 | ERX555945 | 1 | 5 | -17.02 | 42.74 | 26.97 | 190.05 |
| 44 | ERX555947 | 2 | 5 | -9 | -139.2 | 26.54 | 186.24 |
| 45 | ERX555949 | 2 | 177 | -12.98 | -96.02 | 13.01 | 0.71 |
| 46 | ERX555951 | 2 | 5 | 43.77 | -16.9 | n. a. | n. a. |
| 47 | ERX555954 | 4 | 5 | -5.25 | -85.15 | 24.94 | 205.99 |
| 48 | ERX555957 | 2 | 5 | 36.17 | -29.02 | n. a. | 232.05 |
| 49 | ERX555959 | 2 | 150 | -21.03 | -35.35 | 21.64 | 203.81 |
| 50 | ERX555961 | 1 | 60 | 6.34 | -102.95 | 19.05 | 77.04 |
| 51 | ERX555963 | 1 | 5 | 1.99 | -84.58 | 27.62 | 198.64 |
| 52 | ERX555964 | 2 | 5 | 36.55 | -6.57 | 20.53 | n. a. |
| 53 | ERX555966 | 3 | 155 | -23.22 | -129.5 | 22.26 | 207.09 |
| 54 | ERX555967 | 5 | 5 | 35.37 | -127.74 | 19.25 | 224.41 |
| 55 | ERX555971 | 1 | 5 | -15.34 | 43.3 | 27.32 | 193.06 |
| 56 | ERX555973 | 1 | 590 | 39.24 | -70.03 | 5.07 | 230.25 |
| 57 | ERX555978 | 1 | 5 | 18.4 | 39.88 | 27.6 | 184.06 |
| 58 | ERX555984 | 3 | 5 | 31.69 | -64.25 | 20.4 | 212.56 |
| 59 | ERX555987 | 2 | 100 | -8.73 | -17.96 | 24.11 | 194.43 |
| 60 | ERX555990 | 4 | 5 | 0 | -153.68 | 26.07 | 179.86 |
| 61 | ERX555999 | 1 | 5 | -20.94 | -35.18 | 23.35 | 206.19 |
| 62 | ERX556003 | 1 | 5 | 34.11 | -49.92 | 18.73 | 220.19 |
| 63 | ERX556005 | 3 | 40 | -5.27 | -85.27 | 19.57 | 103.92 |
| 64 | ERX556009 | 1 | 5 | -8.78 | -17.91 | 25.03 | 199.12 |
| 65 | ERX556017 | 3 | 115 | 31.52 | -159.05 | 15.28 | 225.5 |
| 66 | ERX556019 | 1 | 17 | 20.82 | 63.51 | 25.37 | 211.61 |
| 67 | ERX556022 | 1 | 5 | 23.36 | 37.22 | 25.82 | 188.42 |
| 68 | ERX556027 | 1 | 600 | 20.85 | 63.59 | 12.07 | 1.64 |
| 69 | ERX556028 | 4 | 5 | 25.53 | -88.39 | 24.97 | 194.27 |
| 70 | ERX556031 | 2 | 5 | 14.2 | -116.63 | 26.41 | 195.13 |
| 71 | ERX556032 | 1 | 50 | 39.4 | 19.4 | 15.16 | 229.55 |
| 72 | ERX556033 | 1 | 650 | 35.27 | -127.73 | n. a. | 8.58 |
| 73 | ERX556035 | 1 | 5 | 21.95 | 38.25 | 27.33 | 182.81 |
| 74 | ERX556040 | 3 | 80 | 36.18 | -28.94 | 16.75 | 228.55 |
| 75 | ERX556049 | 2 | 5 | -25.81 | -111.72 | 25.15 | 200.54 |
| 76 | ERX556054 | 2 | 5 | 43.68 | -16.83 | 14.28 | 243.15 |
| 77 | ERX556056 | 2 | 5 | -9.39 | 66.42 | 29.82 | 187.26 |
| 78 | ERX556059 | 3 | 5 | 34.67 | -71.31 | n. a. | 214.37 |
| 79 | ERX556060 | 2 | 90 | -16.96 | -100.68 | 19.88 | 211.55 |
| 80 | ERX556063 | 1 | 75 | -16.95 | 53.96 | 24.89 | 192.56 |
| 81 | ERX556065 | 1 | 188 | -25.83 | -111.73 | 20.17 | 210.58 |
| 82 | ERX556075 | 1 | 120 | -30.15 | -43.27 | 19.31 | 217.14 |
| 83 | ERX556094 | 1 | 5 | 31.52 | -159 | 25.17 | 197.68 |
| 84 | ERX556098 | 2 | 80 | 6 | 73.91 | 27.72 | 132.78 |
| 85 | ERX556101 | 3 | 5 | 39.23 | -70.04 | 14.12 | 233.86 |
| 86 | ERX556103 | 5 | 125 | 25.62 | -88.45 | 24.95 | 192.97 |
| 87 | ERX556106 | 1 | 5 | 6.33 | -102.94 | 26.64 | 196.88 |
| 88 | ERX556108 | 1 | 250 | 34.15 | -56.97 | 18.17 | 191.75 |
| 89 | ERX556126 | 6 | 5 | -8.91 | -140.28 | 26.58 | 189.76 |
| 90 | ERX556127 | 1 | 5 | -29.5 | 37.99 | n. a. | 210.01 |
| 91 | ERX556129 | 2 | 5 | -22.34 | 40.34 | 25.08 | 199.9 |
| 92 | ERX556130 | 1 | 5 | -29.72 | -101.16 | 23.8 | 204.14 |
| 93 | ERX556134 | 5 | 40 | 0.02 | -153.69 | 26.08 | 176.98 |
| 94 | ERX556139 | 1 | 790 | -61.97 | -49.5 | n. a. | 203.84 |
| 95 | ERX943719 | 1 | 16 | 46.23 | -124.16 | n. a. | n. a. |
| 96 | SRX1044543 | 1 | 50 | 18.54 | -81.72 | n. a. | n. a. |
| 97 | SRX1044556 | 1 | 4946 | 18.55 | -81.72 | n. a. | n. a. |
| 98 | SRX1050770 | 1 | 2 | 56.93 | 17.06 | n. a. | n. a. |
| 99 | SRX1075082 | 4 | 4900 | 18.54 | -81.72 | n. a. | n. a. |
| 100 | SRX1097577 | 1 | 25 | 18.58 | 40.74 | n. a. | n. a. |
| 101 | SRX1097585 | 1 | 100 | 20.53 | 38.78 | 0.8 | n. a. |
| 102 | SRX1097597 | 1 | 50 | 23.6 | 37.05 | 0.8 | n. a. |
| 103 | SRX1097608 | 1 | 10 | 27.9 | 34.51 | n. a. | n. a. |
| 104 | SRX1097610 | 1 | 25 | 27.9 | 34.51 | 1.92 | n. a. |
| 105 | SRX1097611 | 1 | 50 | 27.9 | 34.51 | n. a. | n. a. |
| 106 | SRX1097612 | 1 | 100 | 27.9 | 34.51 | n. a. | n. a. |
| 107 | SRX1097619 | 1 | 10 | 18.58 | 40.74 | 1.2 | n. a. |
| 108 | SRX147858 | 1 | 1800 | -50.8 | 37.03 | n. a. | n. a. |
| 109 | SRX514547 | 1 | 50 | 31.4 | -64.1 | n. a. | n. a. |
| 110 | SRX514548 | 2 | 50 | 31.4 | -64.1 | n. a. | n. a. |
| 111 | SRX514549 | 2 | 50 | 31.4 | -64.1 | n. a. | n. a. |
| 112 | SRX648501 | 1 | 80 | 18.8 | -104.7 | n. a. | n. a. |
| 113 | SRX672291 | 1 | 50 | 38.07 | 0.23 | n. a. | n. a. |
| 114 | SRX672316 | 3 | 75 | 38.07 | -0.23 | n. a. | n. a. |
| 115 | SRX802074 | 1 | 1 | 54.18 | 7.9 | n. a. | n. a. |
| 116 | SRX802076 | 5 | 1 | 54.18 | 7.9 | n. a. | n. a. |
| 117 | SRX802077 | 1 | 1 | 54.18 | 7.9 | n. a. | n. a. |
| 118 | SRX802143 | 1 | 1 | 54.17 | 7.9 | n. a. | n. a. |
| 119 | SRX802189 | 2 | 1 | 54.17 | 7.9 | n. a. | n. a. |
| 120 | SRX803008 | 1 | 1 | 54.18 | 7.9 | n. a. | n. a. |
| 121 | SRX959616 | 2 | 90 | 63.44 | 10.43 | n. a. | n. a. |
| 122 | SRX1097587 | 1 | 47 | 17.66 | 40.91 | 2.3 | n. a. |
| 123 | AHCG00000000.1 | 1 | 1 | 47.69 | -122.4 | n. a. | n. a. |
| 124 | SRX1075081 | 2 | 4100 | 18.55 | 81.72 | n. a. | n. a. |
| 125 | SRX1795236 | 8 | 1850 | 27.52 | -111.43 | n. a. | n. a. |
| 126 | SRX511269 | 1 | 2100 | -22.76 | -176.19 | n. a. | n. a. |
| 127 | Port Hacking July 2012 TJ4 | 16 | 2 | -34.12 | 151.23 | 17.59 | 235.77 |
| 128 | Port Hacking August 2012 TJ5 | 16 | 2 | -34.12 | 151.23 | 17.52 | 233.85 |
| 129 | Port Hacking September 2012 TJ6 | 16 | 2 | -34.12 | 151.23 | 16.63 | 234.56 |
| 130 | Port Hacking October 2012 TJ7 | 16 | 2 | -34.12 | 151.23 | 17.51 | 244.83 |
| 131 | Port Hacking November 2012 TJ8 | 16 | 2 | -34.12 | 151.23 | 18.99 | 238.63 |
| 132 | Port Hacking December 2012 TJ9 | 16 | 2 | -34.12 | 151.23 | 20 | 243.28 |
| 133 | Port Hacking January 2013 TJ10 | 16 | 2 | -34.12 | 151.23 | 22.27 | 227.31 |
| 134 | Port Hacking February 2013 TJ11 | 16 | 2 | -34.12 | 151.23 | 17.94 | 187.21 |
| 135 | Port Hacking March 2013 TJ12 | 16 | 2 | -34.12 | 151.23 | 24.54 | 209.81 |
| 136 | Port Hacking April 2013 TJ13 | 16 | 2 | -34.12 | 151.23 | 20.93 | 217.14 |
| 137 | Port Hacking May 2013 TJ14 | 16 | 2 | -34.12 | 151.23 | 20.61 | 209.83 |
| 138 | Port Hacking June 2013 TJ15 | 16 | 2 | -34.12 | 151.23 | 19.65 | 220.53 |
| 139 | Port Hacking July 2013 TJ16 | 16 | 2 | -34.12 | 151.23 | 17.48 | 229.8 |
| 140 | Port Hacking August 2013 TJ17 | 16 | 2 | -34.12 | 151.23 | 17.67 | 226.64 |
| 141 | Port Hacking September 2013 TJ18 | 16 | 2 | -34.12 | 151.23 | 17.75 | 236.64 |

**Table S4 | Core carbon metabolism of *Ca*. Poseidoniales.** Genes aligned to the KEGG database are shown for each genus**.** Heatmap displays the percentage of MAGs per genus possessing a gene assigned to a KO (KEGG Orthology) identifier.


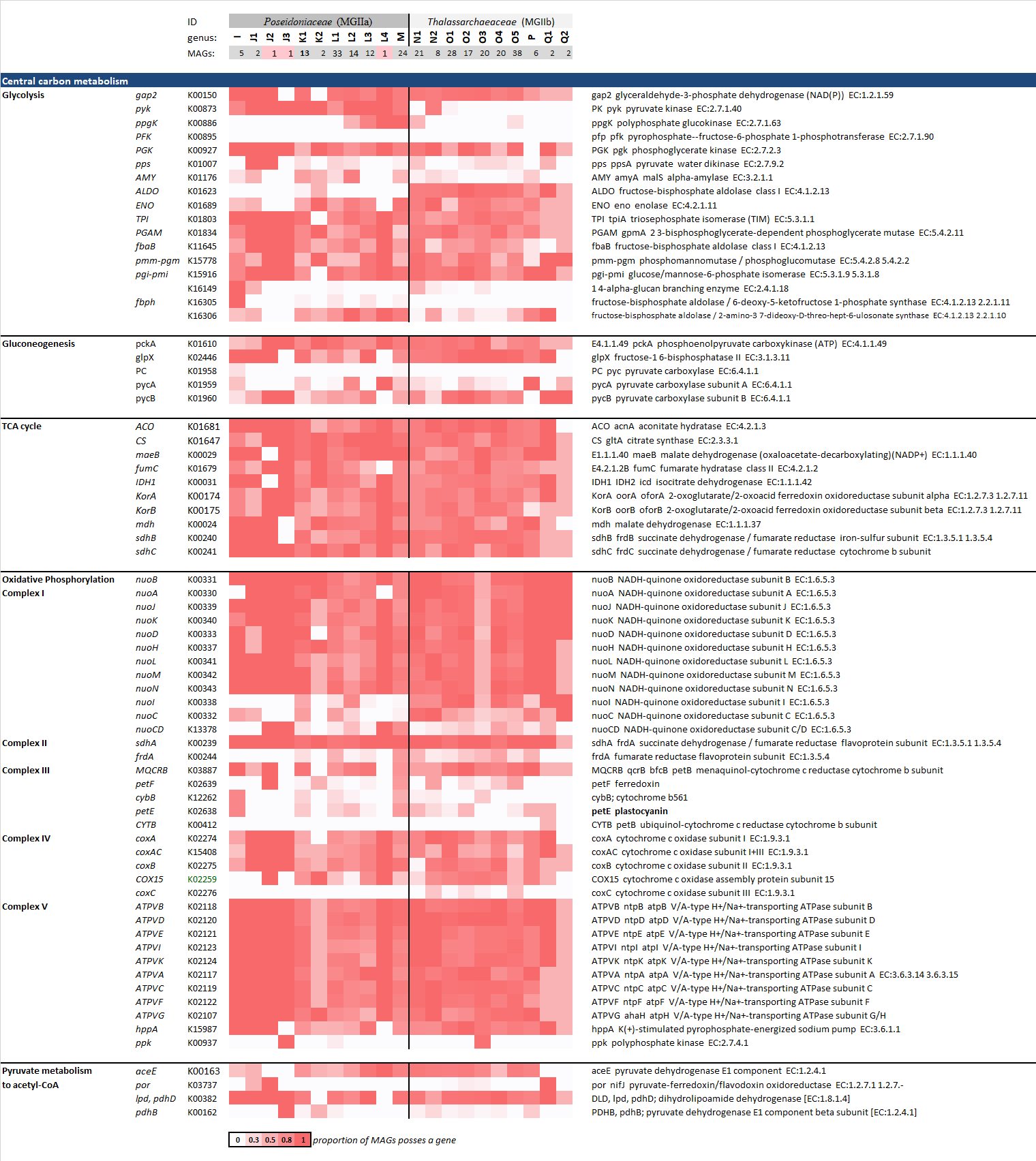


**Table S5 | Amino acid degrading enzymes encoded by *Ca*. Poseidoniales*.*** Shown is the percentage of MAGs per genus to which a KO (KEGG Orthology) identifier has been assigned.

**Table S6 | Peptide degrading enzymes encoded by *Ca*. Poseidoniales*.*** Heatmap *s*hows the average number of gene copies per MAG for each genus. Gene assignments are based on blast searches against the peptide database MEROPS.


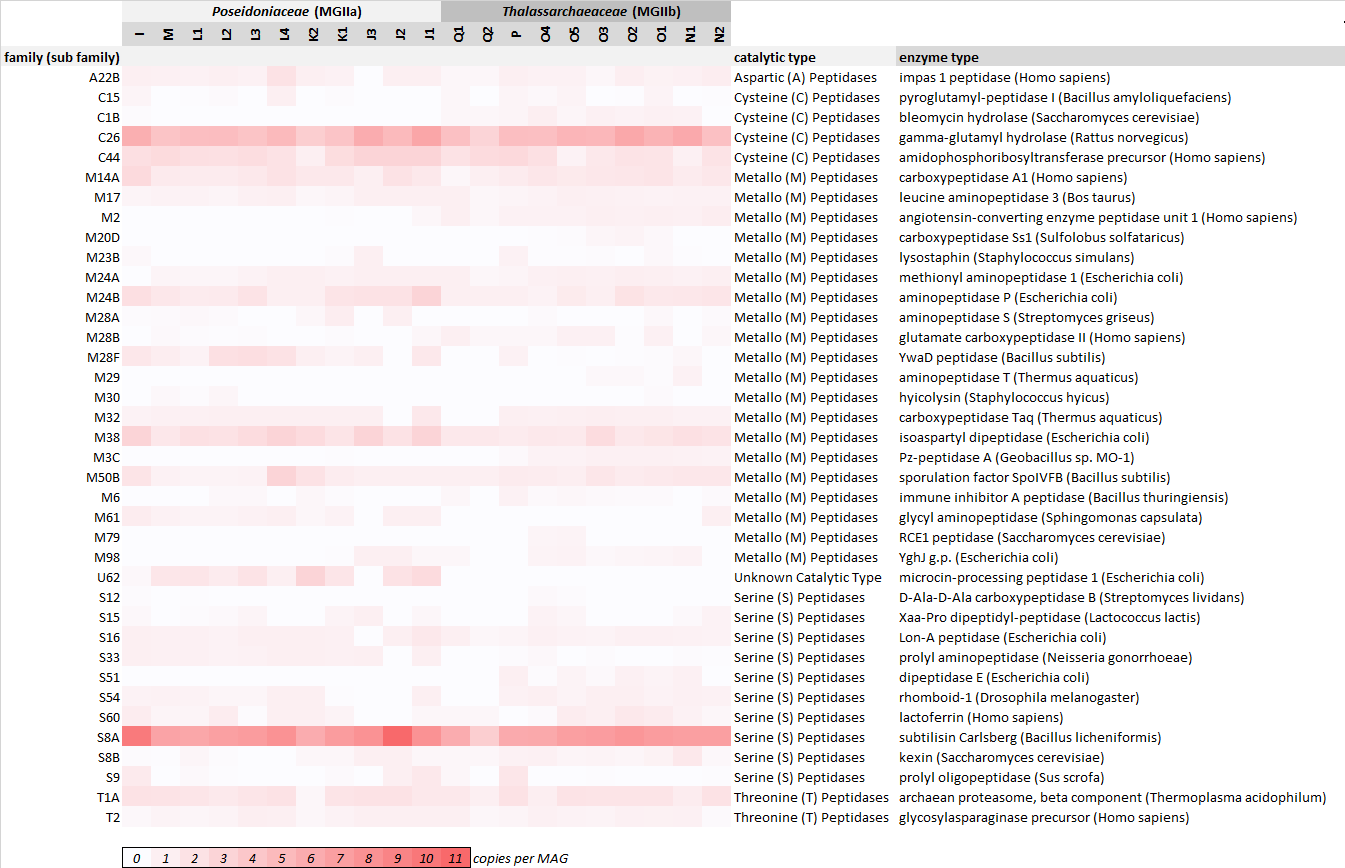


**
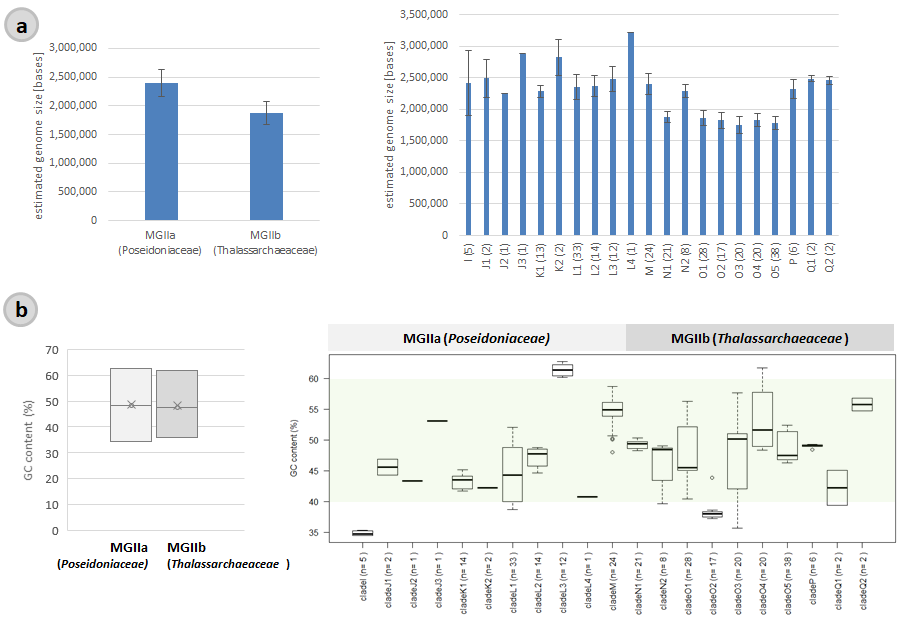
**

**Figure S1 | Genome features. (a) Average estimated genome size.** Average estimated genome sizes, calculated by dividing the MAG size by the estimated completeness and then multiplying by 100, are shown for MGIIa (*Ca*. Poseidoniaceae) and MGIIb (*Ca*. Thalassarchaeaceae), respectively in the left graph. The right graph shows the estimated genome size per clade (genus). **(b) Genomic GC content.** The genome GC content is shown for MGIIa (*Ca*. Poseidoniaceae) and MGIIb (*Ca*. Thalassarchaeaceae), respectively, in the left graph (minimum, average, maximum). The right graph shows the GC content per clade (genus). Note, the GC content ranged from 34.5% to 62.7% with similar means (48.4±7.3; 47.6±5.4) for MGIIa (*Ca*. Poseidoniaceae) and MGIIb (*Ca*. Thalassarchaeaceae), respectively. Clade I (genus I) showed the lowest and clade L3 (genus L3) the highest average GC content (34.9±0.4; 61.4±0.96). Both belonged to subgroup MGIIa (*Ca*. Poseidoniaceae). In MGIIb (*Ca*. Thalassarchaeaceae) clade O2 (genus O2) showed the lowest (38.6±2.0) and clade Q2 (genus Q2) the highest (55.8±1.5) average GC content. Box plot (right graph) shows the median (thick black bar), the first and third quartile (box), and the minimum and maximum GC content values (whiskers).


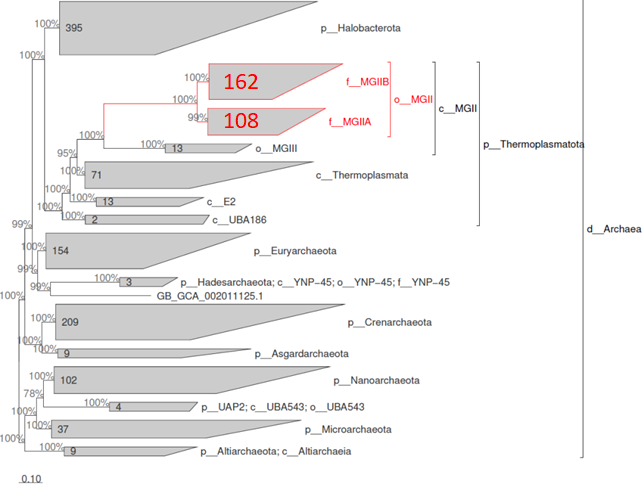


**Figure S2 | Single-copy marker gene tree of the domain Archaea.** The maximum likelihood tree, calculated with FastTree, is based on a multiple sequence alignment of 122 archaeal single-copy marker genes. The taxonomy follows the Genome Taxonomy Database (GTDB;<http://gtdb.ecogenomic.org>). Marine group II (MGII; *Ca*. Poseidoniales) clades are highlighted in red.

**
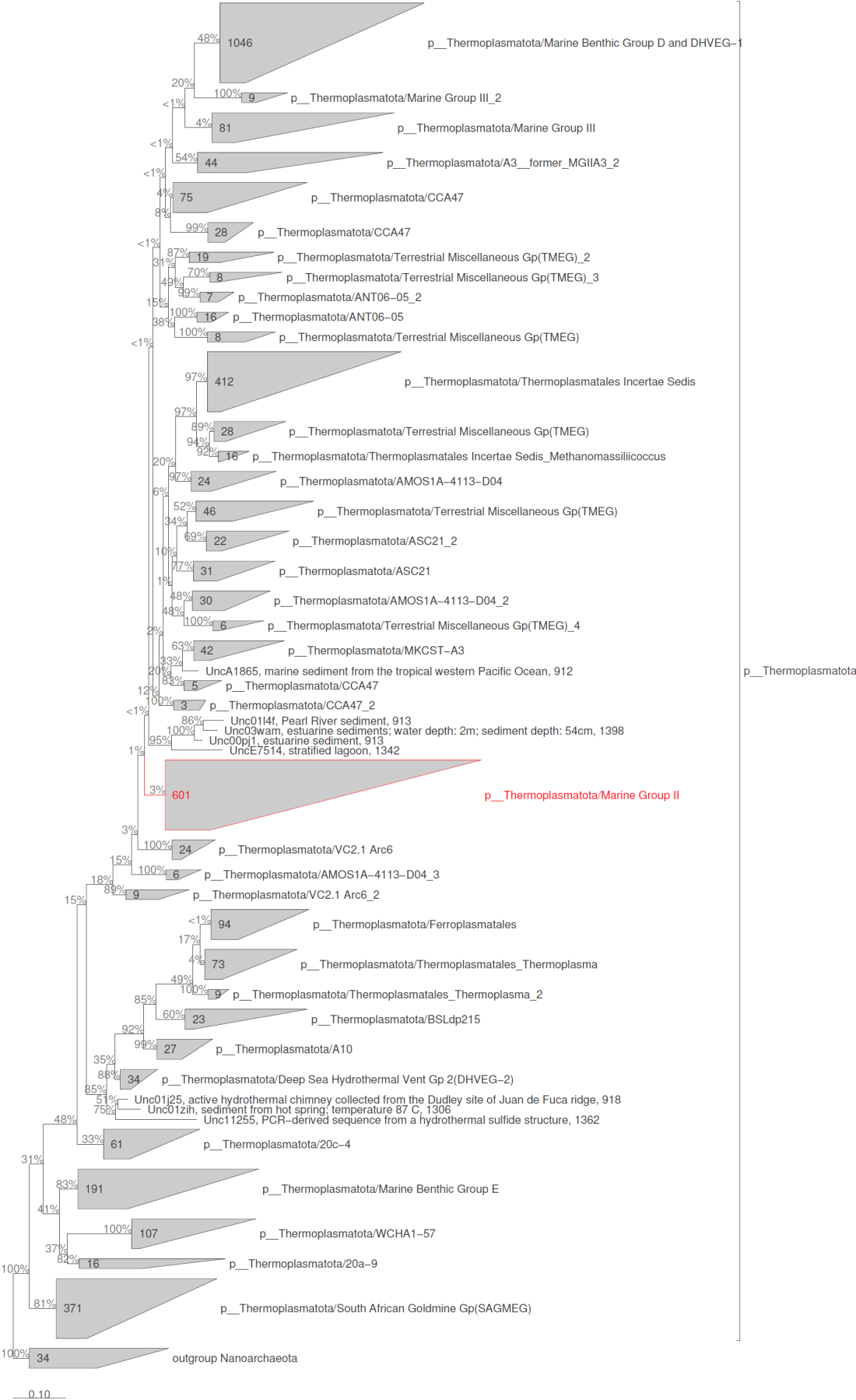
****Figure S3 | SSU rRNA phylogeny.** The bootstrapped SSU tree of *Thermoplasmatota* shows the placement of the 43 SSU sequences (> 700bp) extracted from the MGII MAGs. All sequences were placed within the “Marine Group II” clade (highlighted in red font). The taxonomy is based on SILVA release “SSURef Nr 99 123.1”. The scale bar represents 0.1 substitutions per site.


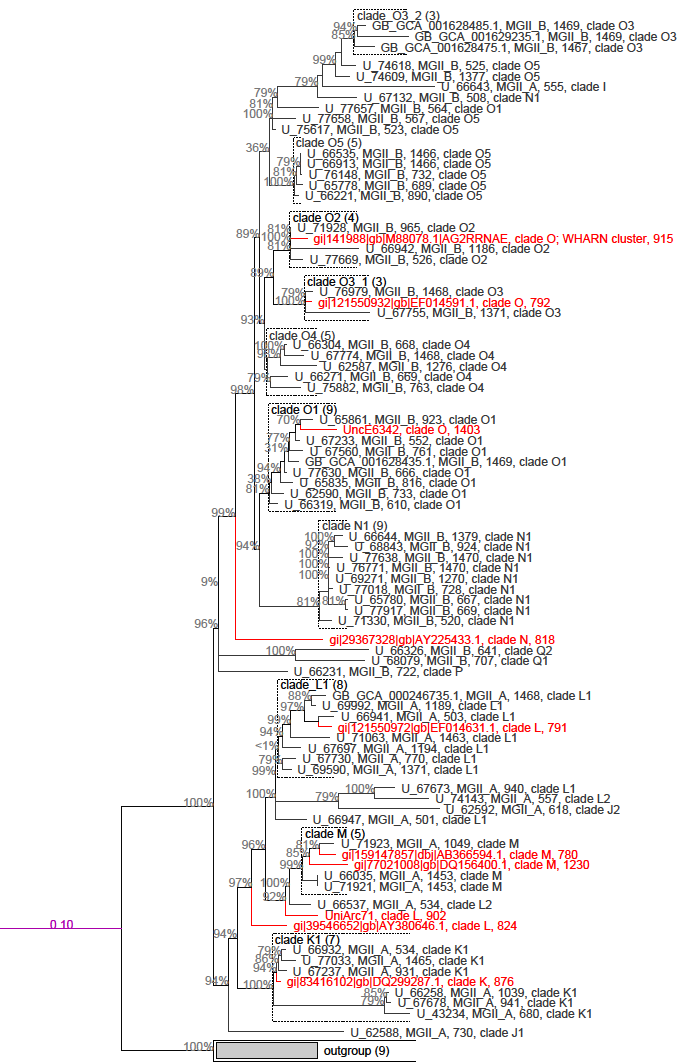


**Figure S4 | SSU rRNA based clade assignments.** The maximum likelihood tree (FastTree, CAT) was calculated using 43 SSU rRNA sequences (>700bp) extracted from MGII MAGs; 10 reference sequences used in Galand et al. 2010 obtained from NCBI and SILVA, and 9 outgroup (E2, MGIII) sequences, resulting in 62 sequences in total. The tree was rooted and 24 MGII SSU sequences greater than 500bp and smaller than 700bp were added to the tree, using the parsimony quick add tool in ARB. Reference sequences are highlighted in red, and the assigned clade names are provided for each group. The scale bar represents 0.1 substitutions per site. Genome IDs are internal accession numbers used during data analysis; see **Table S1**.

**
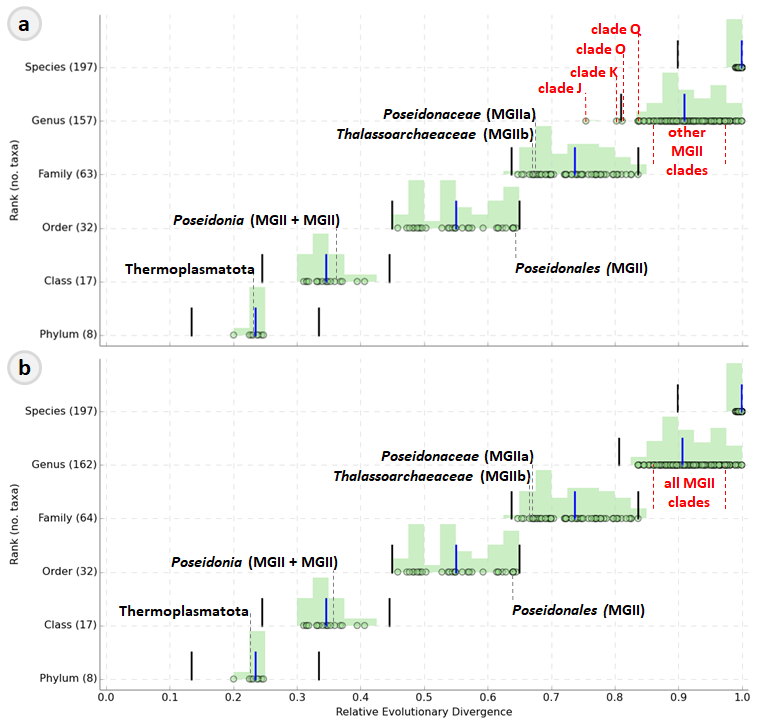
**

**Figure S5 | Rank normalization based on relative evolutionary divergence (RED).** The relative evolutionary divergence (RED; x-axes) is shown for all internal tree nodes with assigned taxonomic ranks (y-axes). Rank assignments are based on the Genome Taxonomy Database (GTDB). **(a) Initial clade distribution:** RED values calculated for all MGII clades indicate that J, K, O, and Q have RED values that are too low for the genus rank, while the remaining clades “other MGII clades” (distribution is indicated by two red dashes lines) are placed well within the genus range. **(b) Distribution after clade splits:** clades J, K, O, and Q were split into multiple groups (e.g. J1, J2, J3) and the RED values were re-calculated. Now all MGII clades (distribution indicated by two red dashes lines) are placed well within the genus range. RED values of the proposed families *Ca*. Poseidoniaceae fam. nov. (MGIIa) and *Ca*. Thalassarchaeaceae fam. nov. (MGIIb), the order *Ca*. Poseidoniales ord. nov. (MGII), the class *Ca*. Poseidoniia class nov. (MGII & MGII), and the phylum *Thermoplasmatota* are highlighted with labelled dashed black lines. The vertical blue bar indicates the median RED value of each rank and the two black lines demark ±0.1 from the median.


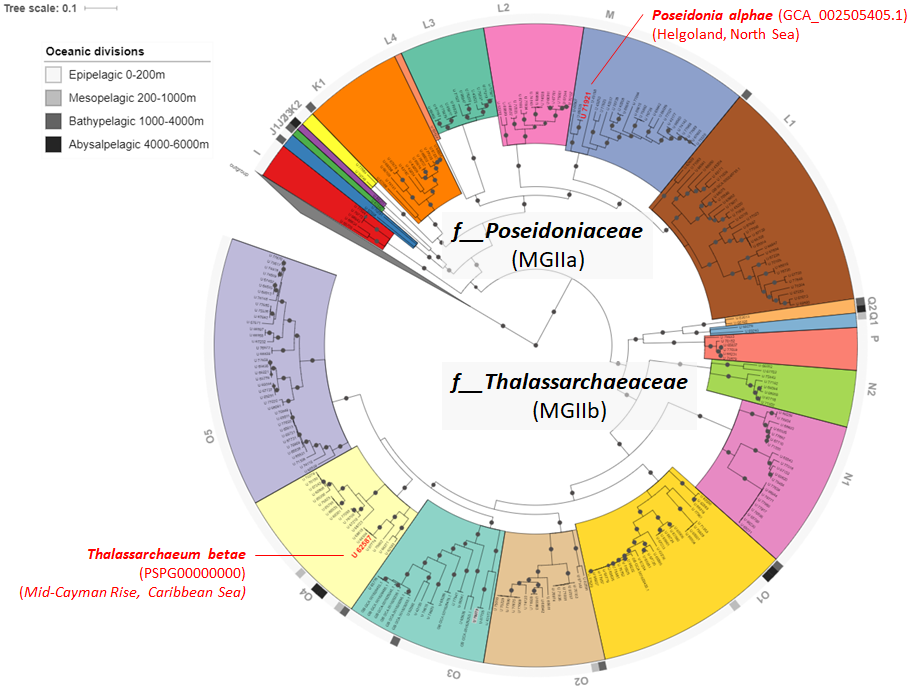


**Figure S6 | Genome tree of the class *Ca*. Poseidoniales.** Phylogenomic tree (FastTree, WAG gamma) calculated from a concatenated alignment of 122 archaeal marker proteins of 302 taxa, including the outgroup. All 270 MAGs are shown and the proposed type species *Poseidonia alpha* (fam. nov. *Ca*. Poseidoniaceae; MGIIa) and *Ca*. Thalassarchaeum *beta* (fam. nov. *Ca*. Thalassarchaeaceae; MGIIb) are highlighted in red. The grey colour bar indicates the sampling depth of each MAG, ranging from epipelagic (0-200m) to Abysalpelagic (4000-6000m). The scale bar represents 0.1 substitutions per site.

**
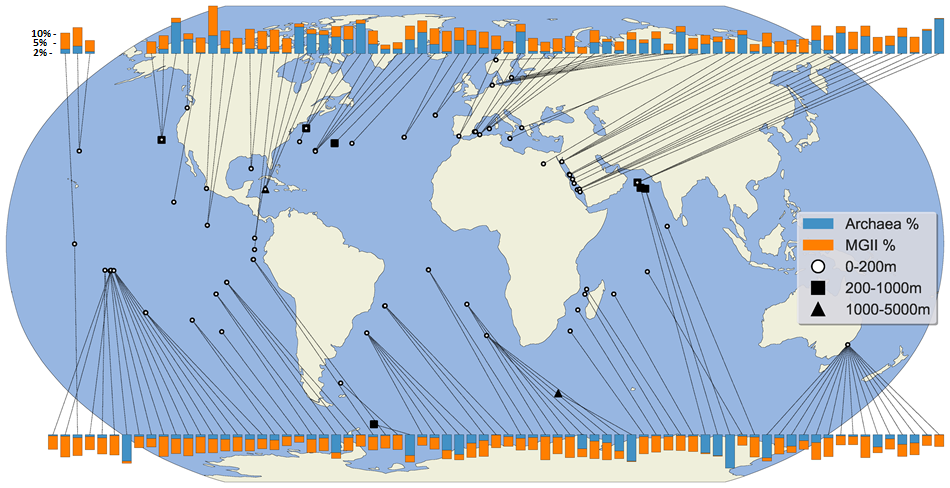
**

**Figure S7 | *Ca*.** *Poseidoniales* **worldwide abundances.** Relative abundance of *Poseidoniales* (MGII) based on SSU rRNA gene community profiles of the 141 metagenomes from which the MAGS were obtained. The bar graphs indicate the relative abundance of *Ca*. Poseidoniales (orange bar; “MGII%”) and all other archaeal groups (blue bar; “Archaea %”) in each sample. Maximum relative abundance of *Ca*. Poseidoniales (MGII) is 100% of all Archaea, and 9.02% of all prokaryotes. Sampling depth is indicated by symbols and explained in the legend.

**
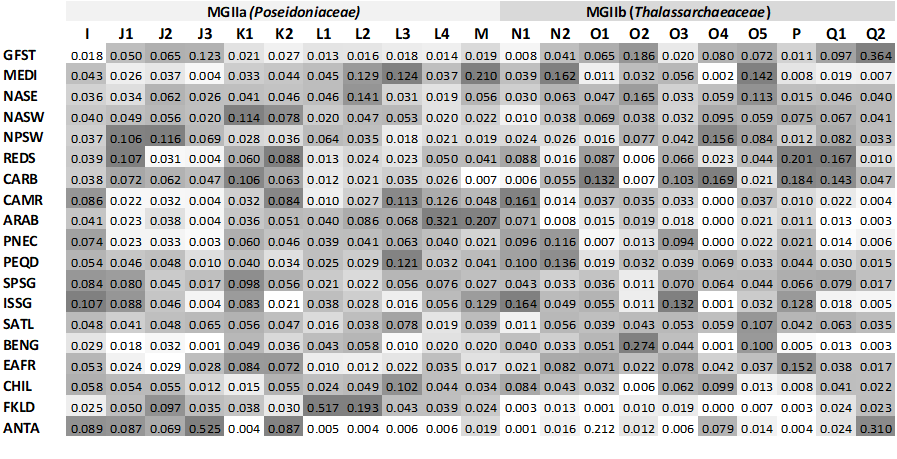
**

**Figure S8 | Genus distribution in biogeographical provinces.** Heatmap displaying the values of the normalised genera abundances across ocean regions (x-axis) versus Ocean regions (y-axis) shown in Fig. 2. Lighter grey shades indicate a lower abundance of a genus, while darker shades indicate a higher abundance, hue is assigned by genus. Note, that only regions with at least one genus showing significantly different abundances from the mean of means (grand mean; baseline) were included in the heatmap. Also based on AIC (Akaike information criterion) model selection, the region “Indian Monsoon Gyres Province” (MONS) was omitted from the sum-to-zero coding based GLM shown in the heatmap. The acronyms for Ocean regions according to Longhurst’s classification (Reygondeau et al. 2013 #1122; [http://www.marineregions.org](http://www.marineregions.org/)) are the following: Gulf Stream (GFST), Mediterranean Sea (MEDI), Northeast Atlantic subtropical gyre (NASE), Northwest Atlantic subtropical gyre (NASW), Northwest Pacific subtropical (NPSW), Caribbean (CARB), Central American coast (CAMR), Northwestern Arabian Upwelling (ARAB), North Pacific equatorial counter current (PNEC), Indian monsoon gyre (MONS), Pacific Equatorial Divergence (PEQD), South Pacific Subtropical Gyre (SPSG), Indian South subtropical gyre (ISSG), South Atlantic gyre (SATL), Benguela Current Coastal (BENG), Eastern India coast (EAFR), Coastal Chile-Peru Current (CHIL), Southwest Atlantic Shelves (FKLD), and Antarctic (ANTA).

**
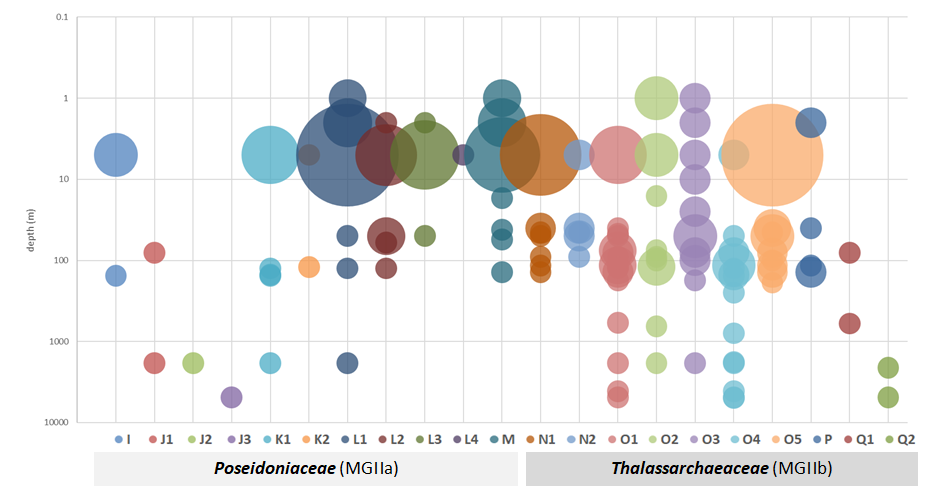
**

**Figure S9 | MAG sampling depths.** The MAG sampling depth, defined as the depth from which the metagenomes were derived, spanned from surface waters to nearly 5000m depth. In many genera MAGs were mostly recovered from surface water samples (<10m). However, genera J2, J3, and Q2 were exclusively recovered from depth below 1000m and genera J1, and Q1 were also absent from surface waters (<10m). MAGs are grouped by genus, and the abundance is shown for each sampling depth (y-axes) whereby the bubble size indicates the number of MAGs.


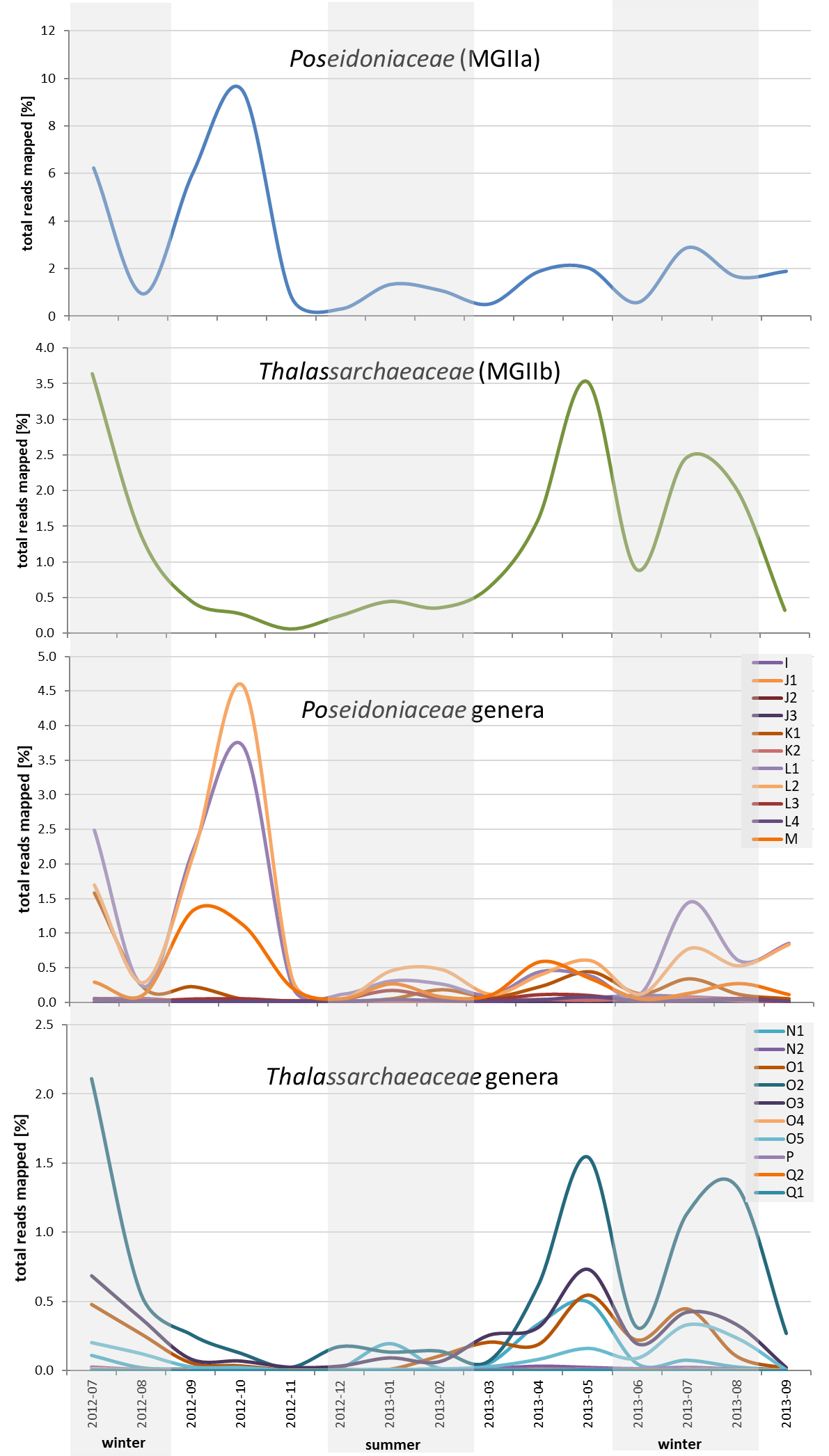


**L2**

**L1**

**M**

**O2**

**O3**

**Figure S10 | Temporal dynamics of *Ca*. Poseidoniales distributions.** The relative abundances of *Ca*. Poseidoniales families and genera, based on read mapping, are shown for each of the 15 months of the Port Hacking time series. The top two graphs show the abundances of the families (*Ca*. Poseidoniaceae (MGIIa) peaking in the Austral spring, and *Ca*. Thalassarchaeaceae (MGIIb) peaking in the Austral autumn and winter. The lower two panels show the relative abundances of the genera within the families, indicating that the *Ca*. Poseidoniaceae spring peak is largely attributable to L1, L2 and M and the *Ca*. Thalassarchaeaceae autumn and winter peaks are mostly due to genus O2. Summer and winter months are highlighted with a grey vertical bar across all graphs.

**
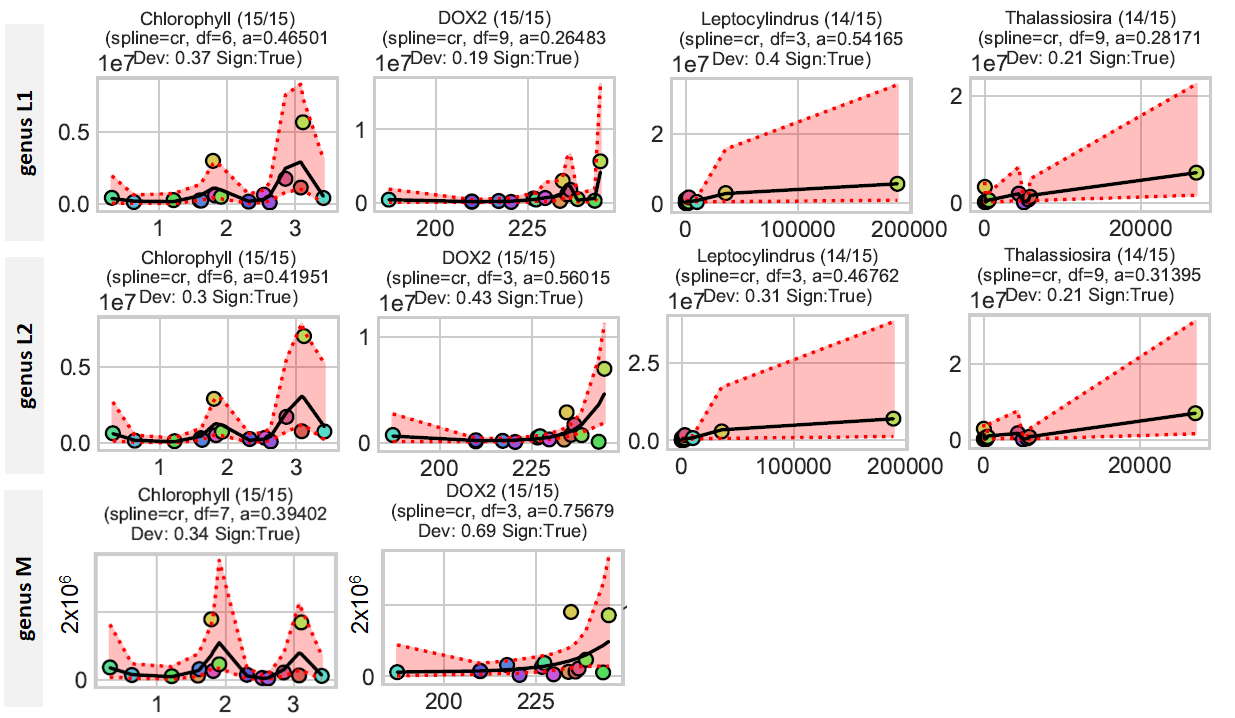
**

**Figure S11 | Relationships between the relative abundance of *Ca***. **Poseidoniales genera and physical, chemical, and biological metadata obtained at the Port Hacking sampling site.** The abundances (y-axes) are plotted against physical, chemical, and biological metadata (x-axes). DOX2 = dissolved oxygen. Abundances are scaled by library size.


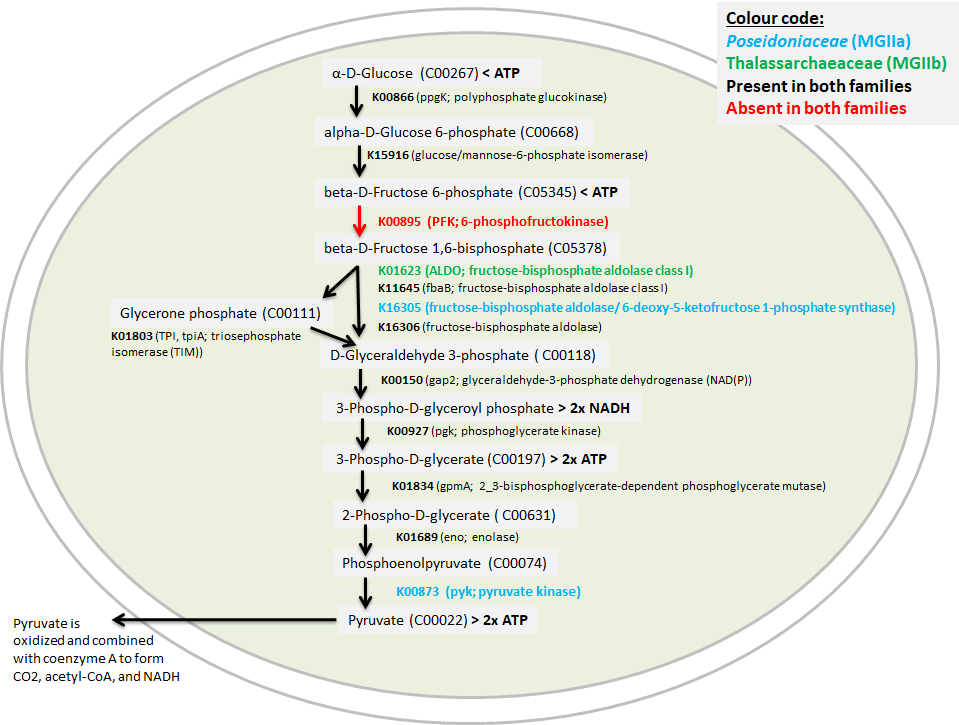


**Figure S12 | Glycolysis pathway.** *Ca*. Poseidoniaceae (MGIIa) and *Ca*. Thalassarchaeaceae (MGIIb) genera encode different genes in the glycolysis pathway. Genes for aldolases, enzymes which convert fructose 1,6 bisphosphate to glyceraldehyde 3-phosphate encoded by, are family specific with *Ca*. Poseidoniaceae encoding K016305 and *Ca*. Thalassarchaeaceae encoding ALDO (K1623*).* The gene encoding pyruvate kinase (*pyk*; K00873), which catalyses the final step of glycolysis, was present in all *Ca*. Poseidoniaceae genera but absent in *Ca*. Thalassarchaeaceae genera with the exception of clade N (**Fig. 4**). In addition, the gene PFK (alternative acronym pfkA), encoding the key glycolysis gene 6-phosphofructokinase [EC:2.7.1.11] was absent from both families which suggests the use of alternative enzymes for this step.


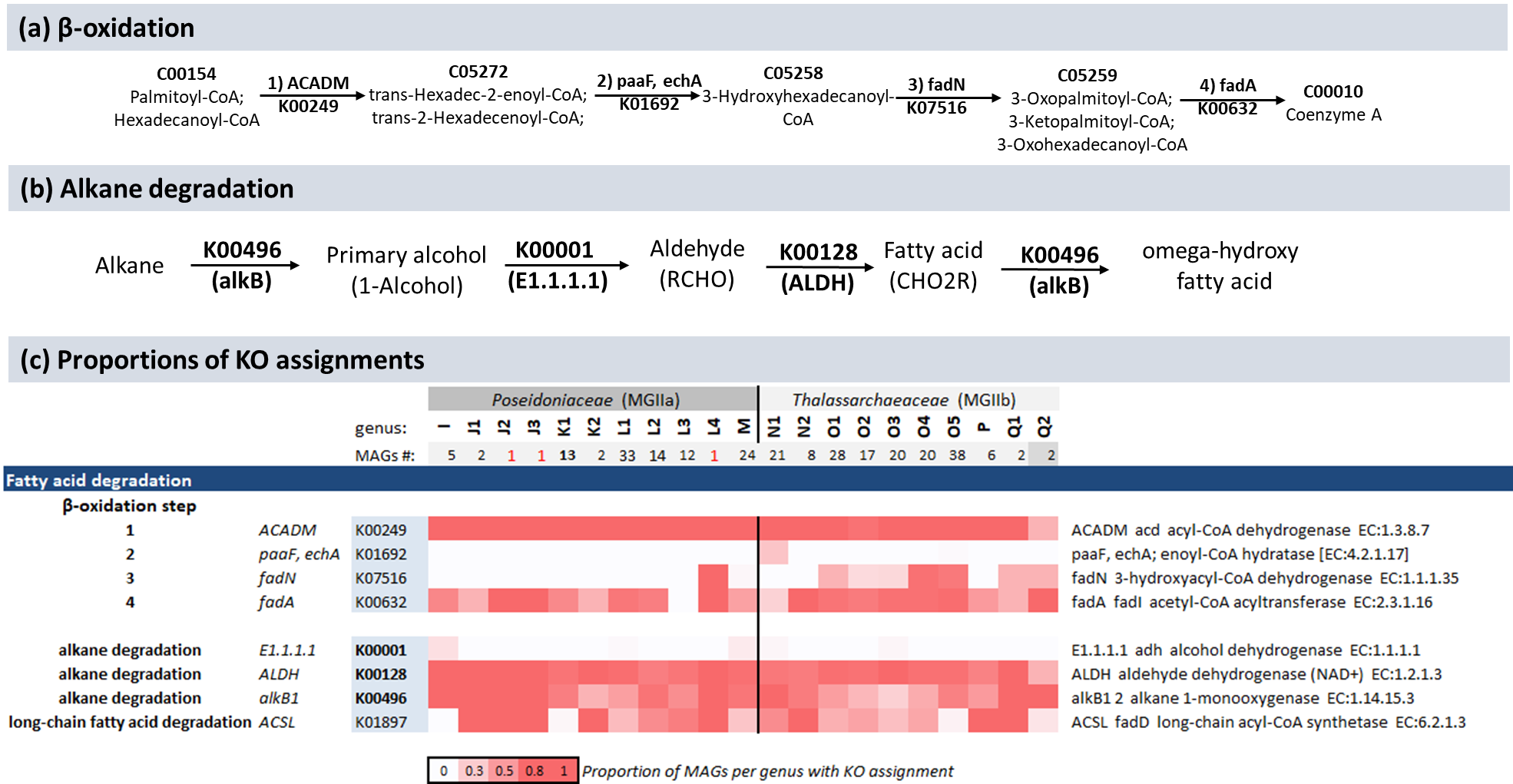


**Figure S13 | Fatty acid degradation genes encoded by *Ca***. **Poseidoniales. (a)** Genes encoding enzymes involved in alkane degradation; **(b)** fatty acid degradation in the β-oxidation pathway; **(c)** Overview of all inferred fatty acid degradation enzymes. Shown is the proportion of MAGs per genus to which a KO (KEGG Orthology) identifier has been assigned.


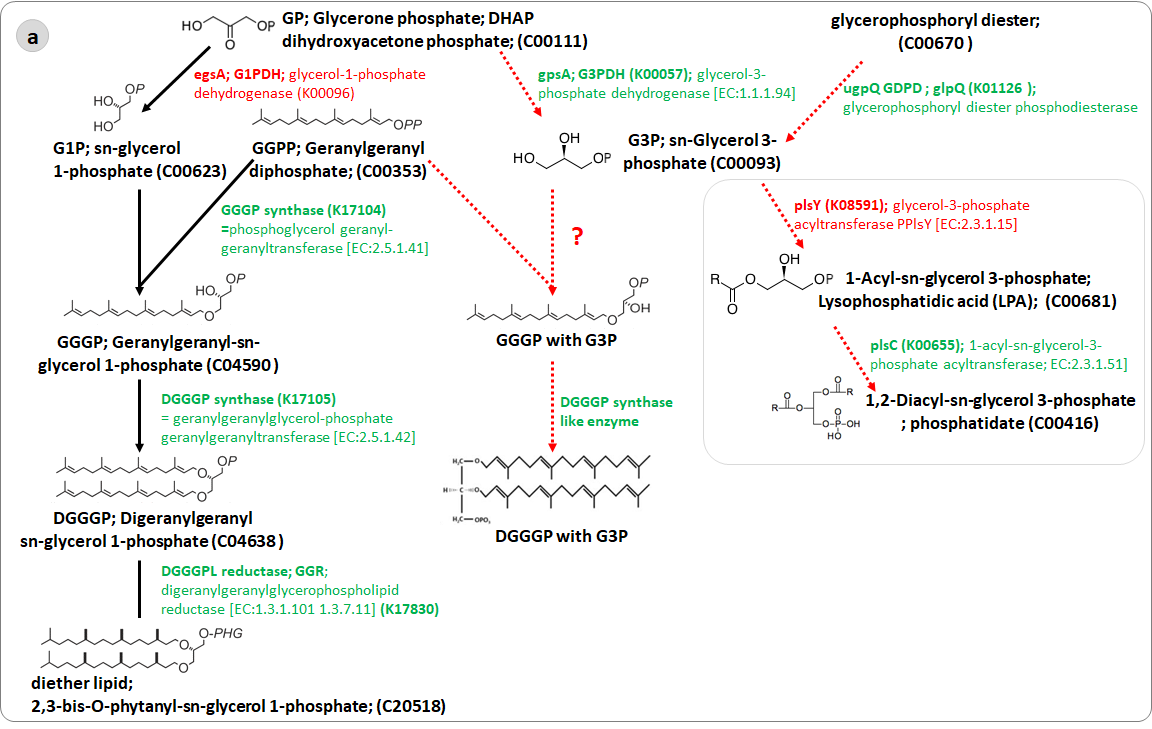


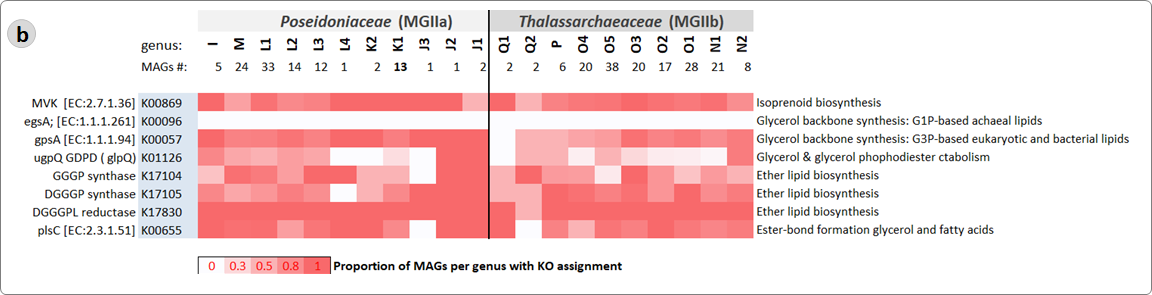


**Figure S14 | Biosynthetic pathway of membrane lipids and glycerol in Ca. Poseidoniales. (a)** Pathway and key enzymes. Enzymes encoded in *Ca*. Poseidoniales are shown in green, enzymes for which genes are missing are shown in red. Dotted red arrows highlight proposed/hypothetical reactions usually only found in bacterial/eukaryotic membrane synthesis. Reactions of a hypothetical bacteria-like fatty acid synthesis are highlighted by a light grey box. KEGG orthology (KO) terms and KEGG compound terms are provided. **(b)** Heatmap showing genes encoding archaeal and bacterial/eukaryotic membrane biosynthesis in Ca. Poseidoniales.

~~
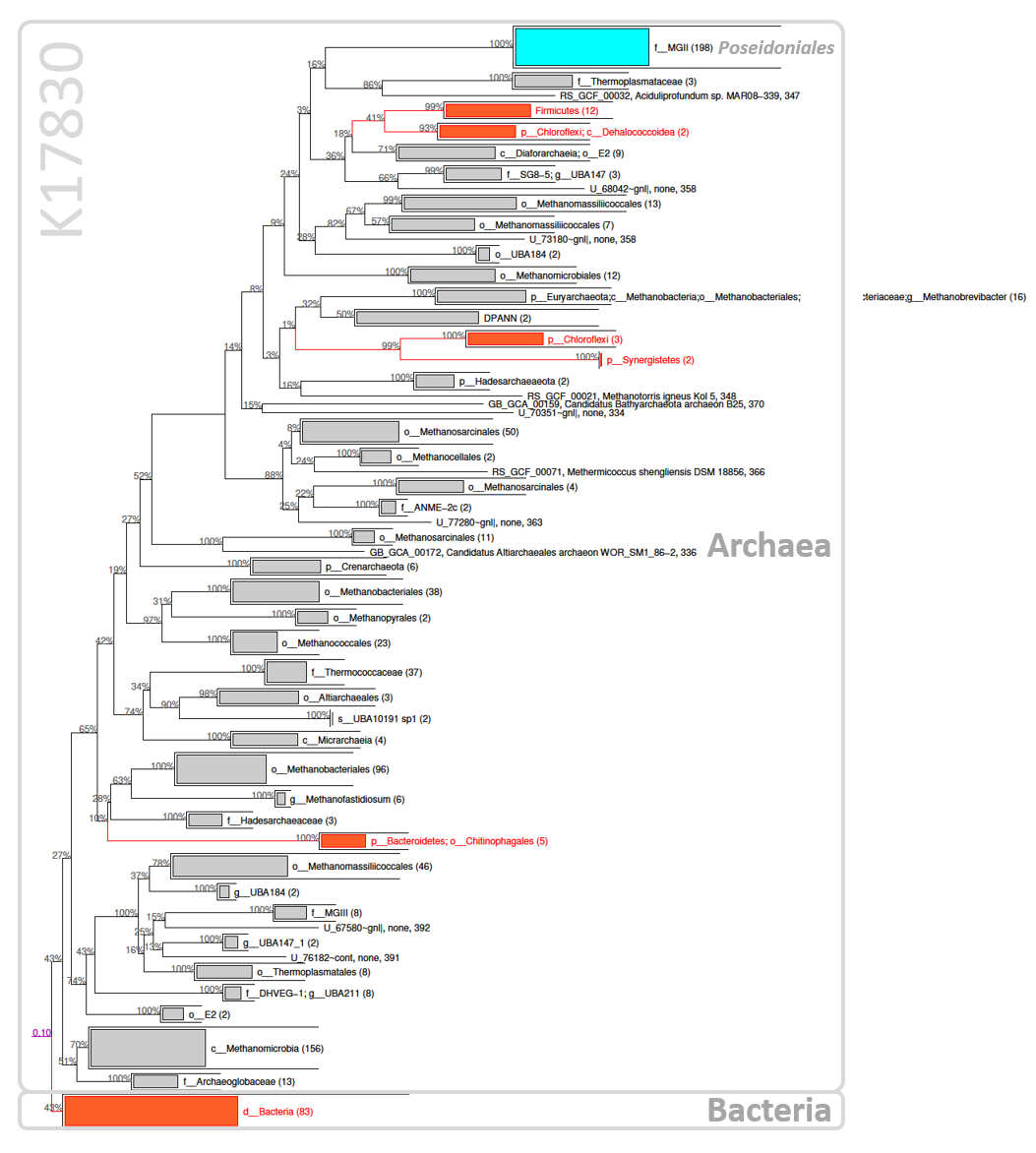
~~

**Figure S15 | Phylogenetic tree of GGR.** Bootstrapped maximum likelihood tree (FastTree, WAG, gamma) of GGR (digeranylgeranylglycerophospholipid reductase, [EC:1.3.1.101 1.3.7.11], K17830). The *Ca*. Poseidoniales homologues cluster together (cyan cluster) in the archaeal radiation of GGR. Clades of homologues assigned to bacterial taxa are highlighted in orange. The phylogenetic signal reveals rampant horizontal gene transfer of GGR among archaeal groups and even across domains.


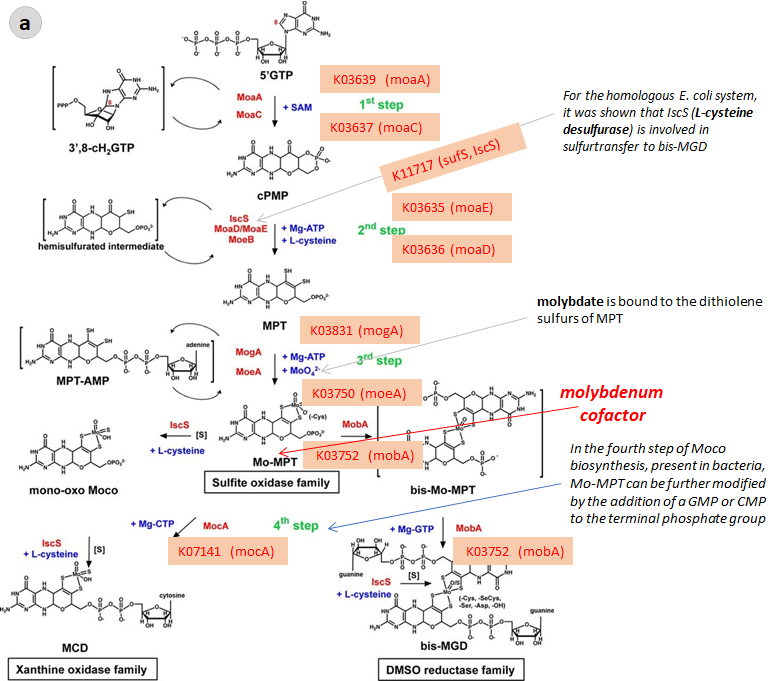


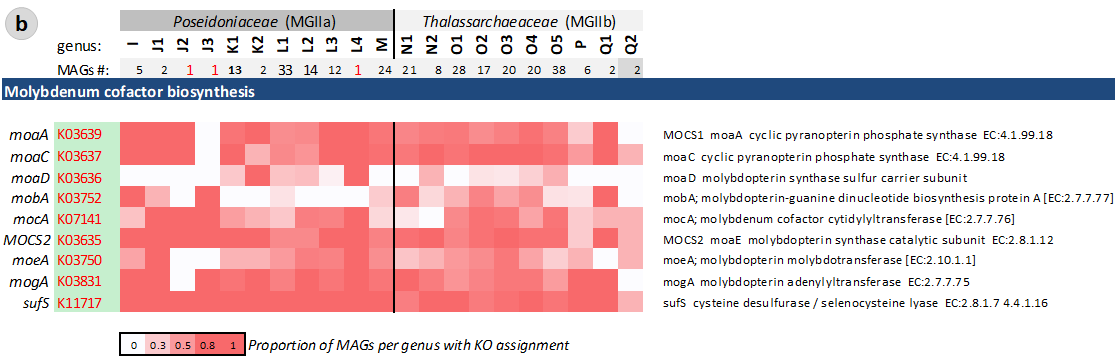


**Figure S16 | Molybdenum cofactor biosynthesis. (a)** The biosynthesis of the molybdenum cofactor occurs in four steps in bacteria and archaea. This includes the formation of (1) cPMP, (2) MPT, (3) Mo-MPT, and (4) the attachment of nucleotides (CMP or GMP) to the phosphate group of MPT, resulting in dinucleotide variants of Moco. *Ca*. Poseidoniales possess all key genes in this pathway. **(b)** Genes present in *Ca*. Poseidoniales. Genes shown in a) are highlighted in red.


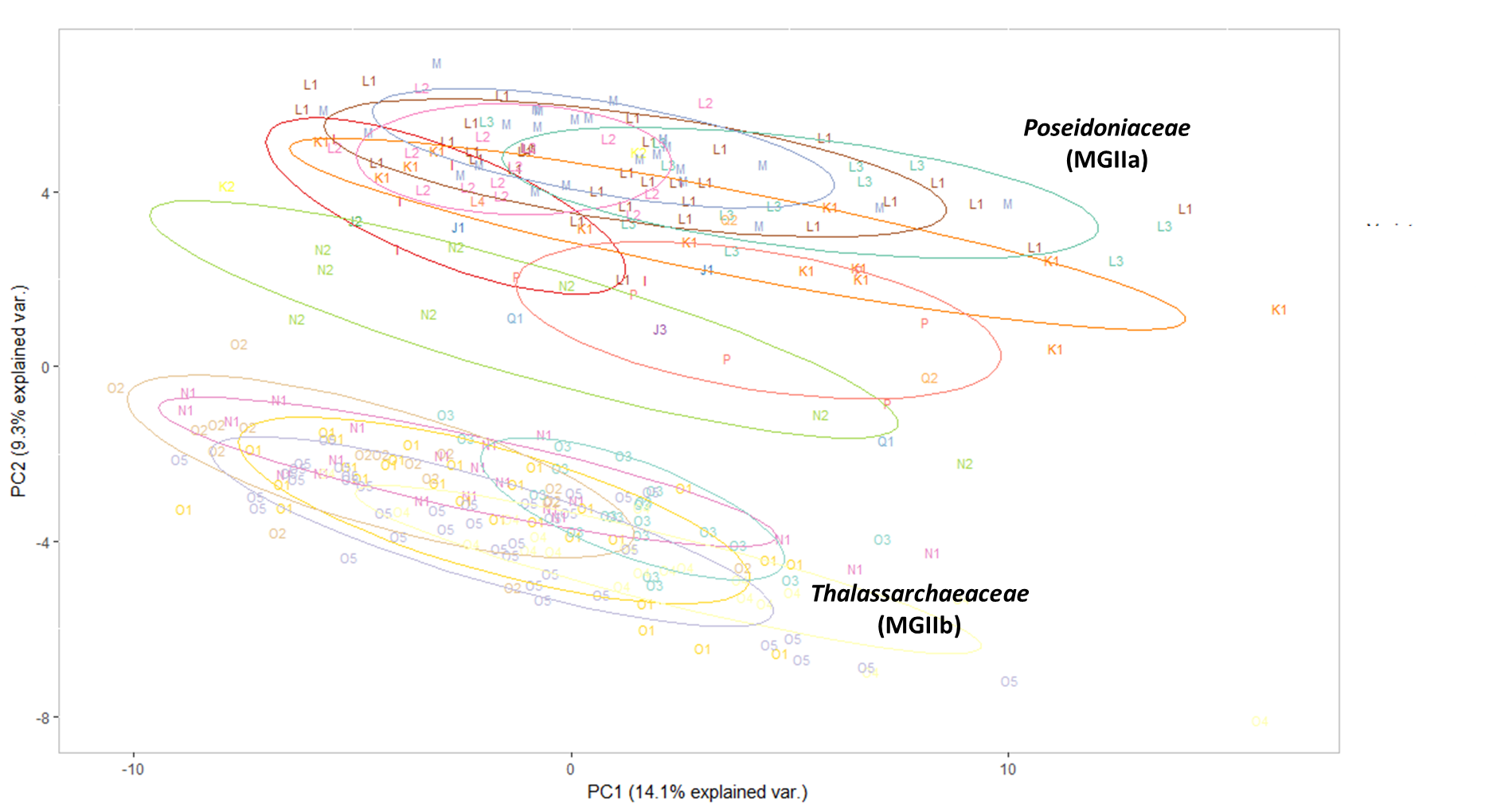


**Figure S17 | Principal component analysis (PCA) of KEGG Orthology (KO) assignments.** The PCA is based on absolute KO counts scaled by library size and includes all genera containing more than 1 MAG. Genera with three or more MAGs are highlighted by an ellipse. The PCA shows a clear separation between the families *Ca*. Poseidoniaceae (MGIIa) and *Ca*. Thalassarchaeaceae (MGIIb) as well as differences between the genera.

**
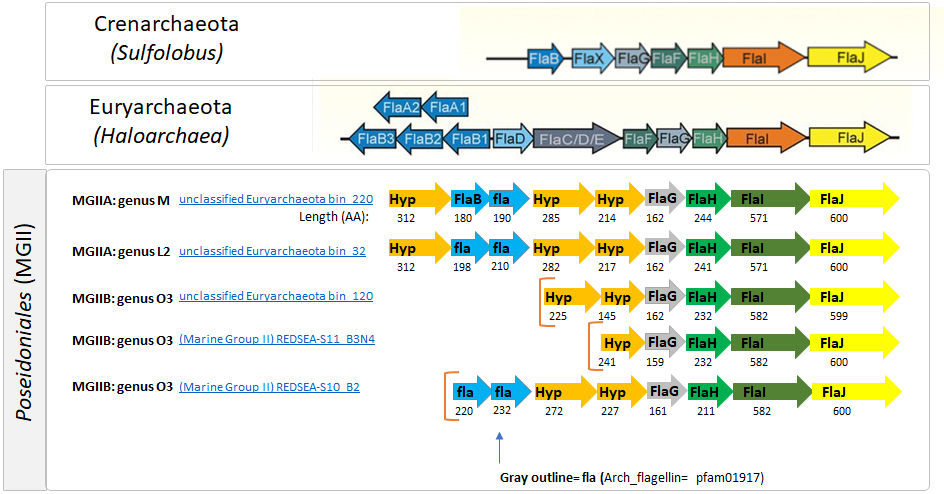
**

**Figure S18 | Flagella operons of Crenarchaeota, Euryarchaeota and selected *Ca***. **Poseidoniales**. Encoded euryarchaeal flagellum proteins include *FlaJ* and *FlaI,* which form the membrane platform of the flagellum structure, and *FlaH*, which is involved in the modulation of *FlaI* activity.


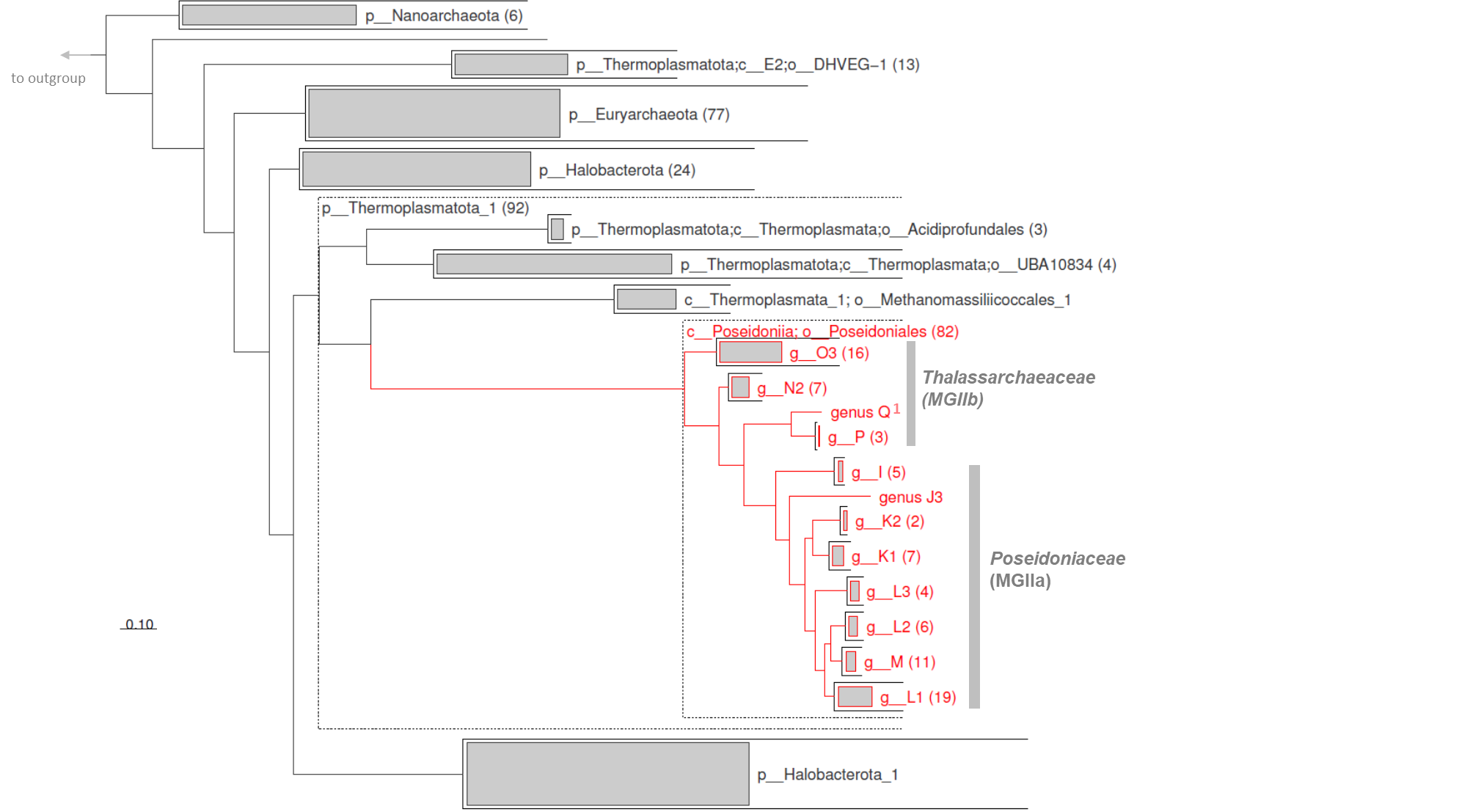


**Figure S19 | Phylogeny of the flagella protein FlaI.** Phylogenetic tree (FastTree, gamma, WAG) of FlaI amino acid alignments. FlaI forms part of the membrane platform of the flagellum structure. *Ca*. Poseidoniales genera are highlighted in red. The scale bar represents 0.1 substitutions per site.


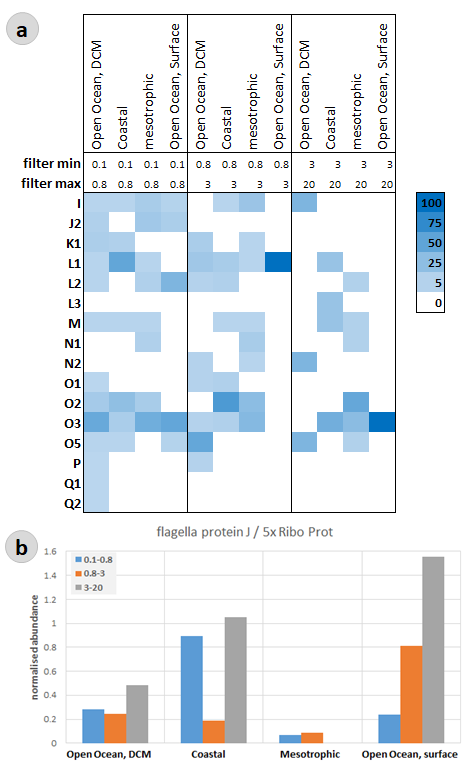


**Figure S20 | Size fractions.** Metagenomic reads were obtained from Orsi et al. 2015 [3]. **(a) Relative abundances of genera in size-fractionated metagenomes.** The heat map shows the relative abundance of *Ca*. Poseidoniales genera (0-100%), based on five ribosomal proteins (S4, S15, S24e, S27ae, L21) which are present in > 92% of all MAGs. Abundances are shown for each marine site (top row). **(b) Flagella protein abundances in size fractioned metagenomes.** The abundances of the flagella protein J were normalised by five ribosomal proteins (S4, S15, S24e, S27ae, L21). Abundances are shown for each marine site and size fractions are colour coded. Marine sites include open ocean samples from the deep chlorophyll maximum layer at 81m (Open Ocean, DCM), coastal samples from Monterey Bay, California taken at 5m depth (Coastal), mesotrophic samples taken 100km of the coast of California at 10m (Mesotrophic), and open ocean samples taken at 5m (Open Ocean, surface).

**
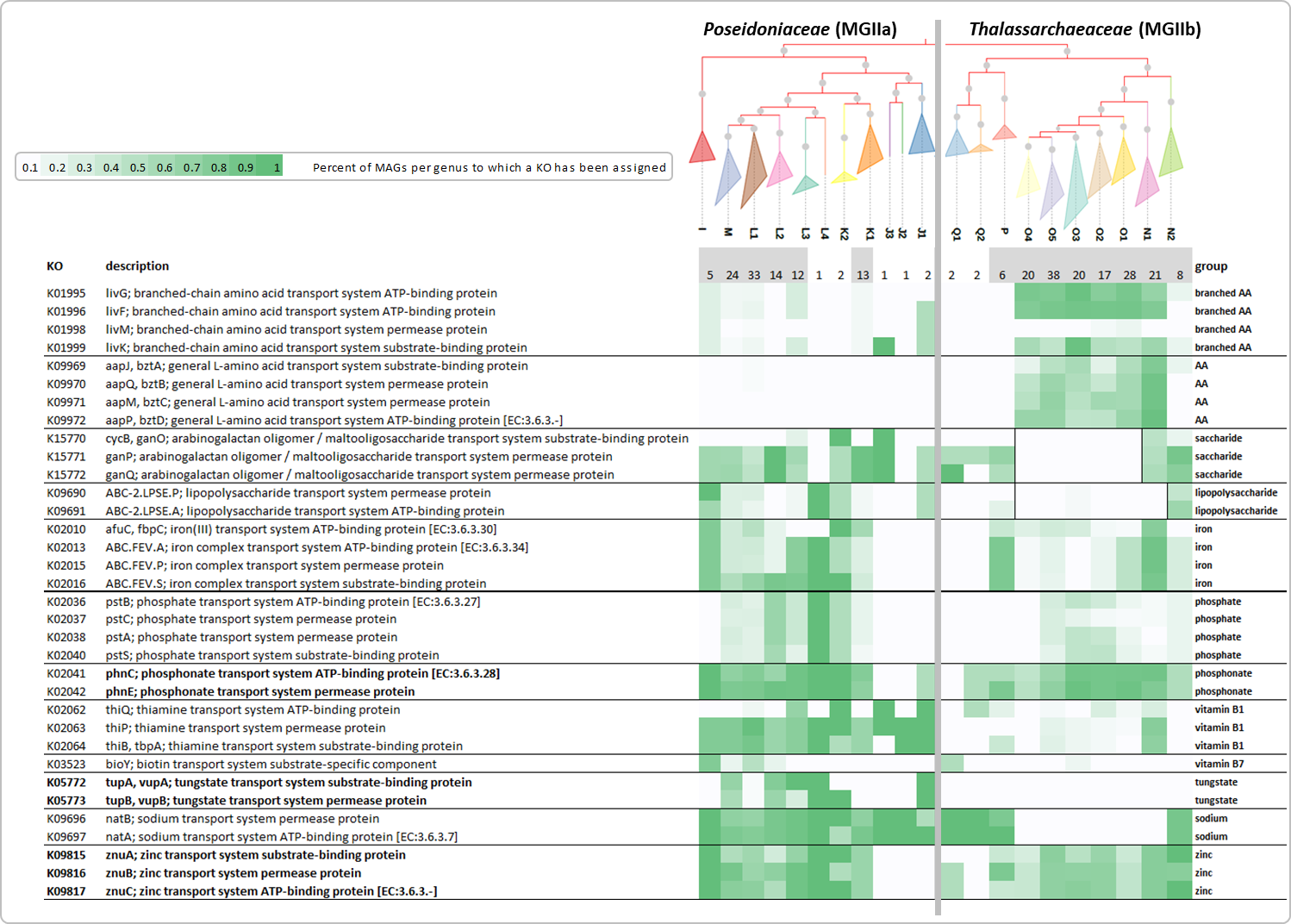
**

**Figure S21 | ATP-binding cassette transporters (ABC transporters) of *Ca***. **Poseidoniales (MGII).** Shown is the percentage of MAGs per genus to which a KO (KEGG Orthology) identifier has been assigned.


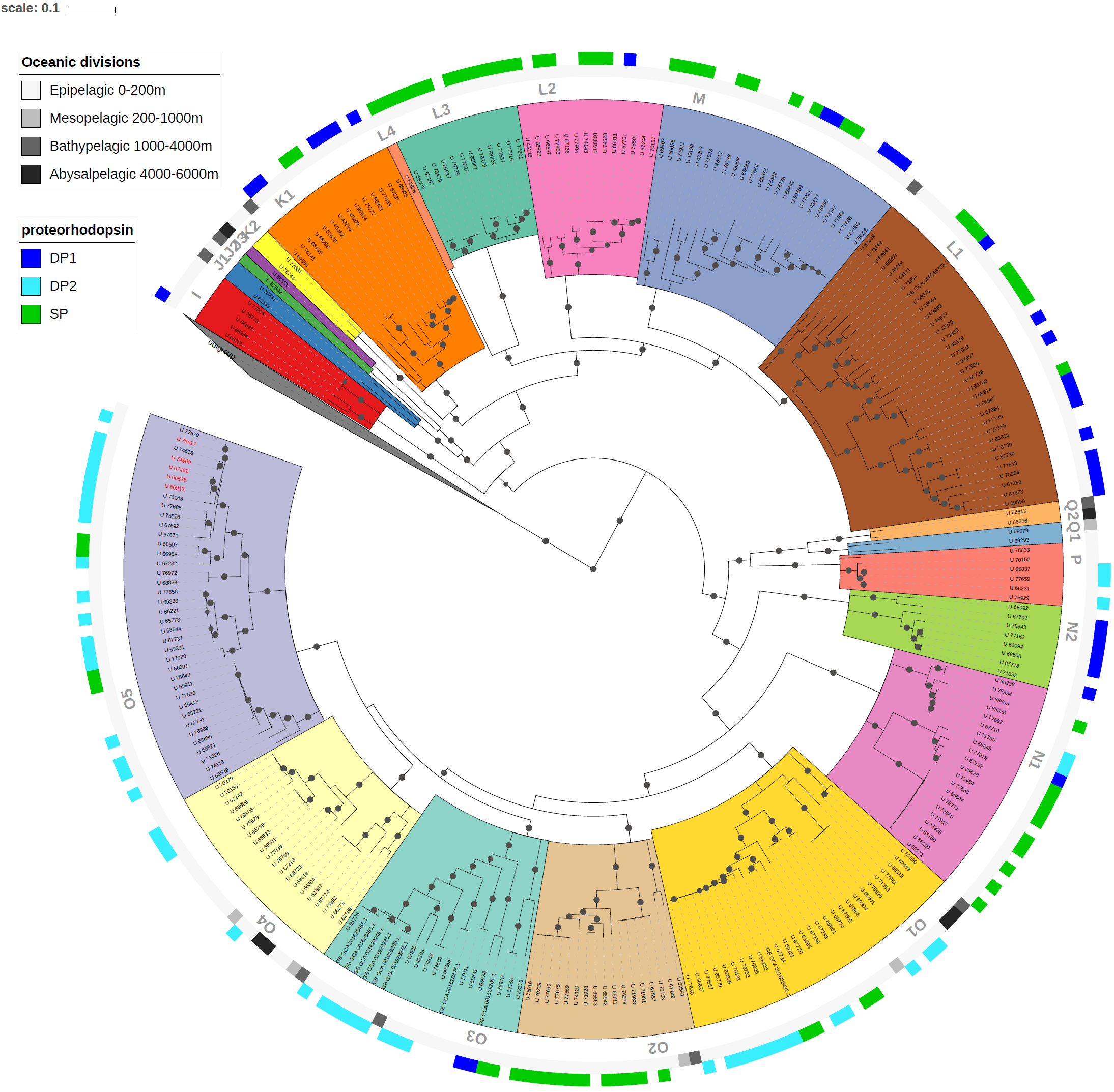


***Poseidoniaceae*** (MGIIa)

***Thalassarchaeaceae*** (MGIIb)

**Figure S22 | Proteorhodopsin genes in *Ca***. **Poseidoniales MAGs.** Phylogenomic tree (FastTree, WAG, gamma) inferred from 122 archaeal marker genes decorated with sampling depth (4 categories, see legend) and proteorhodopsin genes. Abbreviations: DP1 (Deep photic group 1); DP2 (deep photic group 2); SP (shallow photic group.

~~
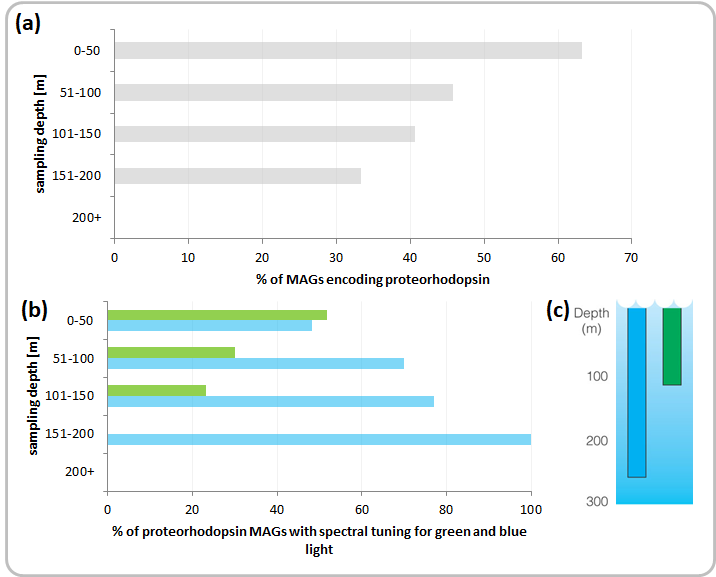
~~

**Figure S23 | Proteorhodopsin genes and light penetration in the open ocean**. **(a)** Percentage of MAGs encoding proteorhodopsin genes, grouped by depth category**. (b)** Percentage of MAGs encoding proteorhodopsin genes with a green or a blue absorption maximum, ordered by depth category. (c) Light attenuation of open ocean water. Different wavelengths are indicated by the corresponding colours. Note the light penetration in coastal waters can be further reduced.


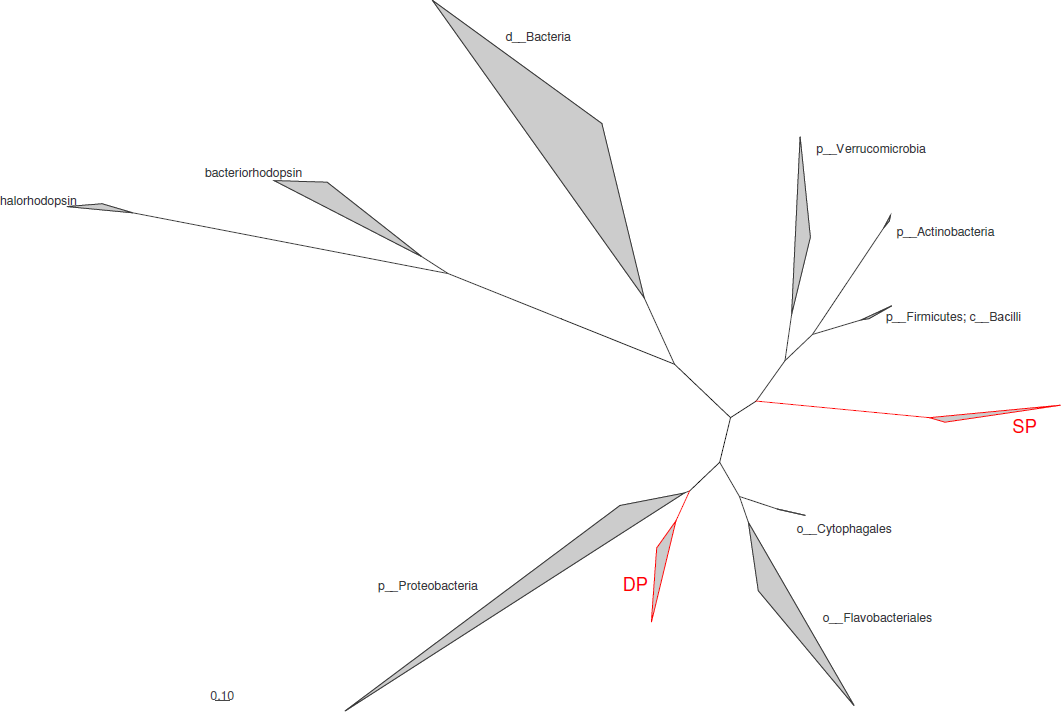


**Figure S24 | Proteorhodopsin phylogeny.** Phylogenetic inference (bootstrapped FastTree, WAG, gamma) of proteorhodopsin (pR) genes. The tree was calculated by applying a 30% similarity filter and an alignment filter including position 113 to 585. *Ca*. Poseidoniales *pR* genes cluster in two clades (highlighted in red), which we termed clade DP (deep photic) and clade SP (shallow photic). Halorhodopsin and bacteriorhodopsin genes were used as outgroup. The scale bar represents 0.1 substitutions.


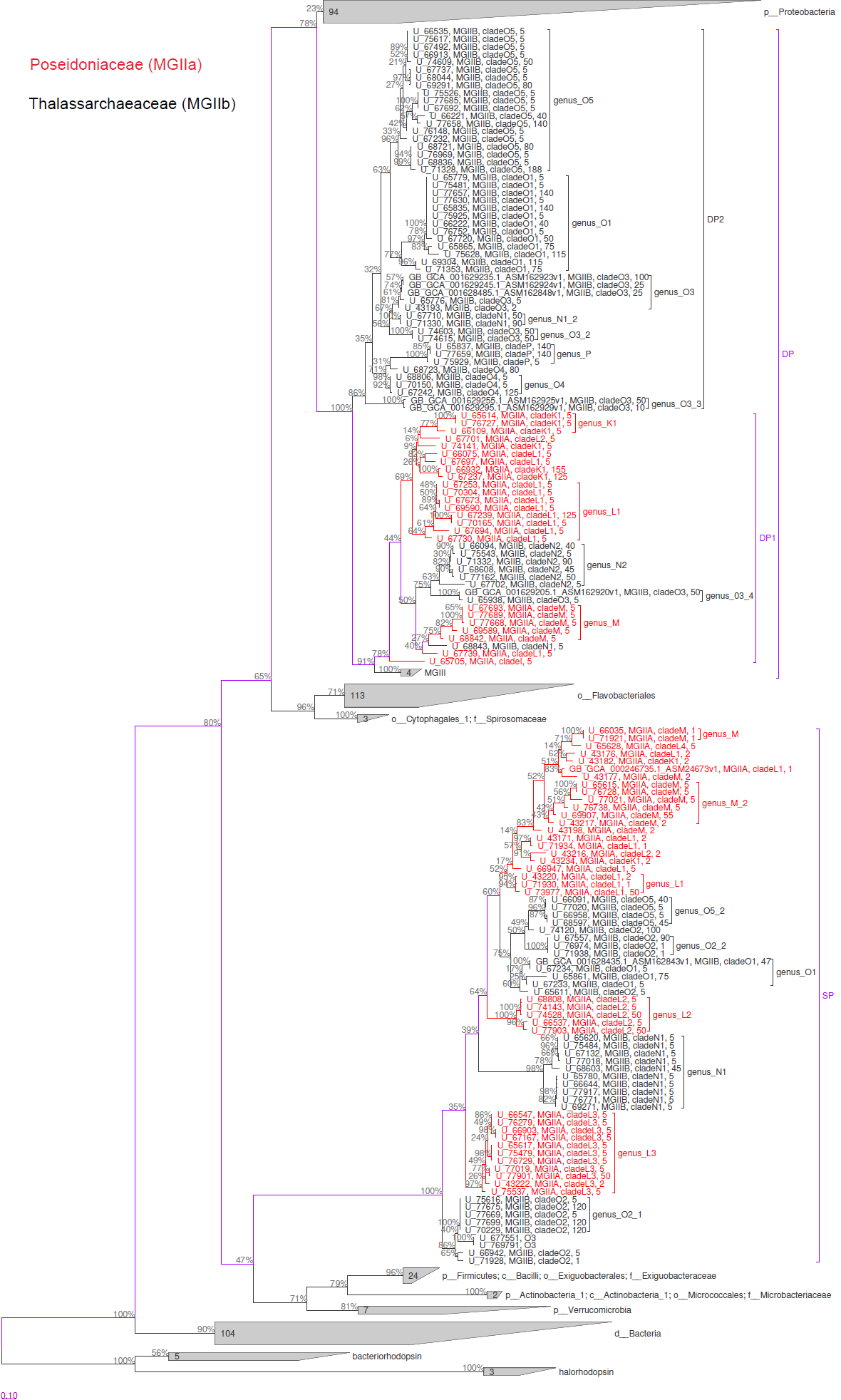


**Figure S25 | Detailed proteorhodopsin phylogeny.** Phylogenetic inference (bootstrapped FastTree, WAG, gamma) of proteorhodopsin genes. The tree was calculated applying a 30% similarity filter and an alignment filter including position 113 to 585. *Ca*. Poseidoniales *pR* genes cluster in two clades (highlighted in red), which we termed clade DP (deep photic) and clade SP (shallow photic). Clade DP is further divided into two subclades DP1 and DP2, the latter contains only *Ca*. Thalassarchaeaceae (MGIIb) genera. Genes (taxa) in clade DP and SP are colour coded according to their phylogenomic classification: *Ca*. Poseidoniaceae (MGIIa) taxa are shown in red font and *Ca*. Thalassarchaeaceae (MGIIb) taxa in black font. Halorhodopsin and bacteriorhodopsin genes were used as outgroup. The scale bar represents 0.1 substitutions.


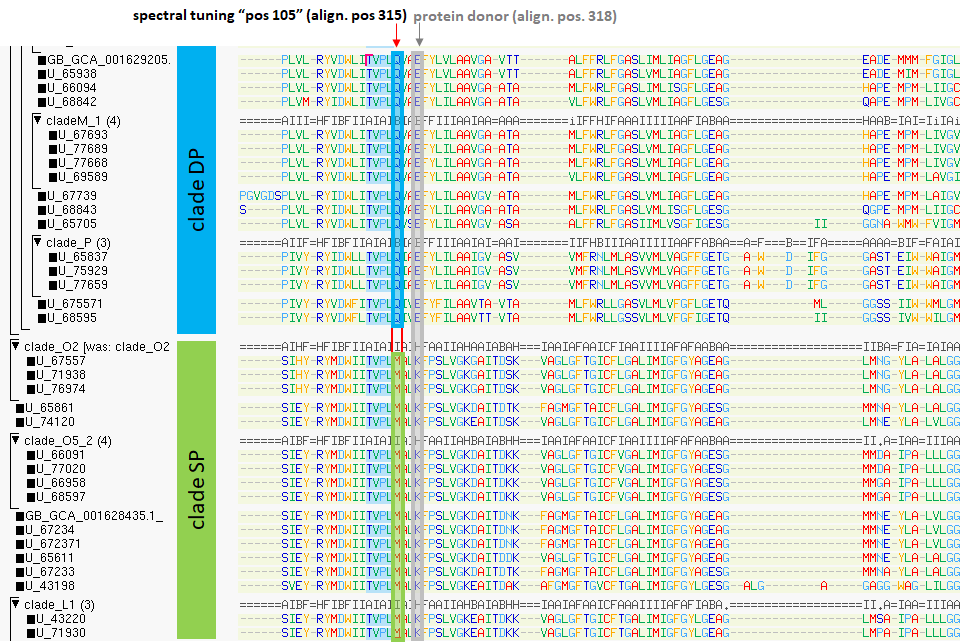


**Figure S26 | Proteorhodopsin gene alignments.** Examples of proteorhodopsin (pR) gene alignments including clade DP (deep photic) and clade SP (shallow photic). The position responsible for spectral tuning, alignment position 315 (highlighted by a red arrow), is Q (Glutamine, Gln) absorbing blue light in clade DP, and M (methionine) absorbing green light in clade SP. Alignment position 318 is the protein donor amino acid (highlighted with a grey bar). Clade DP encodes glutamic acid (Glu, E) at this position and clade SP encodes lysine (Lys, K).


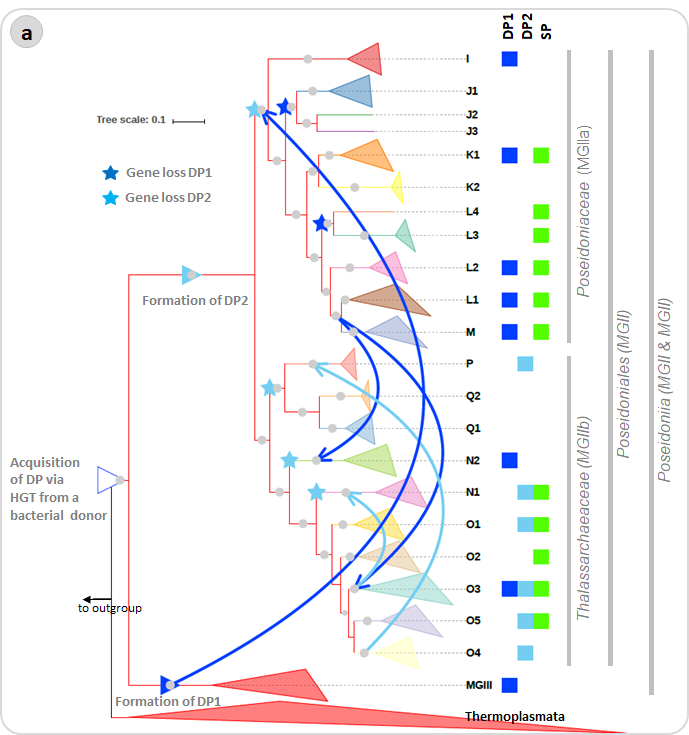


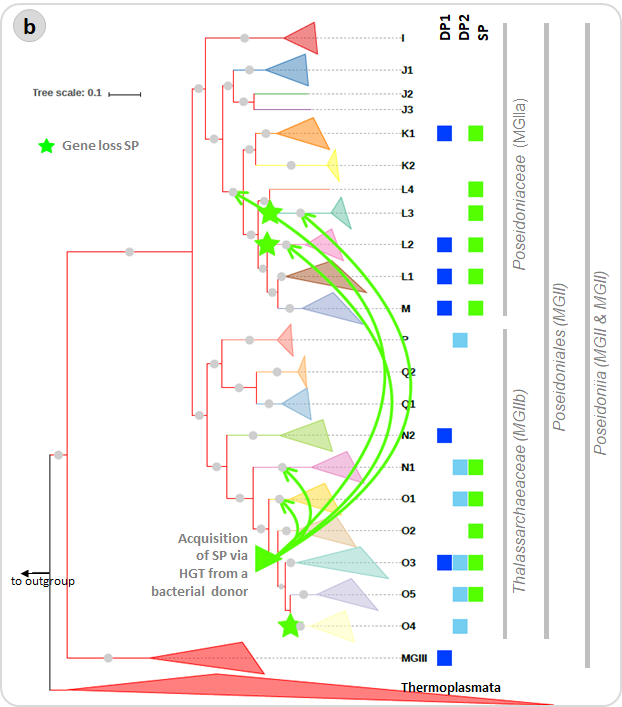


**Figure S27 | Inferred horizontal gene transfers of proteorhodopsin genes.** Phylogenomic trees calculated from 122 archaeal marker genes with *Ca*. Poseidoniales MAGs collapsed on the genus level. **(a) Horizontal gene transfers (HGTs) of clade DP (deep photic).** After the initial acquisition form a bacterial donor the DP proteorhodopsin (pR) gene evolved into DP1 and DP2. The former was (and is) present in MGIII, has been transferred via HGT to *Ca*. Poseidoniaceae and from there into some *Ca*. Thalassarchaeaceae genera. The DP2 *pR* gene was lost in the ancestor of *Ca*. Poseidoniaceae and in several *Ca*. Thalassarchaeaceae genera. Further HGT between *Ca*. Thalassarchaeaceae genera allowed each genus, except for the deep-water adapted genera Q1 and Q2 and genus N2, to maintain the DP2 *pR* gene. **(b) Horizontal gene transfers (HGTs) of clade SP (shallow photic).** The SP proteorhodopsin (pR) gene was initially acquired in the *Thalassarchaeaceae* through HGT from a bacterial donor and spread from there into *Ca*. Poseidoniaceae via several other HGTs. The loss of a *pR* gene variant is indicated by a star, colour coded in dark blue (clade DP), light blue (clade DP2), and green (clade SP). Horizontal gene transfers (HGs) are indicated by arrows following the same colour scheme. The scale bar represents 0.1 substitutions.

**
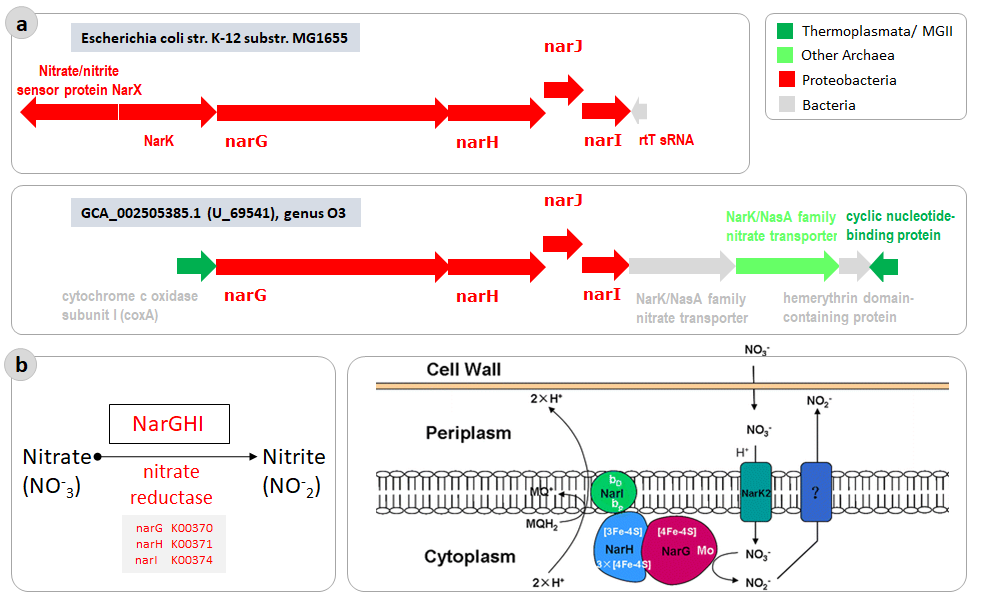
**

**Figure S28 | Nitrate uptake and reduction.** The narGHI operon encodes nitrate reductase 1 consisting of the membrane-bound respiratory NarGHI complex. **(a) Gene neighbourhoods.** Similar to *Escherichia coli*, *Ca*. Poseidoniales MAGs possess genes encoding all three subunits: *narG* (alpha subunit), *narH* (beta (Fe-S) subunit), and *narI* (gamma (cytochrome b(NR)) subunit). Also, the *narJ* gene is present, which encodes the chaperone required for proper molybdenum cofactor insertion and final assembly of the nitrate reductase 1. **(b) Encoded function**. Nitrate reductase 1 mediates the conversion of Nitrate (NO^-^_3_) to Nitrite (NO^-^_2_) and the enzyme complex is anchored in the cell membrane. Illustration of the NarGHI complex modified from Huang et al. 2015 [4];<https://link.springer.com/article/10.1007/s00284-015-0838-2> .


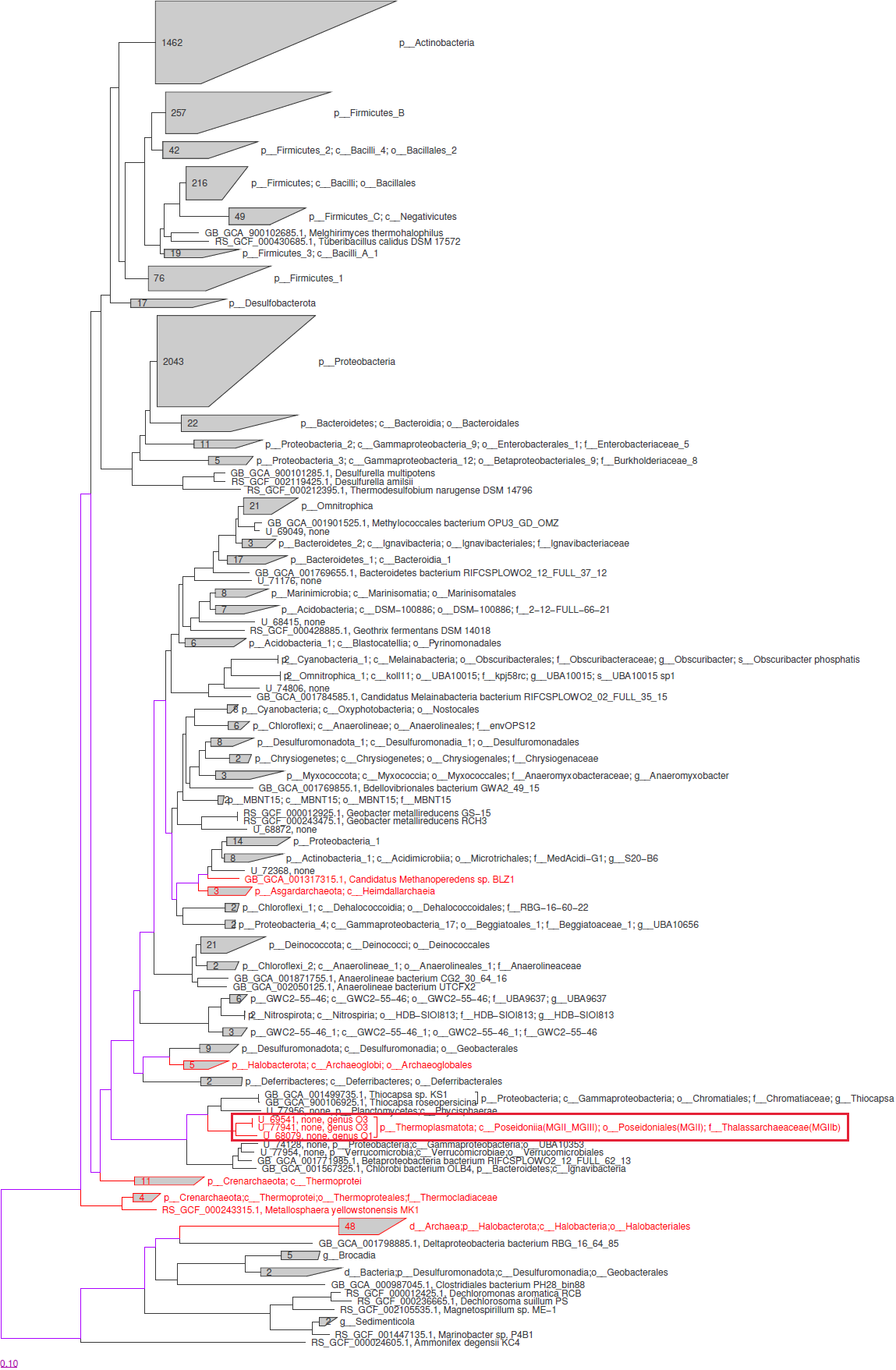


**Figure S29 | Phylogenetic inference of narG.** The *narG* gene encodes the nitrate reductase alpha subunit. Phylogenetic tree (FastTree, gamma, WAG) was calculated from amino acid sequence alignments of *narG*. Archaeal homologues are highlighted in red, *Ca*. Poseidoniales homologues are marked with a red box. The scale bar represents 0.1 substitutions.


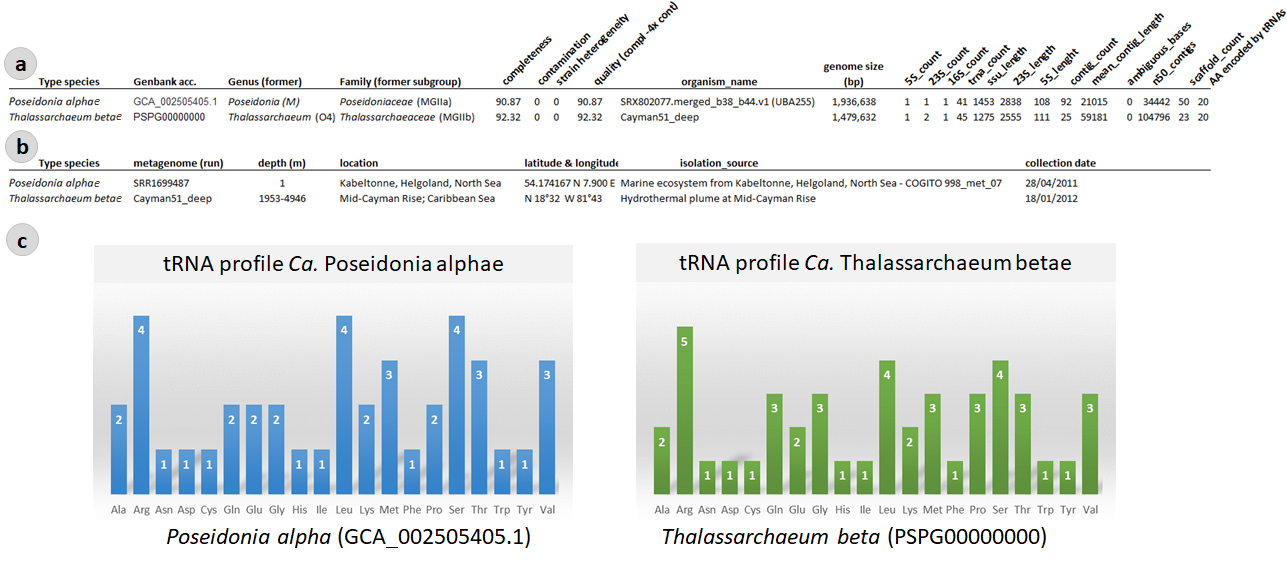


**Figure S30 | Type species.** In accordance with recent proposals to designate type material based on metagenome-assembled genomes (MAGs), we propose the type species *Ca*. Poseidonia *alphae* (fam. nov. *Poseidoniaceae*; MGIIa) and *Ca*. Thalassarchaeum *betae* (fam. nov. T*halassarchaeaceae*; MGIIb). (a) Genome characteristics, (b) environmental metadata, and (c) encoded tRNAs for each amino acid are shown for both type species.

**
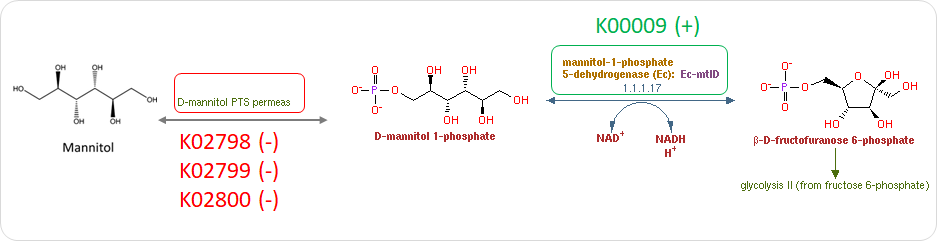
**

**Figure S31 | Degradation of Mannitol.**  The mannitol degradation pathway with the key enzymes D-mannitol PTS permease and mannitol-1-phosphate 5-dehydrogenase is shown. Genera J1, J2, J3 P, and Q1 possess homologous of mtlD encoding mannitol-1-phosphate 5-dehydrogenase (K00009; [EC:1.1.1.17]; highlighted in green). However, genes encoding D-mannitol PTS permease (highlighted in red), converting mannitol to mannitol-phosphate, were not present in these genera. Pathway illustration modified from BIOCYC (<https://biocyc.org/META/NEW-IMAGE?type=PATHWAY&object=MANNIDEG-PWY&detail-level=3>).

**References**

1. Bowers RM, Kyrpides NC, Stepanauskas R, Harmon-Smith M, Doud D, Reddy TBK, et al. Minimum information about a single amplified genome (MISAG) and a metagenome-assembled genome (MIMAG) of bacteria and archaea. *Nat Biotech* 2017; **35**: 725–731.

2. Castelle CJ, Wrighton KC, Thomas BC, Hug LA, Brown CT, Wilkins MJ, et al. Genomic Expansion of Domain Archaea Highlights Roles for Organisms from New Phyla in Anaerobic Carbon Cycling. *Current Biology* 2015; **16**: 690–701.

3. Orsi WD, Smith JM, Wilcox HM, Swalwell JE, Carini P, Worden AZ, et al. Ecophysiology of uncultivated marine euryarchaea is linked to particulate organic matter. *ISME J* 2015; **9**: 1747–1763.

4. Huang Q, Abdalla AE, Xie J. Phylogenomics of Mycobacterium Nitrate Reductase Operon. *Curr Microbiol* 2015; **71**: 121–128.
